# Establishment of immune prognostic signature and analysis of prospective molecular mechanisms in childhood osteosarcoma patients

**DOI:** 10.1097/MD.0000000000023251

**Published:** 2020-11-13

**Authors:** Zide Zhang, Chong Liu, Tuo Liang, Chaojie Yu, Zhaojie Qin, Xin Zhou, Jiang Xue, Haopeng Zeng, Zhaojun Lu, Guoyong Xu, Zequn Wang, Jiarui Chen, Jie Jiang, Xinli Zhan

**Affiliations:** a Guangxi Medical University; b Spine and Osteopathy Ward, The First Affiliated Hospital of Guangxi Medical University, Nanning, Guangxi, People's Republic of China.

**Keywords:** C-C motif chemokine ligand genes, C-C motif chemokine receptor, childhood osteosarcoma, immune infiltration, immune prognostic signature, molecular mechanisms

## Abstract

**Background:**

In pediatric tumors, immunotherapy exhibits less toxicity than chemotherapy and radiation. The current study aims to identify potential immune targets in immune-related genes of C-C motif chemokine ligand genes (CCLs) and C-C motif chemokine receptors (CCRs) in childhood osteosarcoma (OS) and to explore the underlying molecular mechanisms of childhood OS.

**Methods:**

Firstly, we identified immune-related genes in CCLs and CCRs, these genes were used for functional annotation and interaction analysis. Then, the prognostic value of these genes was evaluated using Kaplan-Meier analysis and multivariate COX regression model. And the potential relationship between risk score and immune infiltrating cells was identified. Finally, gene set enrichment analysis was used to determine the underlying molecular mechanism of OS. Immune-related genes in CCLs and CCRs are inextricably linked.

**Results:**

The results of survival analysis of these genes show that CCL5, CCL8, CCR4, and CCR5 are significantly associated with the prognosis of childhood OS. The combined effect survival analysis shows that the co-high expression of these 4 genes has a good prognosis for childhood OS. A prognostic signature model was constructed based on the 4 genes mentioned above, and the result of time-dependent receiver operating characteristic curves showed that this model was a good predictor of childhood OS 3- and 5-year prognosis. In addition, the risk score of the constructed prognostic signature model was closely related to immune infiltration. We also found that CCL5, CCL8, and CCR5 may affect the prognosis of OS through complex regulation among Toll-like receptor signaling pathway, mitogen-activated protein kinase (MAPK) family signaling cascade, and nuclear factor-kappaB pathway, whereas CCR4 affects the prognosis of OS by regulating eukaryotic translation.

**Conclusion:**

CCL5, CCL8, CCR4, and CCR5 are potential prognostic markers for the prognosis of childhood OS, and the underlying molecular mechanisms of childhood OS have been identified.

## Abstract 2

Supplemental Digital Content is available in the text

## 1 Introduction

Osteosarcoma (OS) is the most common solid malignant disease in childhood (an annual incidence of 5.6 cases per million children). OS was rarely diagnosed before age of 5, and the incidence rate reached the peak with age until 10 to 14 years old. The second peak of the age distribution of OS occurred over 50 years old.^[1–4]^ At present, the treatment of OS is mainly based on resection of primary lesions and combination of multiple chemotherapies. The overall survival rate of OS was significantly improved after systematic treatment.^[5,6]^ However, OS is prone to metastasis in the early stage, and about 30% of patients die of lung metastasis even within 5 years after diagnosis.^[7,8]^ In addition, drug resistance worsens the prognosis of OS.^[9]^ Therefore, it is essential to identify early diagnostic markers and therapeutic targets for OS. Based on the encouraging results of immunotherapy and gene therapy in OS,^[10,11]^ the current study aimed to identify new immune targets associated with prognosis.

“Cancer Immunotherapy” was defined as Breakthrough of the Year 2013.^[12]^ The interactions between the immune system and cancer include immune surveillance, immune cell infiltration, and tumor cytolysis. Immunotherapy counteracts the immune escape of tumors by targeting the tumor microenvironment and reactivates the patient's immune system to achieve the goal of recognizing and eliminating tumor cells.^[13,14]^ In pediatric tumors, immunotherapy exhibits less toxicity than chemotherapy and radiation. The aim of the current study was to identify prognostically relevant immune targets for childhood OS.

A large amount of evidence indicates that C-C motif chemokine ligand genes (CCLs) play an important role in the development, progression, and metastasis of cancers, such as gastric cancer,^[15]^ colorectal cancer,^[16]^ breast cancer,^[17]^ and oral squamous cell carcinoma.^[18]^ C-C motif chemokine receptors (CCRs) are the key mediator of inflammation and immune response, and have significant association with multiple cancers.^[19,20]^ Nevertheless, the relationship between CCLs, CCRs and the prognosis of childhood OS is still uncertain. Therefore, the aim of the current study was to identify immune-related genes in CCLs and CCRs, and to explore the relationship between these genes and prognosis of childhood OS.

## 2 Materials and methods

### 2.1 Gene expression datasets and immune-related genes

The gene expression quantification data of 88 childhood OS samples were of the HTSeq-FPKM type, and downloaded from the University of California, Santa Cruz Xena (UCSC Xena; http://xena.ucsc.edu/) on December 20, 2019. The corresponding clinical data were downloaded from UCSC Xena at the same time. Immune-related genes were downloaded from the Immunology Database and Analysis Portal (ImmPort) database (https://www.immport.org/). This study was based on data from an open database. Ethics and patient consent are not applicable.

### 2.2 Bioinformatics analysis of immune-related genes in CCLs and CCRs

To learn more about the functionality of immune-related genes in CCLs and CCRs, we use the Database for Annotation, Visualization and Integrated Discovery (DAVID; https://david.ncifcrf.gov/; version 6.8)^[21]^ to perform gene ontology^[22]^ and Kyoto Encyclopedia of Genes and Genomes (KEGG)^[23]^ pathway on these genes. And then, a protein–protein interaction network was constructed using the Search Tool for the Retrieval of Interacting Genes (STRING; https://string-db.org/; version 11)^[24]^ to analyze the correlation between these genes. Finally, a gene-gene interaction network was constructed using GeneMANIA (http://genemania.org/).^[25]^

### 2.3 Survival and correlation analysis

According to gene expression level, patients were divided into high- group and low-expression group, and then different genes were analyzed for survival (Kaplan-Meier analysis with log-rank test). According to the results, the correlation between genes with significant significance for childhood OS prognosis was analyzed with the Pearson correlation coefficient in R (http://cran.r-project.org/; version 3.6.0) using *corrplot* package. Then the prognosis-related genes were analyzed for combined effect survival (Kaplan-Meier analysis with log-rank test), and a nomogram was constructed. The nomogram was constructed by *rms* package in R according to the expression of prognosis-related genes.

### 2.4 Prognostic signature construction

According to the survival analysis results of the above genes, the genes significantly related to the prognosis of childhood OS were combined to construct a prognostic model based on gene expression level. The formula for the risk score was as follows: risk score = (expression value of gene A) × β A + (expression value of gene B) × β B + …(expression value of gene n) × β n; β meant the regression coefficient.^[26]^ In addition, the *survival* receiver operating characteristic (ROC) package of R software was used to generate time-dependent ROC curves to test the predictive performance of prognostic features.

### 2.5 Correlation analysis between significant genes expression, risk score, and tumor-infiltrating immune cells

CIBERSORT is a deconvolution algorithm based on gene expression. We downloaded gene expression feature matrix of 22 immune cells from CIBERSORT platform(https://cibersort.stanford.edu/), and then evaluate tumor-infiltrating immune cells of childhood OS samples with CIBERSORT deconvolution algorithm. Finally, the correlation between significant gene expression, risk score of the model, and tumor-infiltrating immune cells was analyzed.

### 2.6 Gene set enrichment analysis (GSEA)

To further explore the biological pathways of enrichment of these significant survival significance genes, we uploaded the relevant data required for these genes to GSEA (version 4.0.3),^[27]^ and then used the databases of c2 and c5 in the Molecular Signature Database (MSigDB) (https://www.gsea-msigdb.org/)^[28]^ for enrichment analysis. The enrichment gene sets in the GSEA that attained a false discovery rate (FDR) of <0.25 and *P* < .05 were considered statistical significance.

## 3 Results

### 3.1 Data collection and collation

Through careful screening, we obtained 85 OS samples with complete clinical data. Secondly, 34 genes were identified as immune-related genes in CCLs and CCRs. Details of these genes including ID, name, synonyms, chromosome, and category (see Supplementary Table S1, which illustrates the details of immune-related genes in CCLs and CCRs).

### 3.2 Bioinformatics analysis of immune-related genes in CCLs and CCRs

**Figure 1:**
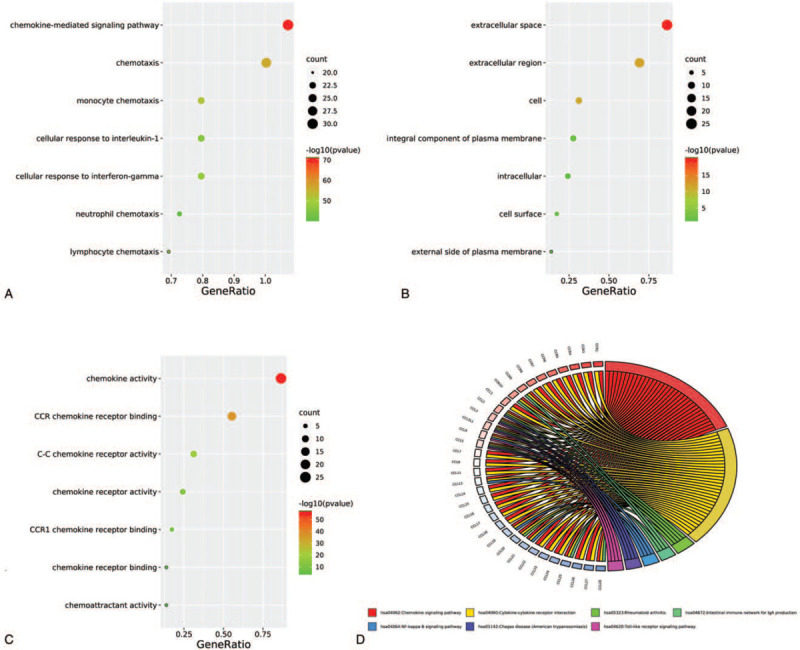
Enrichment analysis of gene ontology term and KEGG pathway of immune-related genes in C-C motif chemokine ligands and C-C motif chemokine receptors. A: biological process. B: cellular component. C: molecular function. D: KEGG pathway. KEGG = Kyoto Encyclopedia of Genes and Genomes.

**Figure 2:**
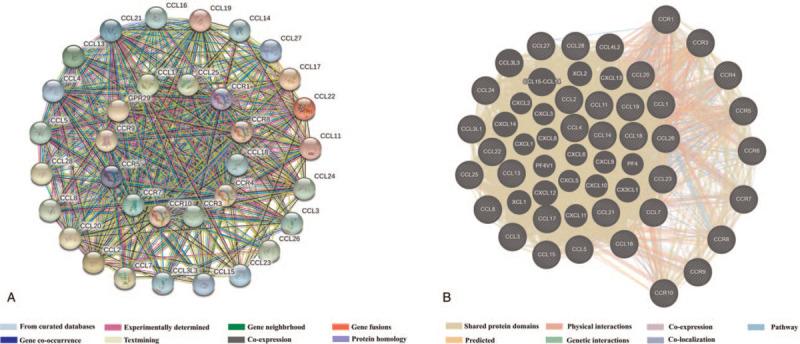
Gene interactions of immune-related genes in C-C motif chemokine ligands and C-C motif chemokine receptors. A: Search Tool for the Retrieval of Interacting Gene protein-protein association networks. B: GeneMANIA protein-protein association networks.

In Table 1 and Figure 1, the results of gene ontology and Kyoto Encyclopedia of Genes and Genomes pathway enrichment showed that these genes were enriched in cellular response to tumor necrosis factor, immune response, inflammatory response, positive regulation of T cell migration, mitogen-activated protein kinase (MAPK) cascade, positive regulation of I-kappaB kinase (IKK)/ nuclear factor-kappaB (NF-κB) signaling, Toll-like receptor (TLR) signaling pathway and NF-κB signaling pathway (see Supplementary Fig. S1, which shows the map of TLR signaling pathway and see Supplementary Fig. S2, which shows the map of NF-κB signaling pathway). In Figure 2, both STRING and GeneMANIA results showed that these immune-related genes in CCLs and CCRs exhibit close associations, such as share protein domains, experimentally determined, co-expressed and gene fusions, etc.

**Table 1:** Enrichment analysis of GO term and KEGG pathway of immune-related genes in CCLs and CCRs.

Category	Term	Count	%	*P*-value
GOTERM_BP_DIRECT	GO:0070098∼chemokine-mediated signaling pathway	31	1.071923	<.001
GOTERM_BP_DIRECT	GO:0006935∼chemotaxis	29	1.002766	<.001
GOTERM_BP_DIRECT	GO:0002548∼monocyte chemotaxis	23	0.795297	<.001
GOTERM_BP_DIRECT	GO:0071346∼cellular response to interferon-gamma	23	0.795297	<.001
GOTERM_BP_DIRECT	GO:0048247∼lymphocyte chemotaxis	20	0.691563	<.001
GOTERM_BP_DIRECT	GO:0071347∼cellular response to interleukin-1	23	0.795297	<.001
GOTERM_BP_DIRECT	GO:0030593∼neutrophil chemotaxis	21	0.726141	<.001
GOTERM_BP_DIRECT	GO:0071356∼cellular response to tumor necrosis factor	23	0.795297	<.001
GOTERM_BP_DIRECT	GO:0070374∼positive regulation of ERK1 and ERK2 cascade	25	0.864454	<.001
GOTERM_BP_DIRECT	GO:0006955∼immune response	29	1.002766	<.001
GOTERM_CC_DIRECT	GO:0005615∼extracellular space	25	0.864454	<.001
GOTERM_CC_DIRECT	GO:0005576∼extracellular region	20	0.691563	<.001
GOTERM_CC_DIRECT	GO:0005623∼cell	9	0.311203	<.001
GOTERM_CC_DIRECT	GO:0009897∼external side of plasma membrane	4	0.138313	.00663450
GOTERM_CC_DIRECT	GO:0005887∼integral component of plasma membrane	8	0.276625	.01194901
GOTERM_CC_DIRECT	GO:0009986∼cell surface	5	0.172891	.01599252
GOTERM_CC_DIRECT	GO:0005622∼intracellular	7	0.242047	.03039116
GOTERM_MF_DIRECT	GO:0008009∼chemokine activity	25	0.864454	<.001
GOTERM_MF_DIRECT	GO:0048020∼CCR chemokine receptor binding	16	0.553250	<.001
GOTERM_MF_DIRECT	GO:0016493∼C-C chemokine receptor activity	9	0.311203	<.001
GOTERM_MF_DIRECT	GO:0004950∼chemokine receptor activity	7	0.242047	<.001
GOTERM_MF_DIRECT	GO:0031726∼CCR1 chemokine receptor binding	5	0.172891	<.001
GOTERM_MF_DIRECT	GO:0042379∼chemokine receptor binding	4	0.138313	<.001
GOTERM_MF_DIRECT	GO:0042056∼chemoattractant activity	4	0.138313	<.001
GOTERM_MF_DIRECT	GO:0019957∼C-C chemokine binding	3	0.103734	<.001
GOTERM_MF_DIRECT	GO:0016004∼phospholipase activator activity	3	0.103734	<.001
GOTERM_MF_DIRECT	GO:0031730∼CCR5 chemokine receptor binding	3	0.103734	<.001
KEGG_PATHWAY	hsa04062:Chemokine signaling pathway	34	1.175657	<.001
KEGG_PATHWAY	hsa04060:Cytokine-cytokine receptor interaction	34	1.175657	<.001
KEGG_PATHWAY	hsa05323:Rheumatoid arthritis	5	0.172891	<.001
KEGG_PATHWAY	hsa04672:Intestinal immune network for IgA production	4	0.138313	.00141276
KEGG_PATHWAY	hsa04064:NF-kappa B signaling pathway	4	0.138313	.00810775
KEGG_PATHWAY	hsa05142:Chagas disease (American trypanosomiasis)	4	0.138313	.01318232
KEGG_PATHWAY	hsa04620:Toll-like receptor signaling pathway	4	0.138313	.01387510

### 3.3 Survival and correlation analysis

**Figure 3:**
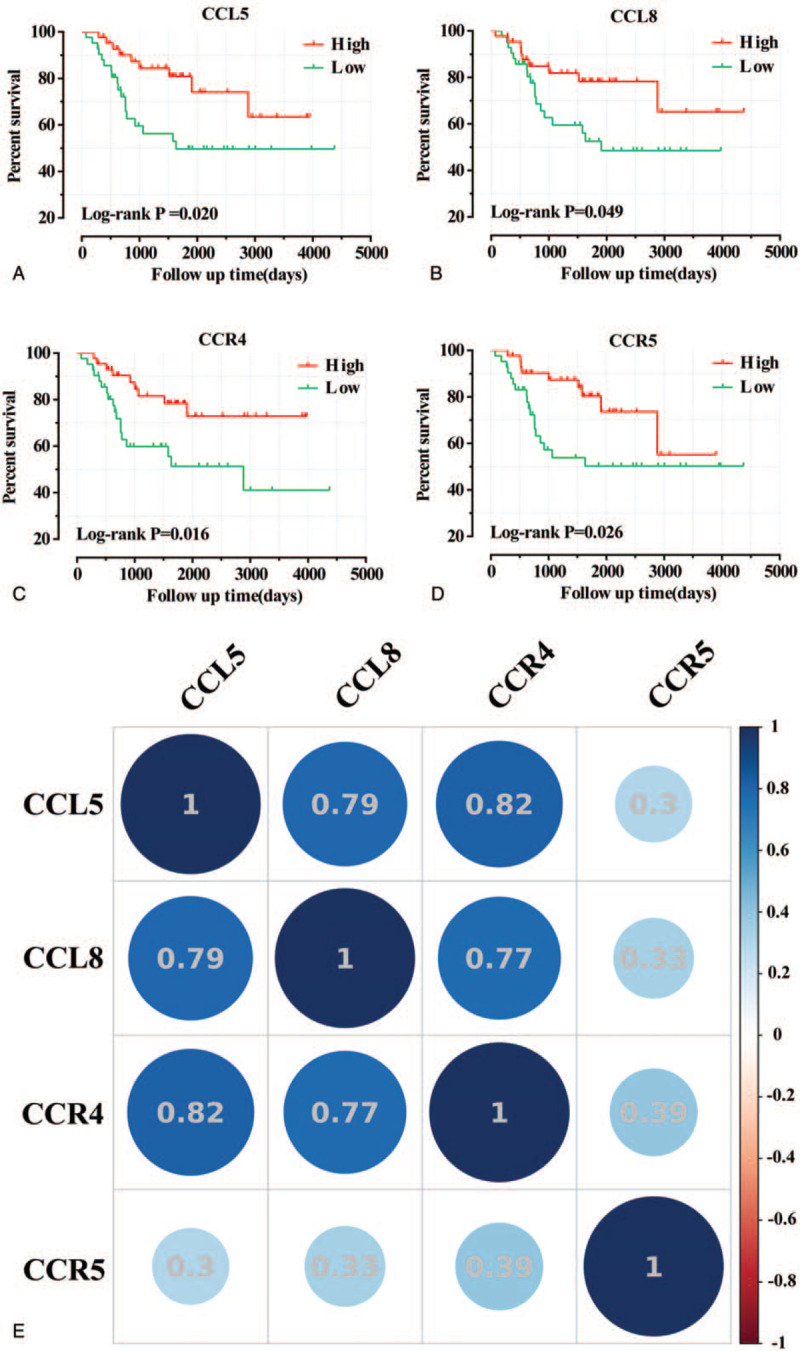
Survival analysis of immune-related genes in CCLs and CCRs and correlation analysis of significant genes. A: CCL5; B: CCL8; C: CCR4; D: CCR5. E: Pearson's correlation analysis of CCL5, CCL8, CCR4, and CCR5. The size, number, and color of the circles indicate the degree of correlation. CCL = C-C motif chemokine ligand, CCR = C-C motif chemokine receptor.

**Figure 4:**
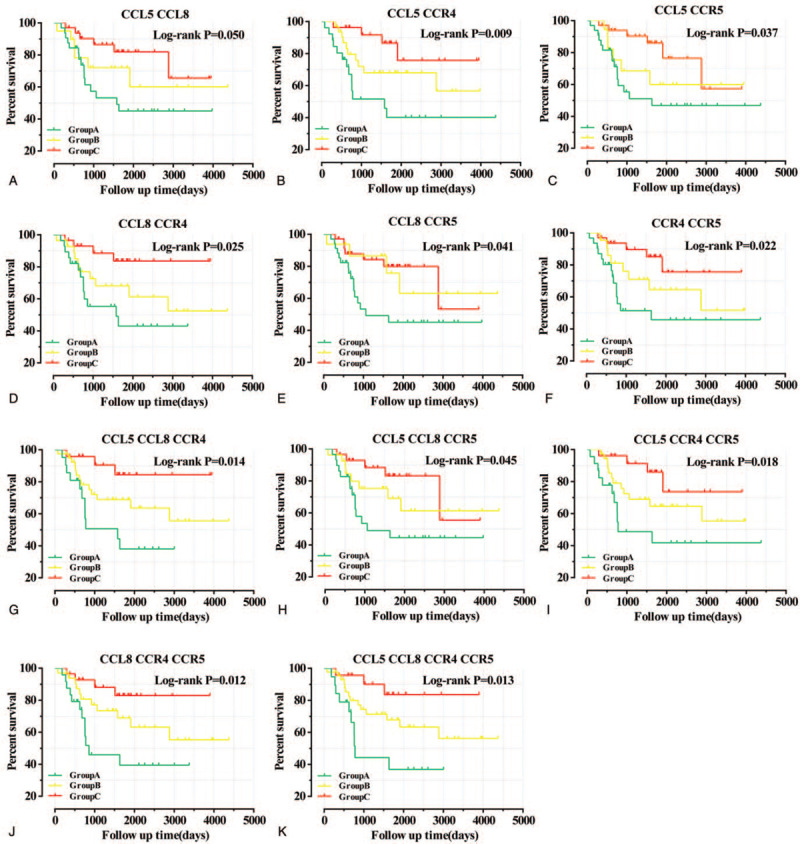
Combined effect survival analysis of CCL5, CCL8, CCR4, and CCR5 in childhood OS. A: Combined effect survival analysis of CCL5 and CCL8. B: Combined effect survival analysis of CCL5 and CCR4. C: Combined effect survival analysis of CCL5 and CCR5. D: Combined effect survival analysis of CCL8 and CCR4. E: Combined effect survival analysis of CCL8 and CCR5. F: Combined effect survival analysis of CCR4 and CCR5. G: Combined effect survival analysis of CCL5, CCL8, and CCR4. H: Combined effect survival analysis of CCL5, CCL8, and CCR5. I: Combined effect survival analysis of CCL5, CCR4, and CCR5. J: Combined effect survival analysis of CCL8, CCR4, and CCR5. K: Combined effect survival analysis of CCL5, CCL8, CCR4, and CCR5. Group A (corresponding genes were at low expression level), Group C (corresponding genes were at high expression level), Group B (excluding group A and group C, ie, the expression levels of each gene were not identical). CCL = C-C motif chemokine ligand, CCR = C-C motif chemokine receptor, OS = osteosarcoma.

**Figure 5:**
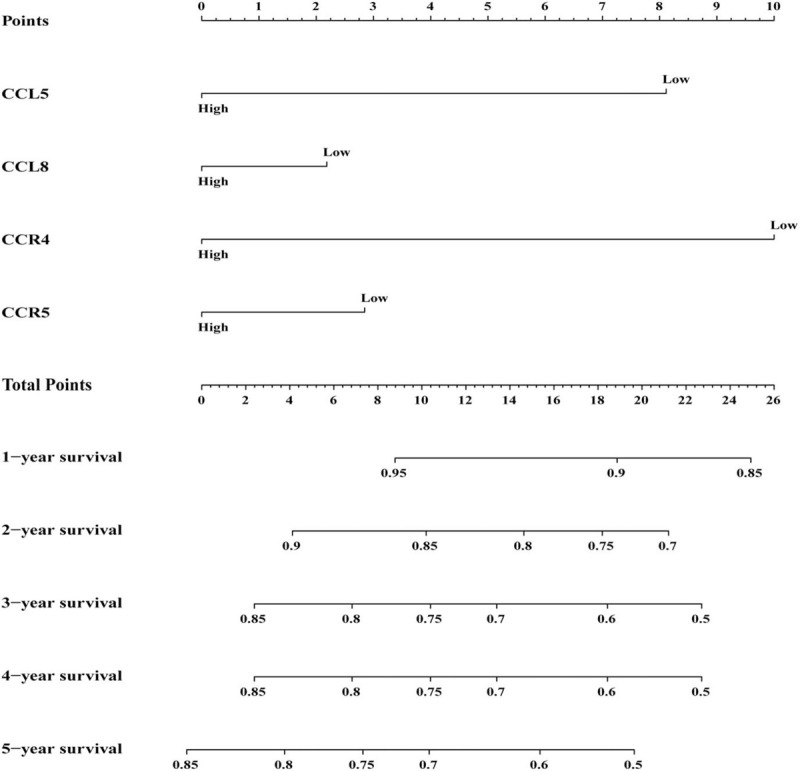
Nomogram constructed based on the expression levels of CCL5, CCL8, CCR4, and CCR5 to predict 1-, 2-, 3-, 4- and 5-year events (mortalities) CCL = C-C motif chemokine ligand, CCR = C-C motif chemokine receptor.

Survival analysis showed that CCL5, CCL8, CCR4, and CCR5 were assigned significant statistical significance. High expression of CCL5(Log-rank *P* = .020,), CCL8 (Log-rank *P* = .049,), CCR4 (Log-rank *P* = .016,) and CCR5(Log-rank *P* = .026,) have better prognosis than low expression. Based on the above 4 genes with significant statistical significance (Table 2 and Fig. 3A–D), Pearson correlation analysis shows that CCL5, CCL8, and CCR4 have a high correlation, while CCR5 has a relatively low correlation with the other 3 genes. The size, number, and color of the circles all represent the correlation between individual genes (Fig. 3E). The combined effect survival analysis of significant genes showed that group C (corresponding genes were at high expression level) had a better prognosis than group A (corresponding genes were at low expression level) and group B (excluding group A and group C, ie, the expression levels of each gene were not identical). All results are shown in Figure 4. In addition, the results of nomogram also support CCL5, CCL8, CCR4, and CCR5, which were beneficial to the prognosis of childhood OS. The high expression of all genes was at a relatively low point (Fig. 5).

**Table 2:** Survival analysis of significant genes in childhood OS patients.

Gene expression level	Patients (n = 85)	Number of events	Median survival time (days)	Crude HR (95% CI)	Crude Log-rank *P*-value
CCL5					
Low	42	18	767	1	
High	43	9	1575	0.399 (0.179–0.889)	.020
CCL8					
Low	42	18	922	1	
High	43	9	1575	0.455 (0.204–1.015)	.049
CCR4					
Low	42	18	816.5	1	
High	43	9	1625	0.386 (0.173–0.860)	.016
CCR5					
Low	42	18	816.5	1	
High	43	9	1579	0.411 (0.184–0.920)	.026

### 3.4 Construction and validation of prognostic signature based on significant genes

**Figure 6:**
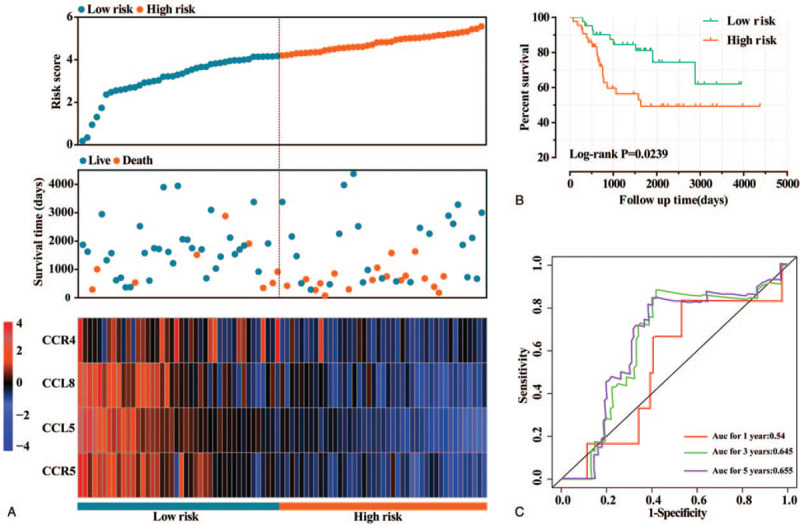
Construction and validation of prognostic signature based on the expression levels of CCL5, CCL8, CCR4, and CCR5.A: From top to bottom; risk score plot, survival status scatter plot, and heat map of the expression levels of CCL5, CCL8, CCR4, and CCR5 in high- and low-risk groups. B. Kaplan-Meier curve of high- and low-risk groups. C: The time-dependent ROC curve to validate the ability of the constructed prognostic signature model to predict 1-, 3-, and 5-year survival. CCL = C-C motif chemokine ligand, CCR = C-C motif chemokine receptor, ROC = receiver operating characteristic.

In order to more intuitively show the relationship between the expression of significant genes and prognostic risk, and to evaluate the predictive effect, CCL5, CCL8, CCR4, and CCR5 were selected to construct prognostic signatures. The regression coefficients of the four genes in the multivariate COX proportional hazards regression model were CCL5 (−0.579), CCL8 (−0.156), CCR4 (−0.714), and CCR5 (−0.203), respectively. In the current study, the specific formula used to calculate the risk score is as follows: expression of CCL5 × −0.579 + expression of CCL8 × −0.156 + expression of CCR4 × −0.714 + expression of CCR5 × −0.203 + 6 [constant]. Since the regression coefficients are all negative, in order to make the results more intuitive, the constant is used to add to the end of the risk score formula. The results of Kaplan-Meier curves of high- and low-risk groups showed that the high-risk group was significantly associated with poor prognosis of childhood OS, and the difference was statistically significant (Log-rank *P* = .024, Fig. 6B). The of the time-dependent ROC curve was 0.54, 0.645, and 0.655 for 1-year, 3-year, and 5-year survival, respectively (Fig. 6C).

### 3.5 Identified potential relationships between significant genes expression, risk score, and tumor-infiltrating immune cells

**Figure 7:**
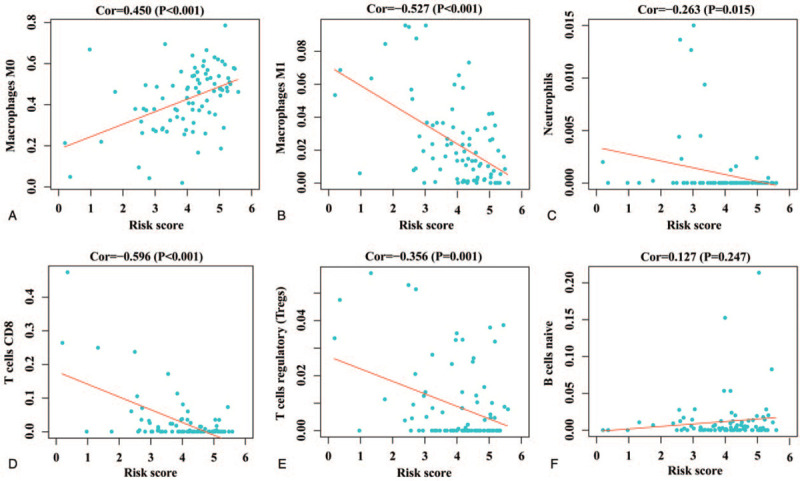
The relationships between risk score and tumor-infiltrating immune cells. A: macrophages M0; B: macrophages M1; C: neutrophils; D:CD8+ T cells; E: Tregs; F: naive B cells. Tregs, T regulatory cells.

The results of correlation analysis between the expression of significant genes and tumor-infiltrating immune cells showed that the expression of CCL5, CCL8, CCR4, and CCR5 were all correlated with the level of tumor immune cell infiltration. The expression of CCL5 was significantly correlated with the infiltration levels of macrophages M0, macrophages M1, CD8^+^ T cells and T cells regulatory (Tregs) (see Supplementary Fig. S3, which illustrates the relationships between the expression of CCL5 and tumor-infiltrating immune cells). The expression of CCL8 was significantly correlated with the infiltration levels of macrophages M0, macrophages M1, macrophages M2, neutrophils, CD8^+^ T cells, and Tregs (see Supplementary Fig. S4, which illustrates the relationships between the expression of CCL8 and tumor-infiltrating immune cells). The expression of CCR4 was significantly correlated with the infiltration levels of eosinophils, macrophages M0, and CD8^+^ T cells (see Supplementary Fig. S5, which illustrates the relationships between the expression of CCR4 and tumor-infiltrating immune cells). The expression of CCR5 was significantly correlated with the infiltration levels of macrophages M0, macrophages M1, macrophages M2, neutrophils, resting natural killer (NK) cells, CD8^+^ T cells, and Tregs (see Supplementary Fig. S6, which illustrates the relationships between the expression of CCR5 and tumor-infiltrating immune cells). After successfully constructing the prognostic signature model, we analyzed the correlation between risk score and OS immune infiltration level. The results showed that the risk score was positively correlated with the expression of macrophages M0 (*P* < .001). In addition, the risk score was negatively correlated with the expression of macrophages M1 (*P* < .001), neutrophils (*P* = .015), CD8^+^ T cells (*P* < .001), and Tregs (*P* = .001). The results were shown in Figure 7.

### 3.6 GSEA

**Figure 8:**
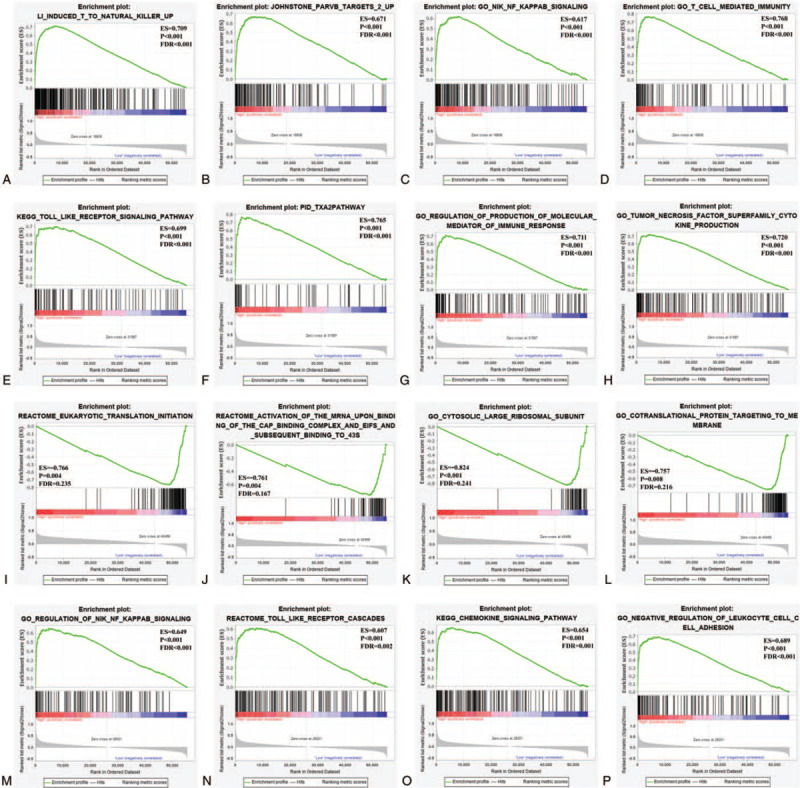
The results of GSEA for CCL5 (A–D), CCL8 (E–H), CCR4 (I–L), and CCR5 (M–P) in childhood OS. CCL = C-C motif chemokine ligand, CCR = C-C motif chemokine receptor, GSEA = gene set enrichment analysis, OS = osteosarcoma.

The analysis of c2 and c5 gene sets showed that CCL5 was mainly enriched in TLR signaling pathway, T to NK cells, Parvin-beta, MAPK family signaling cascade, T cell immune response, and NF-κB pathway (Fig. 8A–D and see Supplementary Table S2, which shows part of the results of GSEA analysis in the high expression group of CCL5). CCL8 was rich in MAPK family signaling cascade, T to NK cells, NF-κB pathway, TLR pathway, production of tumor necrosis factor superfamily cytokines in c2 and c5 gene sets (Fig. 8E–H and see Supplementary Table S3, which shows part of the results of GSEA analysis in the high expression group of CCL8).CCR4 was enriched in eukaryotic translation initiation, eukaryotic translation elongation, ribosome and co-translation protein of target membrane in c2 and c5 gene sets (Fig. 8I–L and see Supplementary Table S4, which shows part of the results of GSEA analysis in the low expression group of CCR4). The results of c2 and c5 gene sets showed that CCR5 was rich in TLR signaling pathway, MAPK family signaling cascade, NIK/NF-κB signaling, T to NK cells and chemokine signaling pathway (Fig. 8M–P and see Supplementary Table S5, which shows part of the results of GSEA analysis in the high expression group of CCR5).

## 4 Discussion

Although immunotherapy has made tremendous progress in adult tumors, less success has been achieved in pediatric tumors. This may be associated with a significant reduction in mutational load in childhood cancers leading to a reduction in the number of neoantigens in immunotherapy. However, the relative low toxicity of immunotherapy makes the immunotherapy of pediatric cancer exhibit infinite charm.^[14,29]^ Fortunately, bone biology is inextricably linked to the immune system, which has led to significant implications for immunotherapy of childhood OS.^[30]^ Therefore, many researchers have carried out a large number of studies on OS immunotherapy, such as denosumab targeting RANK ligand,^[31]^ adding decitabine to upregulate the expression of cancer antigens,^[32]^ T-VEC and anti-PD-1 antibody pembrolizumab combination test,^[33,34]^ etc. However, in the past 3 decades, progress in improving the prognosis of OS patients has been limited by the failure to identify new active agents or to optimize the use of existing drugs.^[32]^ Therefore, it is necessary to identify potential immune prognostic targets for childhood OS. The purpose of the present study was to identify the relationship between immune-related genes in CCLs, CCRs, and childhood OS patients’ prognosis.

Survival analysis of immune-related genes in CCLs and CCRs showed that high expression of CCL5, CCL8, CCR4, and CCR5 all represented a good prognostic signal for childhood OS. The biological function of CCL5 in tumor is not clear. On the one hand, the production of CCL5 is related to the induction of appropriate immune response to tumor, but on the other hand, CCL5 was related to the progression and metastasis of tumor.^[35,36]^ It was reported that the activation of CCL5 will raise tumor-associated macrophages into tumor microenvironment. Tumor-associated macrophages interact with tumor cells to secrete multiple factors and promote the release of matrix metalloproteinase, thus activating NF-κB signaling pathway to induce epithelial-mesenchymal transition.^[37]^ In addition, CCL5 blocking the selective depletion of Treg cells in tumor microenvironment can promote the anti-tumor immune response of pancreatic ductal adenocarcinoma patients.^[38]^ But we also note that in esophageal squamous cell carcinoma, CCL5 induces CD8 + T cells to infiltrate tumor cells and is associated with better survival.^[39]^ We also found that CCL8 expression leads to the migration of monocytes and T lymphocytes and plays an anti-tumor role. And CCL8 can directly inhibit the proliferation of tumor cells or transplant tumor cells into tissues.^[40]^ Di Stasi et al found that T lymphocytes coexpressing CCR4 and targeting CD30 chimeric antigen receptor enhance homing and antitumor activity in Hodgkin's tumor model.^[41]^ In addition, super-agonists of CCR5 regulate upstream and downstream events of antitumor responses by participating in adaptive and innate immunity.^[20,42]^ These findings all effectively corroborate our current findings on CCL5, CCL8, CCR4, and CCR5, however, the current findings have limitations because they were obtained from a single cohort and therefore require an external validation cohort to prove the current results.

Tumor-infiltrating immune cells are important for studying the interaction between tumor and immunity, so we explored the correlation between risk score and immune cell infiltration. The results showed that patients with high-risk scores had high levels of macrophages Mo infiltration, whereas patients with low-risk scores had high levels of macrophages M1, neutrophils, CD8^+^ T cells, and Tregs infiltration. Similar to our results, Yang et al found higher levels of macrophages M0 are a high-risk immune subtype for cancer recurrence in the digestive system.^[43]^ Xiong et al found macrophages M1 has antitumor effect in colorectal cancer.^[44]^ Granot et al found that tumor-entrained neutrophils inhibited lung dissemination before transplantation.^[45]^ T cells are an important part of immune cells, which have antitumor effect, especially CD8^+^ and CD4^+^ T cells.^[46]^ Previous studies have found that the presence of Tregs is associated with a positive prognosis in head-and-neck and gastric cancer.^[47,48]^ Interestingly, some subtypes of tumor-infiltrating immune cells (such as macrophages M2, B cells, and CD4 ^+^ T cells) infiltrate in different tumors, and the difference is given statistical significance, but we did not find any association in the current study, even the subtype infiltration results and our current study results have the opposite situation, which may be related to the difference between different tumors and the functional heterogeneity of immune cell subtypes in tumor progression.

Comprehensive analysis of functional annotations and GSEA results showed that CCL5, CCL8, and CCR5 were significantly enriched in immunity, inflammation, TLR signaling pathway, MAPK family signaling cascade, and NF-κB pathway. The inseparable relationship between immunity, inflammation, and cancer has been widely reported.^[49,50]^ Previous studies have shown that TLRs are negative regulators of cancer and activation of TLRs can lead to activation of MAPKs and NF-κB.^[51]^ However, it has also been found that some TLRs are found in many tumors and produce the effect of tumor cell proliferation and resistance to chemotherapy, but depend on the TLR and tumor type.^[52]^ The role of the NF-κB pathway in cancer is a double-edged sword. On the one hand, the activation of NF-kappa B can cause immune defense and target transformed cells; on the other hand, NF-κB is constitutively activated in many types of cancer and can play a variety of cancer-promoting roles.^[53]^ Schulze-Osthoff et al found that complex crosstalk effects occur between the NF-κB pathway and members of the MAPK family.^[54]^ In addition, the results of GSEA showed that CCR4 with low expression was enriched at the initiation and elongation of eukaryotic translation. These pathways are also closely related to cancer.^[55,56]^ We speculate that CCL5, CCL8, and CCR5 may inhibit the progression of childhood OS through the complex regulation of TLR signaling pathway, MAPK family signaling cascade, and NF-κB pathway. And CCR4 may influence the prognosis of childhood OS by regulating eukaryotic translation. However, these findings need further experimental verification.

However, there are some limitations at present. First, our analysis sample size is relatively small, which may lead to false-negative results. Secondly, the current study is retrospective and needs further prospective study. Finally, the data of the current study come from a single cohort, and the combination of data from multiple cohorts will make the results more convincing.

Although the current study has some limitations, our study provides a new idea for immunotherapy of childhood OS. We identified immune-related genes in CCLs and CCRs that influence childhood OS prognosis, and secondly, we identified potential molecular mechanisms that influence childhood OS prognosis through functional annotation and GSEA. The selected genes and molecular mechanisms can be prioritized for experimental studies to demonstrate their clinical value.

## 5 Conclusion

CCL5, CCL8, CCR4, and CCR5 are potential biomarkers for the prognosis of childhood OS and have great clinical prospects. In addition, CCL5, CCL8, and CCR5 may affect the prognosis of childhood OS through complex regulation among TLR signaling pathway, MAPK family signaling cascade, and NF-κB pathway, while CCR4 may affect the prognosis of childhood OS by affecting eukaryotic translation.

## Acknowledgments

We are grateful to Dr Xinli Zhan (Spine and Osteopathy Ward, The First Affiliated Hospital of Guangxi Medical University,) for his kindly assistance in all stages of the present study.

## Author contributions

**Conceptualization:** Zide Zhang, Tuo Liang, Chaojie Yu, Zhaojie Qin.

**Data curation:** Zide Zhang, Xin Zhou, Jiang Xue, Haopeng Zeng.

**Formal analysis:** Zide Zhang, Chong Liu, Tuo Liang.

**Funding acquisition:** Zide Zhang.

**Investigation:** Zide Zhang, Zhaojun Lu, Guoyong Xu.

**Methodology:** Zide Zhang, Chong Liu, Xin Zhou.

**Project administration:** Zide Zhang, Chong Liu, Zequn Wang.

**Resources:** Zide Zhang.

**Software:** Zide Zhang, Xinli Zhan.

**Supervision:** Zide Zhang.

**Validation:** Zide Zhang, Jiarui Chen, Jie Jiang.

**Visualization:** Zide Zhang, Chong Liu.

**Writing – original draft:** Zide Zhang.

**Writing – review & editing:** Zide Zhang, Xinli Zhan.

## Supplementary Material

Supplemental Digital Content

## Supplementary Material

Supplemental Digital Content

## Supplementary Material

Supplemental Digital Content

## Supplementary Material

Supplemental Digital Content

## Supplementary Material

Supplemental Digital Content

## Supplementary Material

Supplemental Digital Content

## Supplementary Material

Supplemental Digital Content

## Supplementary Material

Supplemental Digital Content

## Supplementary Material

Supplemental Digital Content

## Supplementary Material

Supplemental Digital Content

## Supplementary Material

Supplemental Digital Content

## References

[1] Hou P Ji M Yang B Quantitative analysis of promoter hypermethylation in multiple genes in osteosarcoma. Cancer 2006;106:1602–9. 16502411 10.1002/cncr.21762

[2] Anwar MA El-Baba C Elnaggar MH Novel therapeutic strategies for spinal osteosarcomas. Semin Cancer Biol 2020;64:83–92. 31152785 10.1016/j.semcancer.2019.05.018

[3] Moreno F Cacciavillano W Cipolla M Childhood osteosarcoma: Incidence and survival in Argentina. Report from the National Pediatric Cancer Registry, ROHA Network 2000-2013. Pediatr Blood Cancer 2017;64:e26533. 10.1002/pbc.26533 28409896

[4] Kansara M Teng MW Smyth MJ Translational biology of osteosarcoma. Nat Rev Cancer 2014;14:722–35. 25319867 10.1038/nrc3838

[5] Wang J Liu S Shi J The role of miRNA in the diagnosis, prognosis, and treatment of osteosarcoma. Cancer Biother Radiopharm 2019;34:605–13. 31674804 10.1089/cbr.2019.2939

[6] Ma C Nie XG Wang YL CBX3 predicts an unfavorable prognosis and promotes tumorigenesis in osteosarcoma. Mol Med Rep 2019;19:4205–12. 30942427 10.3892/mmr.2019.10104 PMC6470990

[7] Moore DD Luu HH Osteosarcoma. Cancer Treat Res 2014;162:65–92. 25070231 10.1007/978-3-319-07323-1_4

[8] Wu S Gu Z Wu Y LINC00324 accelerates the proliferation and migration of osteosarcoma through regulating WDR66. J Cell Physiol 2020;235:339–48. 31225659 10.1002/jcp.28973

[9] Zhang C Zheng JH Lin ZH Profiles of immune cell infiltration and immune-related genes in the tumor microenvironment of osteosarcoma. Aging (Albany NY) 2020;12:3486–501. 32039832 10.18632/aging.102824 PMC7066877

[10] Ahmed N Brawley VS Hegde M Human epidermal growth factor receptor 2 (HER2) -specific chimeric antigen receptor-modified T cells for the immunotherapy of HER2-positive sarcoma. J Clin Oncol 2015;33:1688–96. 25800760 10.1200/JCO.2014.58.0225 PMC4429176

[11] Wang Y Yu W Zhu J Anti-CD166/4-1BB chimeric antigen receptor T cell therapy for the treatment of osteosarcoma. J Exp Clin Cancer Res 2019;38:168. 30995926 10.1186/s13046-019-1147-6 PMC6471997

[12] Couzin-Frankel J Breakthrough of the year 2013. Cancer immunotherapy. Science (New York, NY) 2013;342:1432–3. 10.1126/science.342.6165.1432 24357284

[13] Schumacher TN Schreiber RD Neoantigens in cancer immunotherapy. Science (New York, NY) 2015;348:69–74. 10.1126/science.aaa4971 25838375

[14] Wedekind MF Denton NL Chen CY Pediatric cancer immunotherapy: opportunities and challenges. Paediatr Drugs 2018;20:395–408. 29948928 10.1007/s40272-018-0297-x PMC6153971

[15] Wang T Wei Y Tian L C-C motif chemokine ligand 5 (CCL5) levels in gastric cancer patient sera predict occult peritoneal metastasis and a poorer prognosis. Int J Surg (Lond, Engl) 2016;32:136–42. 10.1016/j.ijsu.2016.07.008 27398691

[16] Szczepanik AM Siedlar M Szura M Preoperative serum chemokine (C-C motif) ligand 2 levels and prognosis in colorectal cancer. Pol Arch Med Wewn 2015;125:443–51. 26020569 10.20452/pamw.2886

[17] Gao J Li ZH Tang W Chemokine C-C motif ligand 18 expression correlates with tumor malignancy in breast cancer. Pathologie-biologie 2015;63:199–203. 26294068 10.1016/j.patbio.2015.07.001

[18] Lien MY Tsai HC Chang AC Chemokine CCL4 induces vascular endothelial growth factor C expression and lymphangiogenesis by miR-195-3p in oral squamous cell carcinoma. Front Immunol 2018;9:412. 29599774 10.3389/fimmu.2018.00412 PMC5863517

[19] Ryu H Baek SW Moon JY C-C motif chemokine receptors in gastric cancer. Mol Clin Oncol 2018;8:3–8. 29285394 10.3892/mco.2017.1470 PMC5738695

[20] Zhou X Liao X Wang X Clinical significance and prospective molecular mechanism of C-C motif chemokine receptors in patients with early-stage pancreatic ductal adenocarcinoma after pancreaticoduodenectomy. Oncol Rep 2019;42:1856–68. 31432181 10.3892/or.2019.7277 PMC6775805

[21] Sherman BT Huang da W Tan Q DAVID Knowledgebase: a gene-centered database integrating heterogeneous gene annotation resources to facilitate high-throughput gene functional analysis. BMC Bioinformatics 2007;8:426. 17980028 10.1186/1471-2105-8-426 PMC2186358

[22] Ashburner M Ball CA Blake JA Gene ontology: tool for the unification of biology. The gene ontology consortium. Nat Genet 2000;25:25–9. 10802651 10.1038/75556 PMC3037419

[23] Kanehisa M Furumichi M Tanabe M KEGG: new perspectives on genomes, pathways, diseases and drugs. Nucleic Acids Res 2017;45:D353–61. 27899662 10.1093/nar/gkw1092 PMC5210567

[24] Szklarczyk D Gable AL Lyon D STRING v11: protein-protein association networks with increased coverage, supporting functional discovery in genome-wide experimental datasets. Nucleic Acids Res 2019;47:D607–13. 30476243 10.1093/nar/gky1131 PMC6323986

[25] Montojo J Zuberi K Rodriguez H GeneMANIA: Fast gene network construction and function prediction for Cytoscape. F1000Res 2014;3:153. 25254104 10.12688/f1000research.4572.1 PMC4168749

[26] Zhou X Liao X Wang X Noteworthy prognostic value of phospholipase C delta genes in early stage pancreatic ductal adenocarcinoma patients after pancreaticoduodenectomy and potential molecular mechanisms. Cancer Med 2020;9:859–71. 31808619 10.1002/cam4.2699 PMC6997088

[27] Subramanian A Tamayo P Mootha VK Gene set enrichment analysis: a knowledge-based approach for interpreting genome-wide expression profiles. Proc Natl Acad Sci U S A 2005;102:15545–50. 16199517 10.1073/pnas.0506580102 PMC1239896

[28] Liberzon A Birger C Thorvaldsdóttir H The Molecular Signatures Database (MSigDB) hallmark gene set collection. Cell systems 2015;1:417–25. 26771021 10.1016/j.cels.2015.12.004 PMC4707969

[29] Huang MA Krishnadas DK Lucas KG Cellular and antibody based approaches for pediatric cancer immunotherapy. J Immunol Res 2015;2015:675269. 26587548 10.1155/2015/675269 PMC4637498

[30] Kager L Tamamyan G Bielack S Novel insights and therapeutic interventions for pediatric osteosarcoma. Future Oncol 2017;13:357–68. 27651036 10.2217/fon-2016-0261

[31] de Groot AF Appelman-Dijkstra NM van der Burg SH The anti-tumor effect of RANKL inhibition in malignant solid tumors - a systematic review. Cancer Treat Rev 2018;62:18–28. 29154022 10.1016/j.ctrv.2017.10.010

[32] Wedekind MF Wagner LM Cripe TP Immunotherapy for osteosarcoma: where do we go from here? Pediatr Blood Cancer 2018;65:e27227. 29923370 10.1002/pbc.27227

[33] Geiss C Kis Z Leuchs B Preclinical testing of an oncolytic parvovirus: standard protoparvovirus H-1PV efficiently induces osteosarcoma cell lysis in vitro. Viruses 2017;9 doi: 10.3390/v9100301. 10.3390/v9100301 PMC5691652 29039746

[34] Hingorani P Sampson V Lettieri C Oncolytic viruses for potential osteosarcoma therapy. Adv Exp Med Biol 2014;804:259–83. 24924179 10.1007/978-3-319-04843-7_14

[35] Aldinucci D Colombatti A The inflammatory chemokine CCL5 and cancer progression. Mediators Inflamm 2014;2014:292376. 24523569 10.1155/2014/292376 PMC3910068

[36] Böttcher JP Bonavita E Chakravarty P NK cells stimulate recruitment of cDC1 into the tumor microenvironment promoting cancer immune control. Cell 2018;172: 1022-37.e14. 10.1016/j.cell.2018.01.004 PMC5847168 29429633

[37] An G Wu F Huang S Effects of CCL5 on the biological behavior of breast cancer and the mechanisms of its interaction with tumor associated macrophages. Oncol Rep 2019;42:2499–511. 31578575 10.3892/or.2019.7344 PMC6826325

[38] Wang X Lang M Zhao T Cancer-FOXP3 directly activated CCL5 to recruit FOXP3(+)Treg cells in pancreatic ductal adenocarcinoma. Oncogene 2017;36:3048–58. 27991933 10.1038/onc.2016.458 PMC5454319

[39] Goto M Liu M Chemokines and their receptors as biomarkers in esophageal cancer. Esophagus 2020;17:113–21. 31773415 10.1007/s10388-019-00706-8

[40] Hiwatashi K Tamiya T Hasegawa E Suppression of SOCS3 in macrophages prevents cancer metastasis by modifying macrophage phase and MCP2/CCL8 induction. Cancer Lett 2011;308:172–80. 21624767 10.1016/j.canlet.2011.04.024

[41] Di Stasi A De Angelis B Rooney CM T lymphocytes coexpressing CCR4 and a chimeric antigen receptor targeting CD30 have improved homing and antitumor activity in a Hodgkin tumor model. Blood 2009;113:6392–402. 19377047 10.1182/blood-2009-03-209650 PMC2710932

[42] Dorgham K Abadie V Iga M Engineered CCR5 superagonist chemokine as adjuvant in anti-tumor DNA vaccination. Vaccine 2008;26:3252–60. 18479788 10.1016/j.vaccine.2008.04.003

[43] Yang S Liu T Cheng Y Immune cell infiltration as a biomarker for the diagnosis and prognosis of digestive system cancer. Cancer Sci 2019;110:3639–49. 31605436 10.1111/cas.14216 PMC6890448

[44] Xiong Y Wang K Zhou H Profiles of immune infiltration in colorectal cancer and their clinical significant: A gene expression-based study. Cancer Med 2018;7:4496–508. 30117315 10.1002/cam4.1745 PMC6144159

[45] Granot Z Henke E Comen EA Tumor entrained neutrophils inhibit seeding in the premetastatic lung. Cancer Cell 2011;20:300–14. 21907922 10.1016/j.ccr.2011.08.012 PMC3172582

[46] Ge P Wang W Li L Profiles of immune cell infiltration and immune-related genes in the tumor microenvironment of colorectal cancer. Biomed Pharmacother 2019;118:109228. 31351430 10.1016/j.biopha.2019.109228

[47] Mougiakakos D Choudhury A Lladser A Regulatory T cells in cancer. Adv Cancer Res 2010;107:57–117. 20399961 10.1016/S0065-230X(10)07003-X

[48] Haas M Dimmler A Hohenberger W Stromal regulatory T-cells are associated with a favourable prognosis in gastric cancer of the cardia. BMC Gastroenterol 2009;9:65. 19732435 10.1186/1471-230X-9-65 PMC2749861

[49] Chen DS Mellman I Elements of cancer immunity and the cancer-immune set point. Nature 2017;541:321–30. 28102259 10.1038/nature21349

[50] Greten FR Grivennikov SI Inflammation and cancer: triggers, mechanisms, and consequences. Immunity 2019;51:27–41. 31315034 10.1016/j.immuni.2019.06.025 PMC6831096

[51] Rakoff-Nahoum S Medzhitov R Toll-like receptors and cancer. Nat Rev Cancer 2009;9:57–63. 19052556 10.1038/nrc2541

[52] Dajon M Iribarren K Cremer I Toll-like receptor stimulation in cancer: a pro- and anti-tumor double-edged sword. Immunobiology 2017;222:89–100. 27349597 10.1016/j.imbio.2016.06.009

[53] Hoesel B Schmid JA The complexity of NF-κB signaling in inflammation and cancer. Mol Cancer 2013;12:86. 23915189 10.1186/1476-4598-12-86 PMC3750319

[54] Schulze-Osthoff K Ferrari D Riehemann K Regulation of NF-kappa B activation by MAP kinase cascades. Immunobiology 1997;198:35–49. 9442376 10.1016/s0171-2985(97)80025-3

[55] Hassan MK Kumar D Naik M The expression profile and prognostic significance of eukaryotic translation elongation factors in different cancers. PLoS One 2018;13:e0191377. 29342219 10.1371/journal.pone.0191377 PMC5771626

[56] Ali MU Ur Rahman MS Jia Z Eukaryotic translation initiation factors and cancer. Tumour Biol 2017;39: 1010428317709805. 10.1177/1010428317709805 28653885

