# Supplementary figures and images for: Establishment of immune prognostic signature and analysis of prospective molecular mechanisms in childhood osteosarcoma patients
Source: Medicine (Baltimore). 2020 Nov 13;99(46):e23251. doi: 10.1097/MD.0000000000023251 (PMC7668544; doi:10.1097/MD.0000000000023251)

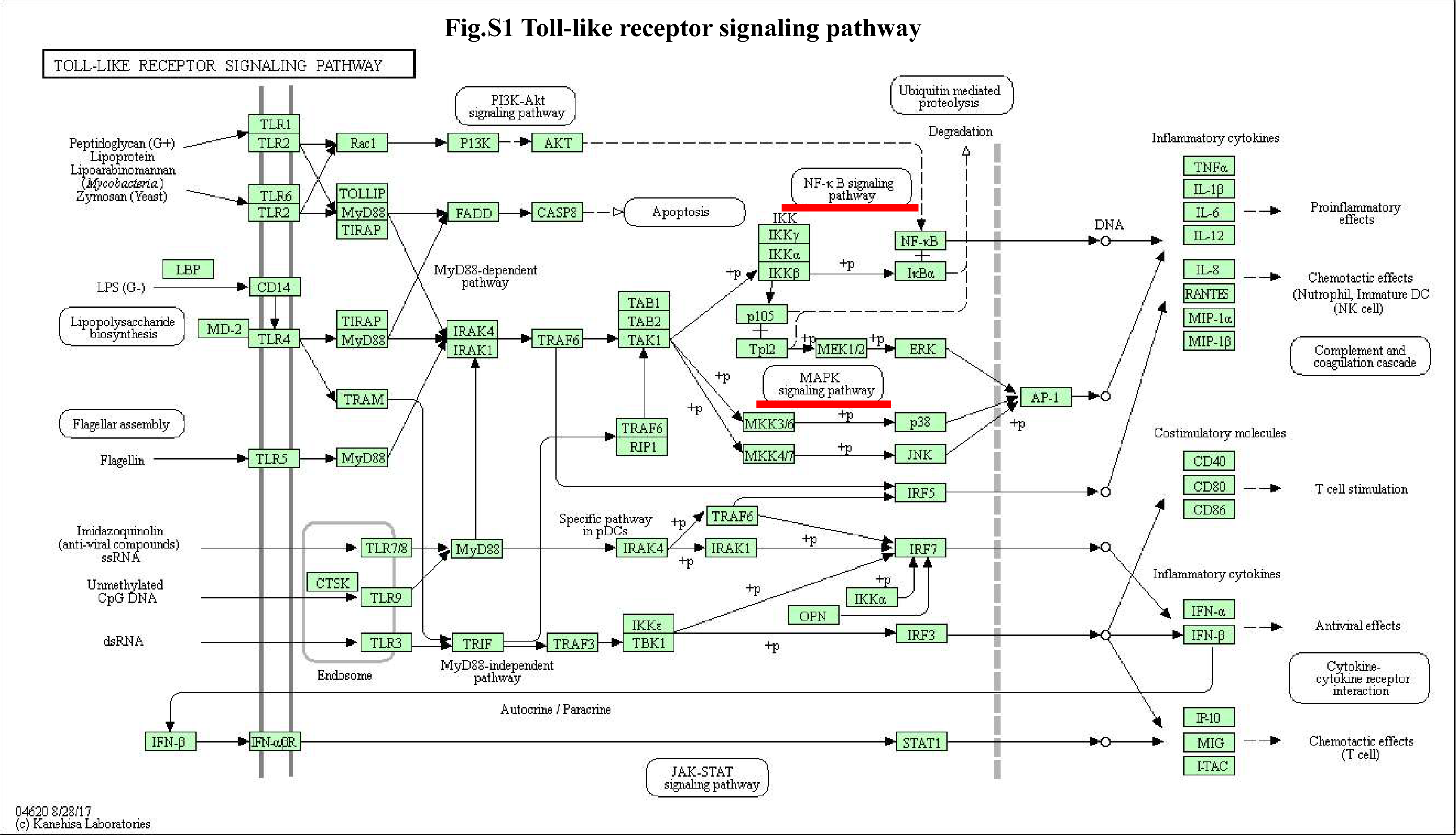

Supplement: Supplemental Digital Content [file medi-99-e23251-s001.tif]

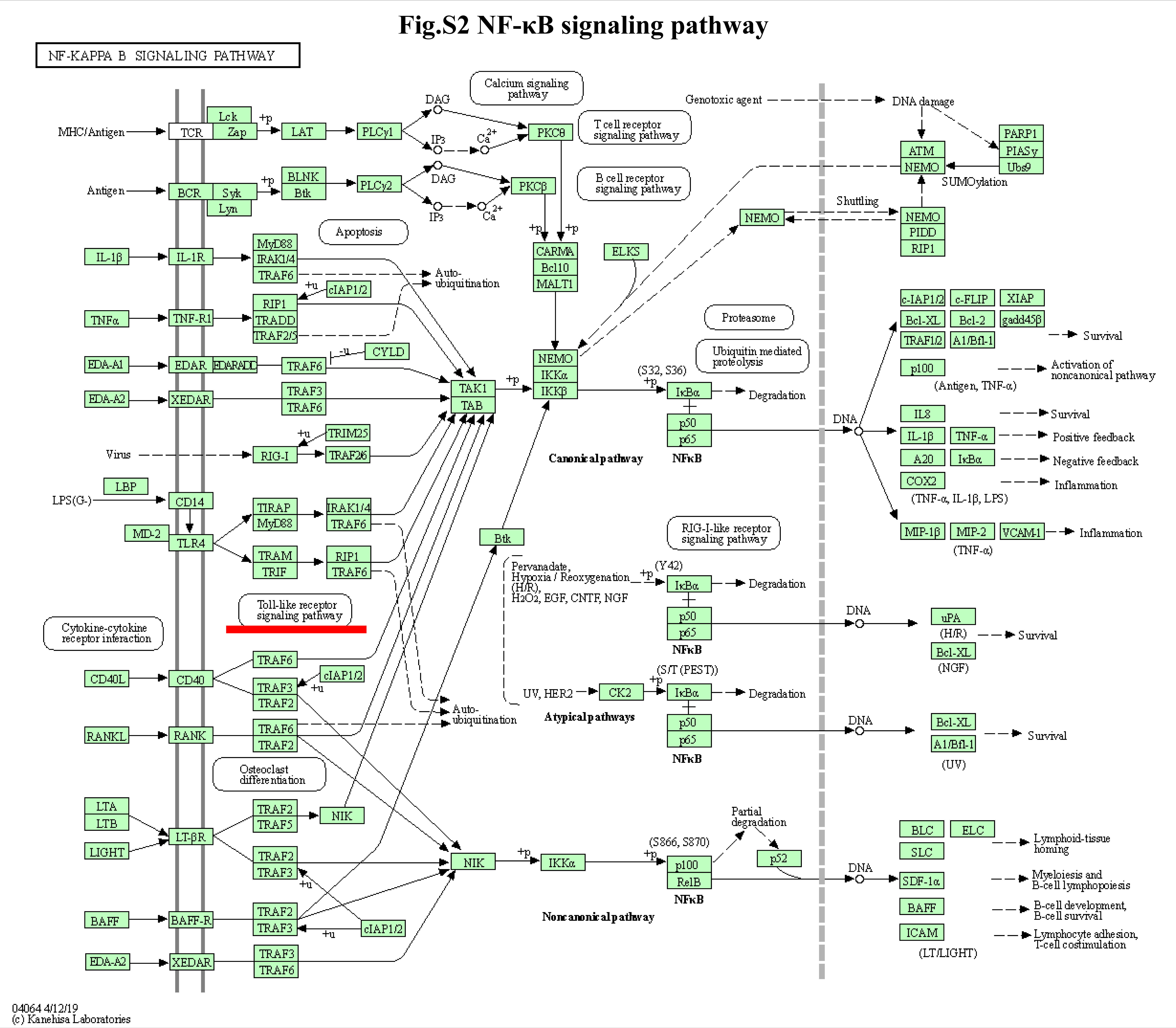

Supplement: Supplemental Digital Content [file medi-99-e23251-s002.tif]

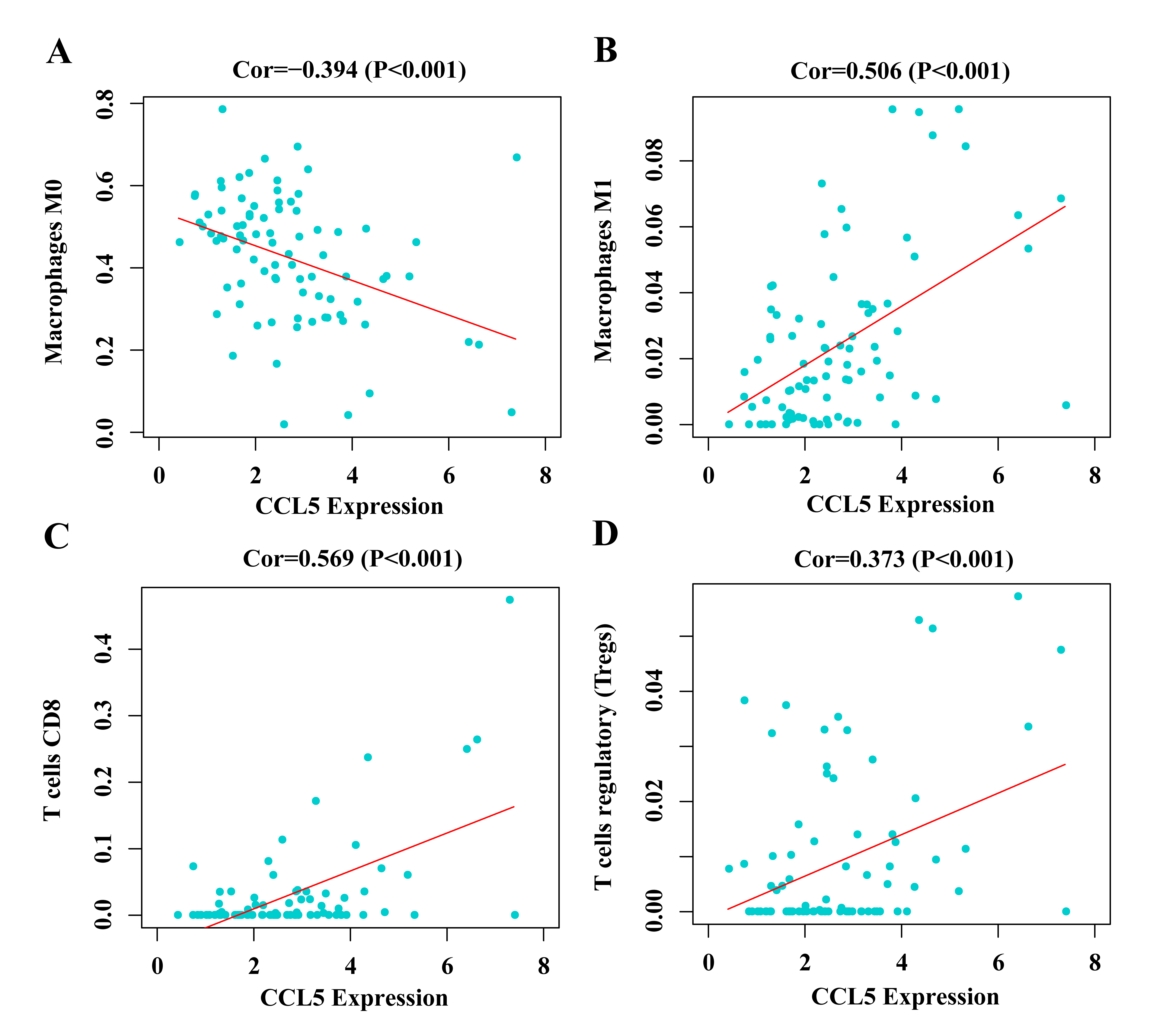

Supplement: Supplemental Digital Content [file medi-99-e23251-s003.tif]

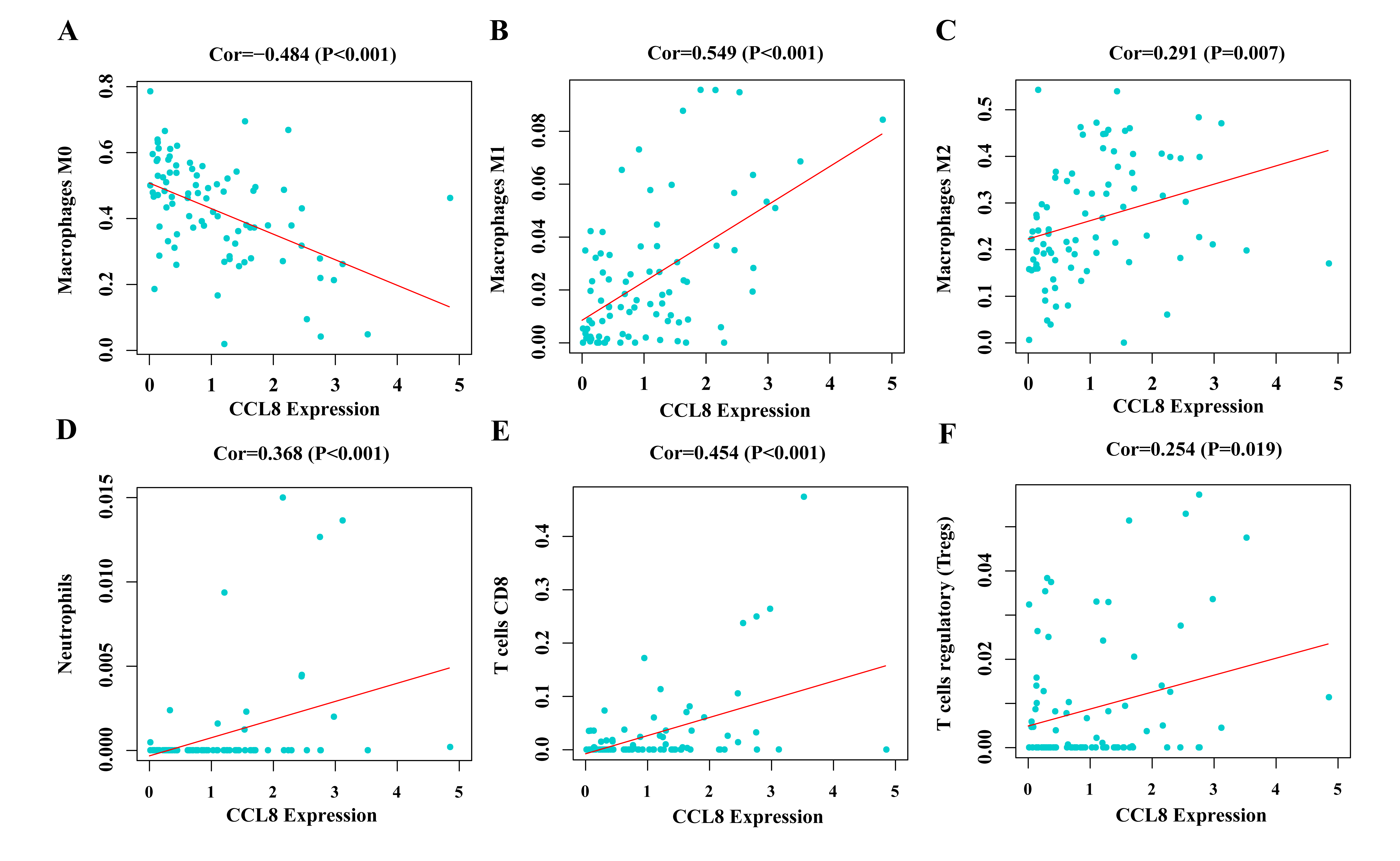

Supplement: Supplemental Digital Content [file medi-99-e23251-s004.tif]

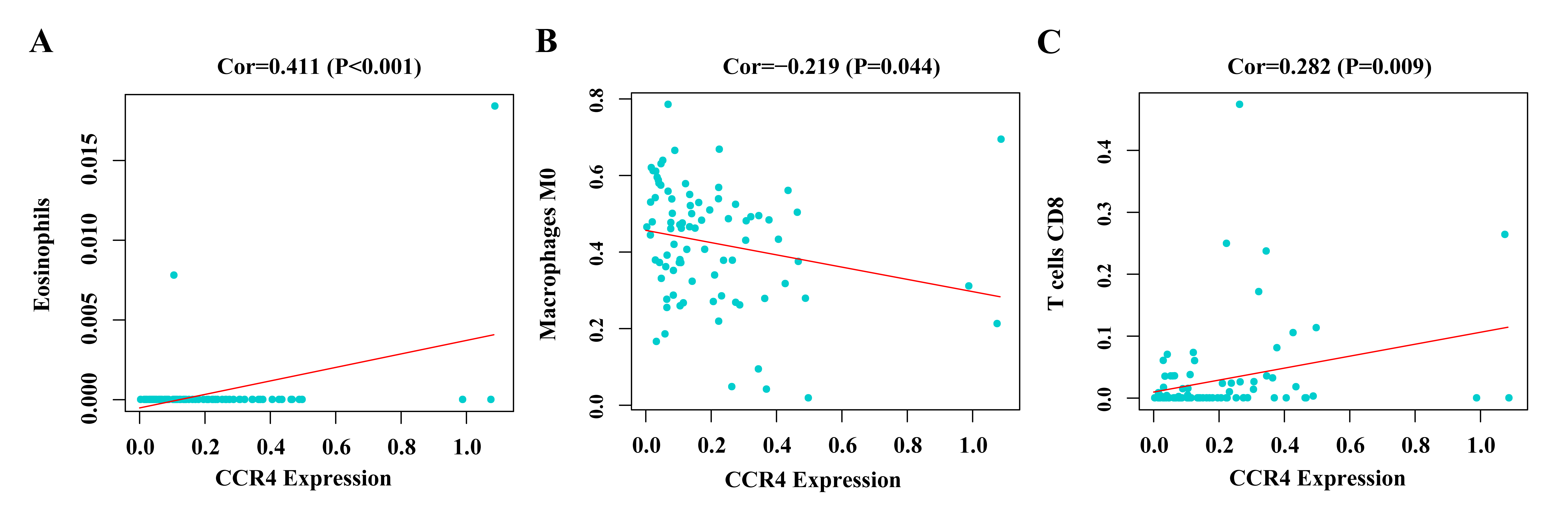

Supplement: Supplemental Digital Content [file medi-99-e23251-s005.tif]

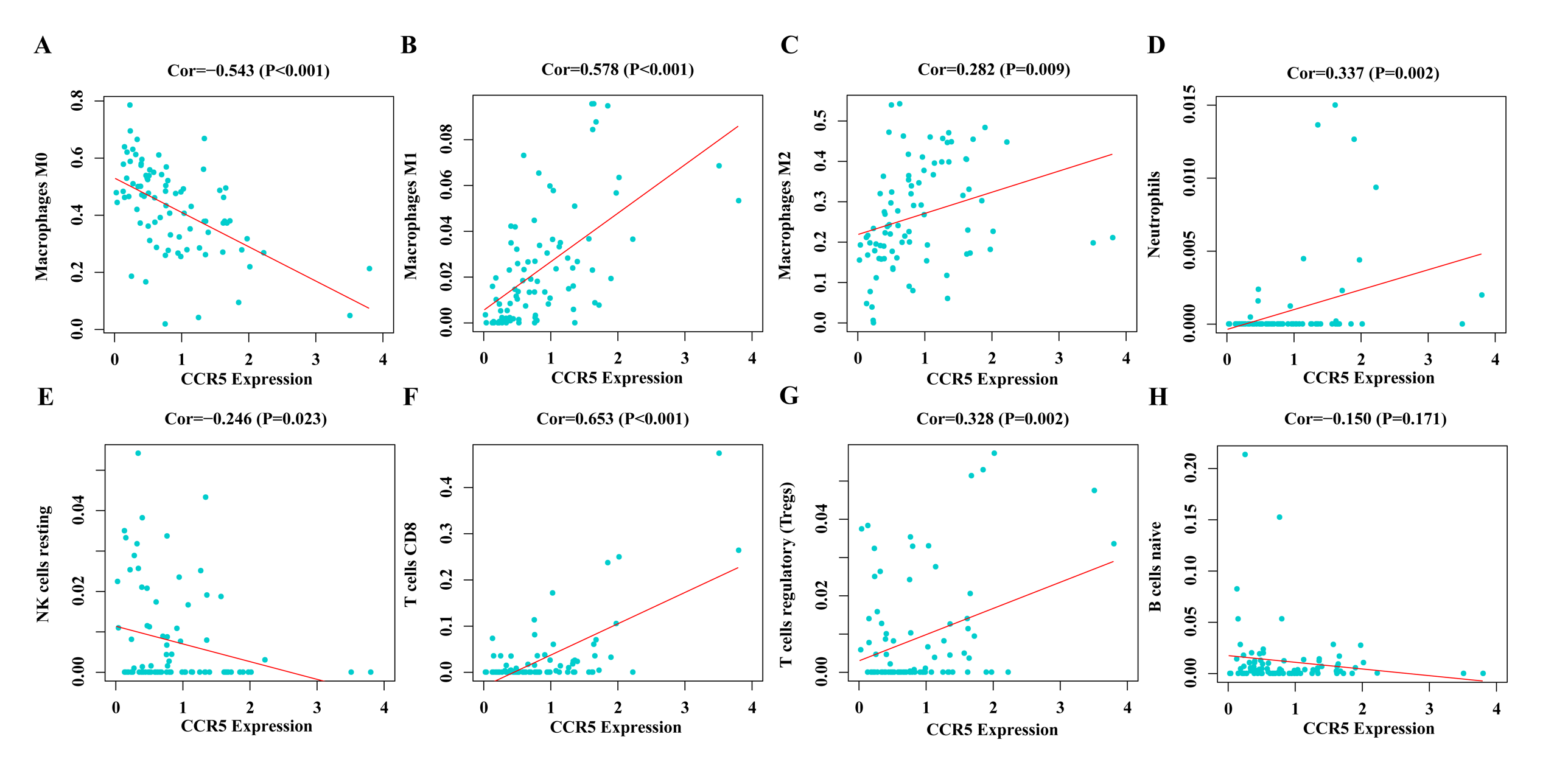

Supplement: Supplemental Digital Content [file medi-99-e23251-s006.tif]
