# Supplementary material for: Establishment of immune prognostic signature and analysis of prospective molecular mechanisms in childhood osteosarcoma patients
Source: Medicine (Baltimore). 2020 Nov 13;99(46):e23251. doi: 10.1097/MD.0000000000023251 (PMC7668544; doi:10.1097/MD.0000000000023251)
Supplement: Supplemental Digital Content [file medi-99-e23251-s007.docx]

| Table S1. Details of immune-related genes in CCLS and CCRs. |
| --- |
| \| Symbol \| ID \| Name \| Synonyms \| Chromosome \| Category \| \| --- \| --- \| --- \| --- \| --- \| --- \| |
| \| CCL13 \| 6357 \| chemokine (C-C motif) ligand 13 \| CKb10\|MCP-4\|MGC17134\|NCC-1\|NCC1\|SCYA13\|SCYL1 \| 17 \|  \| Antimicrobials \| \| --- \| --- \| --- \| --- \| --- \| --- \| --- \| \| CCL1 \| 6346 \| chemokine (C-C motif) ligand 1 \| I-309\|P500\|SCYA1\|SISe\|TCA3 \| 17 \|  \| Antimicrobials \| \| CCL8 \| 6355 \| chemokine (C-C motif) ligand 8 \| HC14\|MCP-2\|MCP2\|SCYA10\|SCYA8 \| 17 \|  \| Antimicrobials \| \| CCR10 \| 2826 \| chemokine (C-C motif) receptor 10 \| GPR2 \| 17 \|  \| Antimicrobials \| \| CCL20 \| 6364 \| chemokine (C-C motif) ligand 20 \| CKb4\|LARC\|MIP-3a\|MIP3A\|SCYA20\|ST38 \| 2 \|  \| Antimicrobials \| \| CCL5 \| 6352 \| chemokine (C-C motif) ligand 5 \| D17S136E\|MGC17164\|RANTES\|SCYA5\|SISd\|TCP228 \| 17 \|  \| Antimicrobials \| \| CCR6 \| 1235 \| chemokine (C-C motif) receptor 6 \| BN-1\|CD196\|CKR-L3\|CKR6\|CKRL3\|CMKBR6\|DCR2\|DRY-6\|GPR-CY4\|GPR29\|GPRCY4\|STRL22 \| 6 \|  \| Antimicrobials \| \| CCL4 \| 6351 \| chemokine (C-C motif) ligand 4 \| ACT2\|AT744.1\|G-26\|LAG1\|MGC104418\|MGC126025\|MGC126026\|MIP-1-beta\|MIP1B\|MIP1B1\|SCYA2\|SCYA4 \| 17 \|  \| Antimicrobials \| \| CCL28 \| 56477 \| chemokine (C-C motif) ligand 28 \| CCK1\|MEC\|MGC71902\|SCYA28 \| 5 \|  \| Antimicrobials \| \| CCL15 \| 6359 \| chemokine (C-C motif) ligand 15 \| HCC-2\|HMRP-2B\|LKN1\|Lkn-1\|MIP-1d\|MIP-5\|NCC-3\|NCC3\|SCYA15\|SCYL3\|SY15 \| 17 \|  \| Antimicrobials \| \| CCL14 \| 6358 \| chemokine (C-C motif) ligand 14 \| CC-1\|CC-3\|CKb1\|FLJ16015\|HCC-1\|HCC-3\|MCIF\|NCC-2\|NCC2\|SCYA14\|SCYL2\|SY14 \| 17 \|  \| Antimicrobials \| \| CCL4 \| 6351 \| chemokine (C-C motif) ligand 4 \| ACT2\|AT744.1\|G-26\|LAG1\|MGC104418\|MGC126025\|MGC126026\|MIP-1-beta\|MIP1B\|MIP1B1\|SCYA2\|SCYA4 \| 17 \|  \| Antimicrobials \| \| CCL16 \| 6360 \| chemokine (C-C motif) ligand 16 \| CKb12\|HCC-4\|ILINCK\|LCC-1\|LEC\|LMC\|MGC117051\|Mtn-1\|NCC-4\|NCC4\|SCYA16\|SCYL4 \| 17 \|  \| Antimicrobials \| \| CCL19 \| 6363 \| chemokine (C-C motif) ligand 19 \| CKb11\|ELC\|MGC34433\|MIP-3b\|MIP3B\|SCYA19 \| 9 \|  \| Antimicrobials \| \| CCL13 \| 6357 \| chemokine (C-C motif) ligand 13 \| CKb10\|MCP-4\|MGC17134\|NCC-1\|NCC1\|SCYA13\|SCYL1 \| 17 \|  \| Antimicrobials \| \| CCL18 \| 6362 \| chemokine (C-C motif) ligand 18 \| AMAC-1\|AMAC1\|CKb7\|DC-CK1\|DCCK1\|MIP-4\|PARC\|SCYA18 \| 17 \|  \| Antimicrobials \| \| CCL17 \| 6361 \| chemokine (C-C motif) ligand 17 \| A-152E5.3\|ABCD-2\|MGC138271\|MGC138273\|SCYA17\|TARC \| 16 \|  \| Antimicrobials \| \| CCL26 \| 10344 \| chemokine (C-C motif) ligand 26 \| IMAC\|MGC126714\|MIP-4a\|MIP-4alpha\|SCYA26\|TSC-1 \| 7 \|  \| Antimicrobials \| \| CCL22 \| 6367 \| chemokine (C-C motif) ligand 22 \| A-152E5.1\|ABCD-1\|DC/B-CK\|MDC\|MGC34554\|SCYA22\|STCP-1 \| 16 \|  \| Antimicrobials \| \| CCR3 \| 1232 \| chemokine (C-C motif) receptor 3 \| CC-CKR-3\|CD193\|CKR3\|CMKBR3\|MGC102841 \| 3 \|  \| Antimicrobials \| \| CCL28 \| 56477 \| chemokine (C-C motif) ligand 28 \| CCK1\|MEC\|MGC71902\|SCYA28 \| 5 \|  \| Antimicrobials \| \| CCR7 \| 1236 \| chemokine (C-C motif) receptor 7 \| BLR2\|CD197\|CDw197\|CMKBR7\|EBI1 \| 17 \|  \| Antimicrobials \| \| CCL27 \| 10850 \| chemokine (C-C motif) ligand 27 \| ALP\|CTACK\|CTAK\|ESKINE\|ILC\|PESKY\|SCYA27 \| 9 \|  \| Antimicrobials \| \| CCR8 \| 1237 \| chemokine (C-C motif) receptor 8 \| CDw198\|CKR-L1\|CKRL1\|CMKBR8\|CMKBRL2\|CY6\|GPR-CY6\|MGC129966\|MGC129973\|TER1 \| 3 \|  \| Antimicrobials \| \| CCR10 \| 2826 \| chemokine (C-C motif) receptor 10 \| GPR2 \| 17 \|  \| Antimicrobials \| \| CCL2 \| 6347 \| chemokine (C-C motif) ligand 2 \| GDCF-2\|HC11\|HSMCR30\|MCAF\|MCP-1\|MCP1\|MGC9434\|SCYA2\|SMC-CF \| 17 \|  \| Antimicrobials \| \| CCL21 \| 6366 \| chemokine (C-C motif) ligand 21 \| 6Ckine\|CKb9\|ECL\|MGC34555\|SCYA21\|SLC\|TCA4 \| 9 \|  \| Antimicrobials \| \| CCL7 \| 6354 \| chemokine (C-C motif) ligand 7 \| FIC\|MARC\|MCP-3\|MCP3\|MGC138463\|MGC138465\|NC28\|SCYA6\|SCYA7 \| 17 \|  \| Antimicrobials \| \| CCL5 \| 6352 \| chemokine (C-C motif) ligand 5 \| D17S136E\|MGC17164\|RANTES\|SCYA5\|SISd\|TCP228 \| 17 \|  \| Antimicrobials \| \| CCL3 \| 6348 \| chemokine (C-C motif) ligand 3 \| G0S19-1\|LD78ALPHA\|MIP-1-alpha\|MIP1A\|SCYA3 \| 17 \|  \| Antimicrobials \| \| CCL20 \| 6364 \| chemokine (C-C motif) ligand 20 \| CKb4\|LARC\|MIP-3a\|MIP3A\|SCYA20\|ST38 \| 2 \|  \| Antimicrobials \| \| CCL11 \| 6356 \| chemokine (C-C motif) ligand 11 \| MGC22554\|SCYA11 \| 17 \|  \| Antimicrobials \| \| CCR5 \| 1234 \| chemokine (C-C motif) receptor 5 \| CC-CKR-5\|CCCKR5\|CD195\|CKR-5\|CKR5\|CMKBR5\|FLJ78003\|IDDM22 \| 3 \|  \| Antimicrobials \| \| CCL23 \| 6368 \| chemokine (C-C motif) ligand 23 \| CK-BETA-8\|CKb8\|Ckb-8\|Ckb-8-1\|MIP-3\|MIP3\|MPIF-1\|SCYA23 \| 17 \|  \| Antimicrobials \| \| CCL25 \| 6370 \| chemokine (C-C motif) ligand 25 \| Ckb15\|MGC150327\|SCYA25\|TECK \| 19 \|  \| Antimicrobials \| \| CCL1 \| 6346 \| chemokine (C-C motif) ligand 1 \| I-309\|P500\|SCYA1\|SISe\|TCA3 \| 17 \|  \| Antimicrobials \| \| CCL8 \| 6355 \| chemokine (C-C motif) ligand 8 \| HC14\|MCP-2\|MCP2\|SCYA10\|SCYA8 \| 17 \|  \| Antimicrobials \| \| CCL3L1 \| 6349 \| chemokine (C-C motif) ligand 3-like 1 \| 464.2\|D17S1718\|G0S19-2\|LD78\|LD78BETA\|MGC104178\|MGC12815\|MGC182017\|MIP1AP\|SCYA3L\|SCYA3L1 \| 17 \|  \| Antimicrobials \| \| CCR1 \| 1230 \| chemokine (C-C motif) receptor 1 \| CD191\|CKR-1\|CKR1\|CMKBR1\|HM145\|MIP1aR\|SCYAR1 \| 3 \|  \| Antimicrobials \| \| CCL24 \| 6369 \| chemokine (C-C motif) ligand 24 \| Ckb-6\|MPIF-2\|MPIF2\|SCYA24 \| 7 \|  \| Antimicrobials \| \| CCR4 \| 1233 \| chemokine (C-C motif) receptor 4 \| CC-CKR-4\|CD194\|CKR4\|CMKBR4\|ChemR13\|HGCN:14099\|K5-5\|MGC88293 \| 3 \|  \| Antimicrobials \| \| CCL1 \| 6346 \| chemokine (C-C motif) ligand 1 \| I-309\|P500\|SCYA1\|SISe\|TCA3 \| 17 \|  \| Chemokines \| \| CCL11 \| 6356 \| chemokine (C-C motif) ligand 11 \| MGC22554\|SCYA11 \| 17 \|  \| Chemokines \| \| CCL13 \| 6357 \| chemokine (C-C motif) ligand 13 \| CKb10\|MCP-4\|MGC17134\|NCC-1\|NCC1\|SCYA13\|SCYL1 \| 17 \|  \| Chemokines \| \| CCL14 \| 6358 \| chemokine (C-C motif) ligand 14 \| CC-1\|CC-3\|CKb1\|FLJ16015\|HCC-1\|HCC-3\|MCIF\|NCC-2\|NCC2\|SCYA14\|SCYL2\|SY14 \| 17 \|  \| Chemokines \| \| CCL15 \| 6359 \| chemokine (C-C motif) ligand 15 \| HCC-2\|HMRP-2B\|LKN1\|Lkn-1\|MIP-1d\|MIP-5\|NCC-3\|NCC3\|SCYA15\|SCYL3\|SY15 \| 17 \|  \| Chemokines \| \| CCL16 \| 6360 \| chemokine (C-C motif) ligand 16 \| CKb12\|HCC-4\|ILINCK\|LCC-1\|LEC\|LMC\|MGC117051\|Mtn-1\|NCC-4\|NCC4\|SCYA16\|SCYL4 \| 17 \|  \| Chemokines \| \| CCL17 \| 6361 \| chemokine (C-C motif) ligand 17 \| A-152E5.3\|ABCD-2\|MGC138271\|MGC138273\|SCYA17\|TARC \| 16 \|  \| Chemokines \| \| CCL18 \| 6362 \| chemokine (C-C motif) ligand 18 \| AMAC-1\|AMAC1\|CKb7\|DC-CK1\|DCCK1\|MIP-4\|PARC\|SCYA18 \| 17 \|  \| Chemokines \| \| CCL19 \| 6363 \| chemokine (C-C motif) ligand 19 \| CKb11\|ELC\|MGC34433\|MIP-3b\|MIP3B\|SCYA19 \| 9 \|  \| Chemokines \| \| CCL2 \| 6347 \| chemokine (C-C motif) ligand 2 \| GDCF-2\|HC11\|HSMCR30\|MCAF\|MCP-1\|MCP1\|MGC9434\|SCYA2\|SMC-CF \| 17 \|  \| Chemokines \| \| CCL20 \| 6364 \| chemokine (C-C motif) ligand 20 \| CKb4\|LARC\|MIP-3a\|MIP3A\|SCYA20\|ST38 \| 2 \|  \| Chemokines \| \| CCL21 \| 6366 \| chemokine (C-C motif) ligand 21 \| 6Ckine\|CKb9\|ECL\|MGC34555\|SCYA21\|SLC\|TCA4 \| 9 \|  \| Chemokines \| \| CCL22 \| 6367 \| chemokine (C-C motif) ligand 22 \| A-152E5.1\|ABCD-1\|DC/B-CK\|MDC\|MGC34554\|SCYA22\|STCP-1 \| 16 \|  \| Chemokines \| \| CCL23 \| 6368 \| chemokine (C-C motif) ligand 23 \| CK-BETA-8\|CKb8\|Ckb-8\|Ckb-8-1\|MIP-3\|MIP3\|MPIF-1\|SCYA23 \| 17 \|  \| Chemokines \| \| CCL24 \| 6369 \| chemokine (C-C motif) ligand 24 \| Ckb-6\|MPIF-2\|MPIF2\|SCYA24 \| 7 \|  \| Chemokines \| \| CCL25 \| 6370 \| chemokine (C-C motif) ligand 25 \| Ckb15\|MGC150327\|SCYA25\|TECK \| 19 \|  \| Chemokines \| \| CCL26 \| 10344 \| chemokine (C-C motif) ligand 26 \| IMAC\|MGC126714\|MIP-4a\|MIP-4alpha\|SCYA26\|TSC-1 \| 7 \|  \| Chemokines \| \| CCL27 \| 10850 \| chemokine (C-C motif) ligand 27 \| ALP\|CTACK\|CTAK\|ESKINE\|ILC\|PESKY\|SCYA27 \| 9 \|  \| Chemokines \| \| CCL28 \| 56477 \| chemokine (C-C motif) ligand 28 \| CCK1\|MEC\|MGC71902\|SCYA28 \| 5 \|  \| Chemokines \| \| CCL3 \| 6348 \| chemokine (C-C motif) ligand 3 \| G0S19-1\|LD78ALPHA\|MIP-1-alpha\|MIP1A\|SCYA3 \| 17 \|  \| Chemokines \| \| CCL3L1 \| 6349 \| chemokine (C-C motif) ligand 3-like 1 \| 464.2\|D17S1718\|G0S19-2\|LD78\|LD78BETA\|MGC104178\|MGC12815\|MGC182017\|MIP1AP\|SCYA3L\|SCYA3L1 \| 17 \|  \| Chemokines \| \| CCL4 \| 6351 \| chemokine (C-C motif) ligand 4 \| ACT2\|AT744.1\|G-26\|LAG1\|MGC104418\|MGC126025\|MGC126026\|MIP-1-beta\|MIP1B\|MIP1B1\|SCYA2\|SCYA4 \| 17 \|  \| Chemokines \| \| CCL5 \| 6352 \| chemokine (C-C motif) ligand 5 \| D17S136E\|MGC17164\|RANTES\|SCYA5\|SISd\|TCP228 \| 17 \|  \| Chemokines \| \| CCL7 \| 6354 \| chemokine (C-C motif) ligand 7 \| FIC\|MARC\|MCP-3\|MCP3\|MGC138463\|MGC138465\|NC28\|SCYA6\|SCYA7 \| 17 \|  \| Chemokines \| \| CCL8 \| 6355 \| chemokine (C-C motif) ligand 8 \| HC14\|MCP-2\|MCP2\|SCYA10\|SCYA8 \| 17 \|  \| Chemokines \| \| CCR1 \| 1230 \| chemokine (C-C motif) receptor 1 \| CD191\|CKR-1\|CKR1\|CMKBR1\|HM145\|MIP1aR\|SCYAR1 \| 3 \|  \| Chemokine_Receptors \| \| CCR10 \| 2826 \| chemokine (C-C motif) receptor 10 \| GPR2 \| 17 \|  \| Chemokine_Receptors \| \| CCR3 \| 1232 \| chemokine (C-C motif) receptor 3 \| CC-CKR-3\|CD193\|CKR3\|CMKBR3\|MGC102841 \| 3 \|  \| Chemokine_Receptors \| \| CCR4 \| 1233 \| chemokine (C-C motif) receptor 4 \| CC-CKR-4\|CD194\|CKR4\|CMKBR4\|ChemR13\|HGCN:14099\|K5-5\|MGC88293 \| 3 \|  \| Chemokine_Receptors \| \| CCR5 \| 1234 \| chemokine (C-C motif) receptor 5 \| CC-CKR-5\|CCCKR5\|CD195\|CKR-5\|CKR5\|CMKBR5\|FLJ78003\|IDDM22 \| 3 \|  \| Chemokine_Receptors \| \| CCR6 \| 1235 \| chemokine (C-C motif) receptor 6 \| BN-1\|CD196\|CKR-L3\|CKR6\|CKRL3\|CMKBR6\|DCR2\|DRY-6\|GPR-CY4\|GPR29\|GPRCY4\|STRL22 \| 6 \|  \| Chemokine_Receptors \| \| CCR7 \| 1236 \| chemokine (C-C motif) receptor 7 \| BLR2\|CD197\|CDw197\|CMKBR7\|EBI1 \| 17 \|  \| Chemokine_Receptors \| \| CCR8 \| 1237 \| chemokine (C-C motif) receptor 8 \| CDw198\|CKR-L1\|CKRL1\|CMKBR8\|CMKBRL2\|CY6\|GPR-CY6\|MGC129966\|MGC129973\|TER1 \| 3 \|  \| Chemokine_Receptors \| \| CCR9 \| 10803 \| chemokine (C-C motif) receptor 9 \| CDw199\|GPR-9-6\|GPR28 \| 3 \|  \| Chemokine_Receptors \| \| CCL1 \| 6346 \| chemokine (C-C motif) ligand 1 \| I-309\|P500\|SCYA1\|SISe\|TCA3 \| 17 \|  \| Cytokines \| \| CCL11 \| 6356 \| chemokine (C-C motif) ligand 11 \| MGC22554\|SCYA11 \| 17 \|  \| Cytokines \| \| CCL13 \| 6357 \| chemokine (C-C motif) ligand 13 \| CKb10\|MCP-4\|MGC17134\|NCC-1\|NCC1\|SCYA13\|SCYL1 \| 17 \|  \| Cytokines \| \| CCL14 \| 6358 \| chemokine (C-C motif) ligand 14 \| CC-1\|CC-3\|CKb1\|FLJ16015\|HCC-1\|HCC-3\|MCIF\|NCC-2\|NCC2\|SCYA14\|SCYL2\|SY14 \| 17 \|  \| Cytokines \| \| CCL15 \| 6359 \| chemokine (C-C motif) ligand 15 \| HCC-2\|HMRP-2B\|LKN1\|Lkn-1\|MIP-1d\|MIP-5\|NCC-3\|NCC3\|SCYA15\|SCYL3\|SY15 \| 17 \|  \| Cytokines \| \| CCL16 \| 6360 \| chemokine (C-C motif) ligand 16 \| CKb12\|HCC-4\|ILINCK\|LCC-1\|LEC\|LMC\|MGC117051\|Mtn-1\|NCC-4\|NCC4\|SCYA16\|SCYL4 \| 17 \|  \| Cytokines \| \| CCL17 \| 6361 \| chemokine (C-C motif) ligand 17 \| A-152E5.3\|ABCD-2\|MGC138271\|MGC138273\|SCYA17\|TARC \| 16 \|  \| Cytokines \| \| CCL18 \| 6362 \| chemokine (C-C motif) ligand 18 \| AMAC-1\|AMAC1\|CKb7\|DC-CK1\|DCCK1\|MIP-4\|PARC\|SCYA18 \| 17 \|  \| Cytokines \| \| CCL19 \| 6363 \| chemokine (C-C motif) ligand 19 \| CKb11\|ELC\|MGC34433\|MIP-3b\|MIP3B\|SCYA19 \| 9 \|  \| Cytokines \| \| CCL2 \| 6347 \| chemokine (C-C motif) ligand 2 \| GDCF-2\|HC11\|HSMCR30\|MCAF\|MCP-1\|MCP1\|MGC9434\|SCYA2\|SMC-CF \| 17 \|  \| Cytokines \| \| CCL20 \| 6364 \| chemokine (C-C motif) ligand 20 \| CKb4\|LARC\|MIP-3a\|MIP3A\|SCYA20\|ST38 \| 2 \|  \| Cytokines \| \| CCL21 \| 6366 \| chemokine (C-C motif) ligand 21 \| 6Ckine\|CKb9\|ECL\|MGC34555\|SCYA21\|SLC\|TCA4 \| 9 \|  \| Cytokines \| \| CCL22 \| 6367 \| chemokine (C-C motif) ligand 22 \| A-152E5.1\|ABCD-1\|DC/B-CK\|MDC\|MGC34554\|SCYA22\|STCP-1 \| 16 \|  \| Cytokines \| \| CCL23 \| 6368 \| chemokine (C-C motif) ligand 23 \| CK-BETA-8\|CKb8\|Ckb-8\|Ckb-8-1\|MIP-3\|MIP3\|MPIF-1\|SCYA23 \| 17 \|  \| Cytokines \| \| CCL24 \| 6369 \| chemokine (C-C motif) ligand 24 \| Ckb-6\|MPIF-2\|MPIF2\|SCYA24 \| 7 \|  \| Cytokines \| \| CCL25 \| 6370 \| chemokine (C-C motif) ligand 25 \| Ckb15\|MGC150327\|SCYA25\|TECK \| 19 \|  \| Cytokines \| \| CCL26 \| 10344 \| chemokine (C-C motif) ligand 26 \| IMAC\|MGC126714\|MIP-4a\|MIP-4alpha\|SCYA26\|TSC-1 \| 7 \|  \| Cytokines \| \| CCL27 \| 10850 \| chemokine (C-C motif) ligand 27 \| ALP\|CTACK\|CTAK\|ESKINE\|ILC\|PESKY\|SCYA27 \| 9 \|  \| Cytokines \| \| CCL28 \| 56477 \| chemokine (C-C motif) ligand 28 \| CCK1\|MEC\|MGC71902\|SCYA28 \| 5 \|  \| Cytokines \| \| CCL3 \| 6348 \| chemokine (C-C motif) ligand 3 \| G0S19-1\|LD78ALPHA\|MIP-1-alpha\|MIP1A\|SCYA3 \| 17 \|  \| Cytokines \| \| CCL3L1 \| 6349 \| chemokine (C-C motif) ligand 3-like 1 \| 464.2\|D17S1718\|G0S19-2\|LD78\|LD78BETA\|MGC104178\|MGC12815\|MGC182017\|MIP1AP\|SCYA3L\|SCYA3L1 \| 17 \|  \| Cytokines \| \| CCL4 \| 6351 \| chemokine (C-C motif) ligand 4 \| ACT2\|AT744.1\|G-26\|LAG1\|MGC104418\|MGC126025\|MGC126026\|MIP-1-beta\|MIP1B\|MIP1B1\|SCYA2\|SCYA4 \| 17 \|  \| Cytokines \| \| CCL5 \| 6352 \| chemokine (C-C motif) ligand 5 \| D17S136E\|MGC17164\|RANTES\|SCYA5\|SISd\|TCP228 \| 17 \|  \| Cytokines \| \| CCL7 \| 6354 \| chemokine (C-C motif) ligand 7 \| FIC\|MARC\|MCP-3\|MCP3\|MGC138463\|MGC138465\|NC28\|SCYA6\|SCYA7 \| 17 \|  \| Cytokines \| \| CCL8 \| 6355 \| chemokine (C-C motif) ligand 8 \| HC14\|MCP-2\|MCP2\|SCYA10\|SCYA8 \| 17 \|  \| Cytokines \| \| CCR1 \| 1230 \| chemokine (C-C motif) receptor 1 \| CD191\|CKR-1\|CKR1\|CMKBR1\|HM145\|MIP1aR\|SCYAR1 \| 3 \|  \| Cytokine_Receptors \| \| CCR10 \| 2826 \| chemokine (C-C motif) receptor 10 \| GPR2 \| 17 \|  \| Cytokine_Receptors \| \| CCR3 \| 1232 \| chemokine (C-C motif) receptor 3 \| CC-CKR-3\|CD193\|CKR3\|CMKBR3\|MGC102841 \| 3 \|  \| Cytokine_Receptors \| \| CCR4 \| 1233 \| chemokine (C-C motif) receptor 4 \| CC-CKR-4\|CD194\|CKR4\|CMKBR4\|ChemR13\|HGCN:14099\|K5-5\|MGC88293 \| 3 \|  \| Cytokine_Receptors \| \| CCR5 \| 1234 \| chemokine (C-C motif) receptor 5 \| CC-CKR-5\|CCCKR5\|CD195\|CKR-5\|CKR5\|CMKBR5\|FLJ78003\|IDDM22 \| 3 \|  \| Cytokine_Receptors \| \| CCR6 \| 1235 \| chemokine (C-C motif) receptor 6 \| BN-1\|CD196\|CKR-L3\|CKR6\|CKRL3\|CMKBR6\|DCR2\|DRY-6\|GPR-CY4\|GPR29\|GPRCY4\|STRL22 \| 6 \|  \| Cytokine_Receptors \| \| CCR7 \| 1236 \| chemokine (C-C motif) receptor 7 \| BLR2\|CD197\|CDw197\|CMKBR7\|EBI1 \| 17 \|  \| Cytokine_Receptors \| \| CCR8 \| 1237 \| chemokine (C-C motif) receptor 8 \| CDw198\|CKR-L1\|CKRL1\|CMKBR8\|CMKBRL2\|CY6\|GPR-CY6\|MGC129966\|MGC129973\|TER1 \| 3 \|  \| Cytokine_Receptors \| \| CCR9 \| 10803 \| chemokine (C-C motif) receptor 9 \| CDw199\|GPR-9-6\|GPR28 \| 3 \|  \| Cytokine_Receptors \| |

CCL, C-C motif chemokine ligand genes; CCR, C-C motif chemokine receptors.
