# Supplementary material for: Establishment of immune prognostic signature and analysis of prospective molecular mechanisms in childhood osteosarcoma patients
Source: Medicine (Baltimore). 2020 Nov 13;99(46):e23251. doi: 10.1097/MD.0000000000023251 (PMC7668544; doi:10.1097/MD.0000000000023251)
Supplement: Supplemental Digital Content [file medi-99-e23251-s008.docx]

| \| Table S2. Part of the results of GSEA analysis in the high expression group of CCL5. \|  \|  \|  \|  \|  \|  \|  \| \| --- \| --- \| --- \| --- \| --- \| --- \| --- \| --- \| \| GS<br> follow link to MSigDB \| SIZE \| ES \| NES \| NOM  p-val \| FDR  q-val \| FWER  p-val \| RANK AT MAX \| |
| --- | --- | --- | --- | --- | --- | --- | --- | --- | --- | --- | --- | --- | --- | --- | --- | --- |
| \| REACTOME_NEUTROPHIL_DEGRANULATION \| 476 \| 0.6658652 \| 2.7406673 \| <0.001 \| <0.001 \| 0 \| 8170 \| \| --- \| --- \| --- \| --- \| --- \| --- \| --- \| --- \| \| RODWELL_AGING_KIDNEY_UP \| 492 \| 0.75047714 \| 2.719833 \| <0.001 \| <0.001 \| 0 \| 5146 \| \| REACTOME_SIGNALING_BY_INTERLEUKINS \| 448 \| 0.6231729 \| 2.7182305 \| <0.001 \| <0.001 \| 0 \| 7949 \| \| REACTOME_TNFR2_NON_CANONICAL_NF_KB_PATHWAY \| 101 \| 0.7136341 \| 2.699469 \| <0.001 \| <0.001 \| 0 \| 8170 \| \| REACTOME_INTERLEUKIN_12_FAMILY_SIGNALING \| 57 \| 0.66489697 \| 2.6911724 \| <0.001 \| <0.001 \| 0 \| 10801 \| \| JOHNSTONE_PARVB_TARGETS_2_UP \| 142 \| 0.67130315 \| 2.6653368 \| <0.001 \| <0.001 \| 0.001 \| 7152 \| \| LI_INDUCED_T_TO_NATURAL_KILLER_UP \| 313 \| 0.70874774 \| 2.6580408 \| <0.001 \| <0.001 \| 0.001 \| 7278 \| \| REACTOME_INTERFERON_SIGNALING \| 199 \| 0.7251938 \| 2.6495993 \| <0.001 \| <0.001 \| 0.001 \| 3967 \| \| HUANG_GATA2_TARGETS_UP \| 146 \| 0.75054693 \| 2.636594 \| <0.001 \| <0.001 \| 0.001 \| 4356 \| \| ROSS_AML_WITH_MLL_FUSIONS \| 80 \| 0.72128576 \| 2.6312246 \| <0.001 \| <0.001 \| 0.001 \| 5044 \| \| JISON_SICKLE_CELL_DISEASE_UP \| 180 \| 0.7273882 \| 2.6256878 \| <0.001 \| <0.001 \| 0.001 \| 5917 \| \| DIAZ_CHRONIC_MEYLOGENOUS_LEUKEMIA_DN \| 116 \| 0.73007715 \| 2.624859 \| <0.001 \| <0.001 \| 0.001 \| 3762 \| \| RUTELLA_RESPONSE_TO_CSF2RB_AND_IL4_DN \| 315 \| 0.72826946 \| 2.6154165 \| <0.001 \| <0.001 \| 0.001 \| 5119 \| \| REACTOME_CLEC7A_DECTIN_1_SIGNALING \| 100 \| 0.6650851 \| 2.6132588 \| <0.001 \| <0.001 \| 0.001 \| 8920 \| \| SANSOM_APC_TARGETS_DN \| 338 \| 0.64005446 \| 2.6124792 \| <0.001 \| <0.001 \| 0.001 \| 6464 \| \| FURUKAWA_DUSP6_TARGETS_PCI35_UP \| 71 \| 0.75976515 \| 2.6072176 \| <0.001 \| <0.001 \| 0.001 \| 5603 \| \| GNATENKO_PLATELET_SIGNATURE \| 40 \| 0.72498584 \| 2.60616 \| <0.001 \| <0.001 \| 0.001 \| 8647 \| \| QI_PLASMACYTOMA_UP \| 254 \| 0.76830614 \| 2.5966508 \| <0.001 \| <0.001 \| 0.001 \| 7041 \| \| SMID_BREAST_CANCER_NORMAL_LIKE_UP \| 481 \| 0.7607734 \| 2.5964234 \| <0.001 \| <0.001 \| 0.001 \| 4875 \| \| WALLACE_PROSTATE_CANCER_RACE_UP \| 299 \| 0.8480021 \| 2.594756 \| <0.001 \| <0.001 \| 0.001 \| 3437 \| \| BROWN_MYELOID_CELL_DEVELOPMENT_UP \| 160 \| 0.7427872 \| 2.593547 \| <0.001 \| <0.001 \| 0.002 \| 5426 \| \| REACTOME_C_TYPE_LECTIN_RECEPTORS_CLRS \| 140 \| 0.5783221 \| 2.5918512 \| <0.001 \| <0.001 \| 0.002 \| 8920 \| \| HELLER_SILENCED_BY_METHYLATION_UP \| 277 \| 0.7024681 \| 2.583379 \| <0.001 \| <0.001 \| 0.003 \| 5142 \| \| REACTOME_TCR_SIGNALING \| 122 \| 0.7335671 \| 2.5789747 \| <0.001 \| <0.001 \| 0.003 \| 5720 \| \| BOSCO_TH1_CYTOTOXIC_MODULE \| 113 \| 0.8110857 \| 2.5724843 \| <0.001 \| <0.001 \| 0.004 \| 4795 \| \| RUTELLA_RESPONSE_TO_HGF_VS_CSF2RB_AND_IL4_UP \| 405 \| 0.6965211 \| 2.5669248 \| <0.001 \| <0.001 \| 0.005 \| 5119 \| \| KEGG_LYSOSOME \| 121 \| 0.7230144 \| 2.566638 \| <0.001 \| <0.001 \| 0.005 \| 5950 \| \| FERRANDO_T_ALL_WITH_MLL_ENL_FUSION_UP \| 89 \| 0.69210213 \| 2.561328 \| <0.001 \| <0.001 \| 0.005 \| 4276 \| \| THUM_SYSTOLIC_HEART_FAILURE_UP \| 424 \| 0.68115777 \| 2.559901 \| <0.001 \| <0.001 \| 0.005 \| 4359 \| \| REACTOME_COSTIMULATION_BY_THE_CD28_FAMILY \| 72 \| 0.8154846 \| 2.551665 \| <0.001 \| <0.001 \| 0.007 \| 3307 \| \| KEGG_VIRAL_MYOCARDITIS \| 68 \| 0.80528045 \| 2.5496762 \| <0.001 \| <0.001 \| 0.007 \| 2177 \| \| TONKS_TARGETS_OF_RUNX1_RUNX1T1_FUSION_ERYTHROCYTE_UP \| 157 \| 0.72070265 \| 2.5484242 \| <0.001 \| <0.001 \| 0.007 \| 5161 \| \| HOFFMANN_PRE_BI_TO_LARGE_PRE_BII_LYMPHOCYTE_DN \| 74 \| 0.65993756 \| 2.5479112 \| <0.001 \| <0.001 \| 0.007 \| 3701 \| \| KEGG_CELL_ADHESION_MOLECULES_CAMS \| 131 \| 0.7443335 \| 2.5455766 \| <0.001 \| <0.001 \| 0.007 \| 3233 \| \| WANG_ESOPHAGUS_CANCER_VS_NORMAL_UP \| 119 \| 0.6862354 \| 2.5426774 \| <0.001 \| <0.001 \| 0.007 \| 3888 \| \| REACTOME_ANTIGEN_PROCESSING_CROSS_PRESENTATION \| 99 \| 0.76337475 \| 2.5404763 \| <0.001 \| <0.001 \| 0.007 \| 5720 \| \| LU_IL4_SIGNALING \| 89 \| 0.7306962 \| 2.5391643 \| <0.001 \| <0.001 \| 0.007 \| 5334 \| \| MULLIGHAN_MLL_SIGNATURE_1_UP \| 381 \| 0.6436148 \| 2.5364554 \| <0.001 \| <0.001 \| 0.007 \| 7585 \| \| POOLA_INVASIVE_BREAST_CANCER_UP \| 291 \| 0.7725605 \| 2.5281277 \| <0.001 \| <0.001 \| 0.008 \| 4105 \| \| SHIN_B_CELL_LYMPHOMA_CLUSTER_8 \| 36 \| 0.7919552 \| 2.527313 \| <0.001 \| <0.001 \| 0.008 \| 2564 \| \| REACTOME_DOWNSTREAM_SIGNALING_EVENTS_OF_B_CELL_RECEPTOR_BCR \| 81 \| 0.65766734 \| 2.5266354 \| <0.001 \| <0.001 \| 0.008 \| 8917 \| \| GRAESSMANN_RESPONSE_TO_MC_AND_SERUM_DEPRIVATION_UP \| 211 \| 0.66209245 \| 2.5242727 \| <0.001 \| <0.001 \| 0.009 \| 5982 \| \| REACTOME_LEISHMANIA_INFECTION \| 306 \| 0.6332108 \| 2.5222497 \| <0.001 \| <0.001 \| 0.01 \| 5191 \| \| RASHI_RESPONSE_TO_IONIZING_RADIATION_6 \| 83 \| 0.777803 \| 2.521978 \| <0.001 \| <0.001 \| 0.01 \| 5406 \| \| REACTOME_INTERFERON_GAMMA_SIGNALING \| 91 \| 0.8306679 \| 2.5199327 \| <0.001 \| <0.001 \| 0.01 \| 4159 \| \| FULCHER_INFLAMMATORY_RESPONSE_LECTIN_VS_LPS_DN \| 442 \| 0.723673 \| 2.5179145 \| <0.001 \| <0.001 \| 0.01 \| 5142 \| \| REACTOME_INTERLEUKIN_12_SIGNALING \| 47 \| 0.63185924 \| 2.5149002 \| <0.001 \| <0.001 \| 0.01 \| 10574 \| \| RUTELLA_RESPONSE_TO_HGF_DN \| 233 \| 0.66071594 \| 2.5122163 \| <0.001 \| <0.001 \| 0.011 \| 4942 \| \| JOHNSTONE_PARVB_TARGETS_3_UP \| 426 \| 0.61151224 \| 2.510833 \| <0.001 \| <0.001 \| 0.011 \| 8979 \| \| VERHAAK_AML_WITH_NPM1_MUTATED_UP \| 178 \| 0.7670353 \| 2.5094368 \| <0.001 \| <0.001 \| 0.011 \| 4307 \| \| CHYLA_CBFA2T3_TARGETS_UP \| 376 \| 0.62223315 \| 2.506299 \| <0.001 \| <0.001 \| 0.011 \| 7114 \| \| KEGG_CHEMOKINE_SIGNALING_PATHWAY \| 188 \| 0.667547 \| 2.505199 \| <0.001 \| <0.001 \| 0.012 \| 5191 \| \| BERTUCCI_MEDULLARY_VS_DUCTAL_BREAST_CANCER_UP \| 202 \| 0.71471155 \| 2.5036201 \| <0.001 \| <0.001 \| 0.012 \| 4509 \| \| MULLIGHAN_MLL_SIGNATURE_2_UP \| 419 \| 0.63604295 \| 2.5011005 \| <0.001 \| <0.001 \| 0.012 \| 7585 \| \| REACTOME_INTERLEUKIN_1_FAMILY_SIGNALING \| 139 \| 0.57143545 \| 2.5004303 \| <0.001 \| <0.001 \| 0.012 \| 8647 \| \| REACTOME_CELL_SURFACE_INTERACTIONS_AT_THE_VASCULAR_WALL \| 194 \| 0.7467604 \| 2.5001502 \| <0.001 \| <0.001 \| 0.012 \| 4345 \| \| MORI_PRE_BI_LYMPHOCYTE_DN \| 81 \| 0.75453234 \| 2.49649 \| <0.001 \| <0.001 \| 0.012 \| 6706 \| \| KATSANOU_ELAVL1_TARGETS_UP \| 157 \| 0.6372605 \| 2.4928317 \| <0.001 \| <0.001 \| 0.013 \| 6639 \| \| ACOSTA_PROLIFERATION_INDEPENDENT_MYC_TARGETS_DN \| 116 \| 0.65152067 \| 2.492277 \| <0.001 \| <0.001 \| 0.013 \| 7781 \| \| TAKEDA_TARGETS_OF_NUP98_HOXA9_FUSION_3D_UP \| 178 \| 0.7139672 \| 2.4918451 \| <0.001 \| <0.001 \| 0.014 \| 4375 \| \| JAATINEN_HEMATOPOIETIC_STEM_CELL_DN \| 234 \| 0.75173604 \| 2.4914482 \| <0.001 \| <0.001 \| 0.014 \| 3876 \| \| ODONNELL_TARGETS_OF_MYC_AND_TFRC_UP \| 80 \| 0.79764026 \| 2.4855018 \| <0.001 \| <0.001 \| 0.014 \| 3899 \| \| KEGG_NATURAL_KILLER_CELL_MEDIATED_CYTOTOXICITY \| 132 \| 0.67301023 \| 2.4814305 \| <0.001 \| <0.001 \| 0.014 \| 3511 \| \| SWEET_LUNG_CANCER_KRAS_UP \| 469 \| 0.540547 \| 2.4794133 \| <0.001 \| <0.001 \| 0.014 \| 9594 \| \| LINDGREN_BLADDER_CANCER_CLUSTER_2B \| 384 \| 0.6858961 \| 2.4773958 \| <0.001 \| <0.001 \| 0.015 \| 6106 \| \| LEE_EARLY_T_LYMPHOCYTE_DN \| 58 \| 0.7986897 \| 2.4752417 \| <0.001 \| 0.001037388 \| 0.015 \| 5317 \| \| KEGG_CYTOKINE_CYTOKINE_RECEPTOR_INTERACTION \| 264 \| 0.67869157 \| 2.4736838 \| <0.001 \| 0.001056077 \| 0.015 \| 4387 \| \| MIKKELSEN_MCV6_LCP_WITH_H3K4ME3 \| 162 \| 0.62597823 \| 2.4710245 \| <0.001 \| 0.001093182 \| 0.016 \| 6373 \| \| HOSHIDA_LIVER_CANCER_SUBCLASS_S1 \| 233 \| 0.703196 \| 2.4685268 \| <0.001 \| 0.001108335 \| 0.017 \| 6143 \| \| CAIRO_HEPATOBLASTOMA_CLASSES_DN \| 213 \| 0.6086851 \| 2.4663236 \| <0.001 \| 0.001155784 \| 0.017 \| 5936 \| \| TARTE_PLASMA_CELL_VS_PLASMABLAST_UP \| 393 \| 0.55926865 \| 2.465798 \| <0.001 \| 0.001139506 \| 0.017 \| 6143 \| \| REACTOME_FC_EPSILON_RECEPTOR_FCERI_SIGNALING \| 186 \| 0.7303348 \| 2.4636006 \| <0.001 \| 0.001142334 \| 0.017 \| 4959 \| \| TAKEDA_TARGETS_OF_NUP98_HOXA9_FUSION_16D_UP \| 176 \| 0.6694799 \| 2.4611483 \| <0.001 \| 0.001154284 \| 0.018 \| 4843 \| \| TONKS_TARGETS_OF_RUNX1_RUNX1T1_FUSION_HSC_DN \| 193 \| 0.69471574 \| 2.4610717 \| <0.001 \| 0.001138686 \| 0.018 \| 4982 \| \| BOSCO_ALLERGEN_INDUCED_TH2_ASSOCIATED_MODULE \| 147 \| 0.63745 \| 2.459682 \| <0.001 \| 0.001123503 \| 0.018 \| 4544 \| \| REACTOME_IMMUNOREGULATORY_INTERACTIONS_BETWEEN_A_LYMPHOID_AND_A_NON_LYMPHOID_CELL \| 187 \| 0.8626214 \| 2.4584398 \| <0.001 \| 0.00112353 \| 0.018 \| 3099 \| \| DER_IFN_GAMMA_RESPONSE_UP \| 71 \| 0.7946139 \| 2.4579732 \| <0.001 \| 0.001108938 \| 0.018 \| 6085 \| \| ICHIBA_GRAFT_VERSUS_HOST_DISEASE_35D_UP \| 143 \| 0.81390655 \| 2.457282 \| <0.001 \| 0.001111752 \| 0.019 \| 3600 \| \| LIU_VAV3_PROSTATE_CARCINOGENESIS_UP \| 87 \| 0.7710007 \| 2.4567215 \| <0.001 \| 0.001097679 \| 0.019 \| 3451 \| \| BOYLAN_MULTIPLE_MYELOMA_C_D_DN \| 277 \| 0.63640547 \| 2.455915 \| <0.001 \| 0.001100748 \| 0.019 \| 4293 \| \| MORI_IMMATURE_B_LYMPHOCYTE_UP \| 51 \| 0.8177287 \| 2.4528751 \| <0.001 \| 0.001139602 \| 0.02 \| 4056 \| \| SWEET_KRAS_ONCOGENIC_SIGNATURE \| 88 \| 0.6285213 \| 2.4522977 \| <0.001 \| 0.001125704 \| 0.02 \| 10249 \| \| PID_CXCR4_PATHWAY \| 101 \| 0.7182913 \| 2.452082 \| <0.001 \| 0.001112141 \| 0.02 \| 7700 \| \| MARKEY_RB1_ACUTE_LOF_DN \| 226 \| 0.7503257 \| 2.4511697 \| <0.001 \| 0.001112048 \| 0.02 \| 4834 \| \| MUNSHI_MULTIPLE_MYELOMA_UP \| 77 \| 0.596558 \| 2.4494588 \| <0.001 \| 0.001140824 \| 0.02 \| 5917 \| \| ZHONG_SECRETOME_OF_LUNG_CANCER_AND_FIBROBLAST \| 131 \| 0.51408017 \| 2.4490607 \| <0.001 \| 0.001127559 \| 0.02 \| 11597 \| \| MORI_MATURE_B_LYMPHOCYTE_UP \| 94 \| 0.72262067 \| 2.4461572 \| <0.001 \| 0.001142101 \| 0.021 \| 3971 \| \| NEMETH_INFLAMMATORY_RESPONSE_LPS_UP \| 80 \| 0.76011986 \| 2.4433146 \| <0.001 \| 0.001179671 \| 0.021 \| 6055 \| \| CASTELLANO_NRAS_TARGETS_UP \| 70 \| 0.7203986 \| 2.4432778 \| <0.001 \| 0.001166416 \| 0.021 \| 8326 \| \| WUNDER_INFLAMMATORY_RESPONSE_AND_CHOLESTEROL_UP \| 60 \| 0.86585146 \| 2.442784 \| <0.001 \| 0.001153456 \| 0.021 \| 1887 \| \| MARKEY_RB1_CHRONIC_LOF_DN \| 117 \| 0.7506223 \| 2.4407547 \| <0.001 \| 0.001175338 \| 0.022 \| 6411 \| \| BIDUS_METASTASIS_DN \| 149 \| 0.6625826 \| 2.4405289 \| <0.001 \| 0.001174634 \| 0.022 \| 4982 \| \| ICHIBA_GRAFT_VERSUS_HOST_DISEASE_D7_UP \| 114 \| 0.85648024 \| 2.4396853 \| <0.001 \| 0.001173878 \| 0.022 \| 4905 \| \| KLEIN_TARGETS_OF_BCR_ABL1_FUSION \| 42 \| 0.8013828 \| 2.4392047 \| <0.001 \| 0.001161389 \| 0.022 \| 3277 \| \| GRUETZMANN_PANCREATIC_CANCER_UP \| 352 \| 0.56641865 \| 2.4389415 \| <0.001 \| 0.001149164 \| 0.022 \| 9191 \| \| PID_IL27_PATHWAY \| 26 \| 0.85843617 \| 2.4382625 \| <0.001 \| 0.001151185 \| 0.022 \| 3584 \| \| HELLER_HDAC_TARGETS_DN \| 290 \| 0.5709147 \| 2.4379463 \| <0.001 \| 0.001139317 \| 0.022 \| 5311 \| \| GAL_LEUKEMIC_STEM_CELL_DN \| 233 \| 0.64470154 \| 2.4367256 \| <0.001 \| 0.001127691 \| 0.022 \| 2592 \| \| ZHANG_RESPONSE_TO_IKK_INHIBITOR_AND_TNF_UP \| 221 \| 0.6870154 \| 2.4358444 \| <0.001 \| 0.001125499 \| 0.022 \| 6001 \| \| MISSIAGLIA_REGULATED_BY_METHYLATION_UP \| 122 \| 0.6759416 \| 2.435804 \| <0.001 \| 0.001114244 \| 0.022 \| 7742 \| \| GO_RESPONSE_TO_VIRUS \| 324 \| 0.6841709 \| 2.7861307 \| <0.001 \| <0.001 \| 0 \| 5908 \| \| GO_T_CELL_ACTIVATION \| 466 \| 0.6941511 \| 2.738575 \| <0.001 \| <0.001 \| 0 \| 5317 \| \| GO_LEUKOCYTE_APOPTOTIC_PROCESS \| 107 \| 0.73219824 \| 2.729051 \| <0.001 \| <0.001 \| 0 \| 4202 \| \| GO_POSITIVE_REGULATION_OF_RESPONSE_TO_BIOTIC_STIMULUS \| 396 \| 0.60989803 \| 2.7265372 \| <0.001 \| <0.001 \| 0 \| 8100 \| \| GO_VESICLE_LUMEN \| 326 \| 0.60075676 \| 2.7264268 \| <0.001 \| <0.001 \| 0 \| 8595 \| \| GO_POSITIVE_REGULATION_OF_CYTOKINE_PRODUCTION \| 461 \| 0.67262685 \| 2.7221582 \| <0.001 \| <0.001 \| 0 \| 5317 \| \| GO_LEUKOCYTE_CELL_CELL_ADHESION \| 338 \| 0.7264719 \| 2.7185311 \| <0.001 \| <0.001 \| 0 \| 5604 \| \| GO_NIK_NF_KAPPAB_SIGNALING \| 177 \| 0.6171757 \| 2.7127075 \| <0.001 \| <0.001 \| 0 \| 8483 \| \| GO_REGULATION_OF_LYMPHOCYTE_ACTIVATION \| 485 \| 0.73859364 \| 2.712544 \| <0.001 \| <0.001 \| 0 \| 4546 \| \| GO_ACTIVATION_OF_INNATE_IMMUNE_RESPONSE \| 318 \| 0.6047571 \| 2.711195 \| <0.001 \| <0.001 \| 0 \| 8170 \| \| GO_REGULATION_OF_T_CELL_ACTIVATION \| 316 \| 0.73362535 \| 2.7110813 \| <0.001 \| <0.001 \| 0 \| 5317 \| \| GO_NEGATIVE_REGULATION_OF_MULTI_ORGANISM_PROCESS \| 222 \| 0.6620085 \| 2.7108724 \| <0.001 \| <0.001 \| 0 \| 5908 \| \| GO_TUMOR_NECROSIS_FACTOR_MEDIATED_SIGNALING_PATHWAY \| 161 \| 0.64671165 \| 2.7081 \| <0.001 \| <0.001 \| 0 \| 8188 \| \| GO_POSITIVE_REGULATION_OF_ADAPTIVE_IMMUNE_RESPONSE \| 104 \| 0.75030017 \| 2.7080214 \| <0.001 \| <0.001 \| 0 \| 6064 \| \| GO_AZUROPHIL_GRANULE \| 154 \| 0.671853 \| 2.7076747 \| <0.001 \| <0.001 \| 0 \| 6543 \| \| GO_POSITIVE_REGULATION_OF_CELL_CELL_ADHESION \| 256 \| 0.7399899 \| 2.7072363 \| <0.001 \| <0.001 \| 0 \| 5590 \| \| GO_DEFENSE_RESPONSE_TO_VIRUS \| 241 \| 0.70006055 \| 2.7027261 \| <0.001 \| <0.001 \| 0 \| 4425 \| \| GO_T_CELL_RECEPTOR_SIGNALING_PATHWAY \| 199 \| 0.71416265 \| 2.7024462 \| <0.001 \| <0.001 \| 0 \| 4959 \| \| GO_T_CELL_PROLIFERATION \| 185 \| 0.7543985 \| 2.6995113 \| <0.001 \| <0.001 \| 0 \| 5243 \| \| GO_RESPONSE_TO_TUMOR_NECROSIS_FACTOR \| 305 \| 0.63565266 \| 2.697748 \| <0.001 \| <0.001 \| 0 \| 7939 \| \| GO_LYMPHOCYTE_APOPTOTIC_PROCESS \| 72 \| 0.739177 \| 2.6975453 \| <0.001 \| <0.001 \| 0 \| 3998 \| \| GO_POSITIVE_REGULATION_OF_CELL_ADHESION \| 397 \| 0.68643993 \| 2.697447 \| <0.001 \| <0.001 \| 0 \| 4546 \| \| GO_LEUKOCYTE_PROLIFERATION \| 299 \| 0.7137236 \| 2.6926575 \| <0.001 \| <0.001 \| 0 \| 4546 \| \| GO_T_CELL_MEDIATED_IMMUNITY \| 100 \| 0.76792747 \| 2.6919696 \| <0.001 \| <0.001 \| 0 \| 4363 \| \| GO_REGULATION_OF_ADAPTIVE_IMMUNE_RESPONSE \| 159 \| 0.7297441 \| 2.675556 \| <0.001 \| <0.001 \| 0 \| 5873 \| \| GO_REGULATION_OF_LEUKOCYTE_PROLIFERATION \| 223 \| 0.74763566 \| 2.6712568 \| <0.001 \| <0.001 \| 0 \| 4546 \| \| GO_REGULATION_OF_LYMPHOCYTE_DIFFERENTIATION \| 170 \| 0.7090812 \| 2.6696618 \| <0.001 \| <0.001 \| 0 \| 5317 \| \| GO_ANTIGEN_PROCESSING_AND_PRESENTATION \| 224 \| 0.6983706 \| 2.6651356 \| <0.001 \| <0.001 \| 0 \| 5720 \| \| GO_REGULATION_OF_CELL_CELL_ADHESION \| 406 \| 0.67646706 \| 2.6584914 \| <0.001 \| <0.001 \| 0 \| 5604 \| \| GO_T_CELL_DIFFERENTIATION \| 239 \| 0.6877665 \| 2.6542165 \| <0.001 \| <0.001 \| 0 \| 5317 \| \| GO_IMMUNE_RESPONSE_REGULATING_CELL_SURFACE_RECEPTOR_SIGNALING_PATHWAY \| 491 \| 0.73441684 \| 2.6534026 \| <0.001 \| <0.001 \| 0 \| 4775 \| \| GO_POSITIVE_REGULATION_OF_LEUKOCYTE_CELL_CELL_ADHESION \| 216 \| 0.76794446 \| 2.6530132 \| <0.001 \| <0.001 \| 0 \| 4375 \| \| GO_REGULATION_OF_LEUKOCYTE_DIFFERENTIATION \| 274 \| 0.688481 \| 2.652094 \| <0.001 \| <0.001 \| 0 \| 5317 \| \| GO_NEGATIVE_REGULATION_OF_IMMUNE_SYSTEM_PROCESS \| 452 \| 0.6387479 \| 2.6515138 \| <0.001 \| <0.001 \| 0 \| 5317 \| \| GO_REGULATION_OF_HEMOPOIESIS \| 446 \| 0.597854 \| 2.646612 \| <0.001 \| <0.001 \| 0 \| 5322 \| \| GO_POSITIVE_REGULATION_OF_LEUKOCYTE_PROLIFERATION \| 139 \| 0.757856 \| 2.6416743 \| <0.001 \| <0.001 \| 0 \| 4546 \| \| GO_REGULATION_OF_IMMUNE_EFFECTOR_PROCESS \| 451 \| 0.7219262 \| 2.6373577 \| <0.001 \| <0.001 \| 0 \| 4387 \| \| GO_NEGATIVE_REGULATION_OF_IMMUNE_RESPONSE \| 152 \| 0.67984635 \| 2.6362011 \| <0.001 \| <0.001 \| 0 \| 2438 \| \| GO_REGULATION_OF_PRODUCTION_OF_MOLECULAR_MEDIATOR_OF_IMMUNE_RESPONSE \| 141 \| 0.68311507 \| 2.6358075 \| <0.001 \| <0.001 \| 0 \| 4387 \| \| GO_RESPONSE_TO_INTERLEUKIN_1 \| 203 \| 0.62035996 \| 2.6335323 \| <0.001 \| <0.001 \| 0 \| 8689 \| \| GO_RESPONSE_TO_INTERFERON_GAMMA \| 193 \| 0.80431676 \| 2.6310222 \| <0.001 \| <0.001 \| 0 \| 4159 \| \| GO_REGULATION_OF_LEUKOCYTE_MEDIATED_IMMUNITY \| 198 \| 0.7283358 \| 2.6293852 \| <0.001 \| <0.001 \| 0 \| 4363 \| \| GO_REGULATION_OF_T_CELL_DIFFERENTIATION \| 139 \| 0.7148525 \| 2.628685 \| <0.001 \| <0.001 \| 0 \| 5317 \| \| GO_TYPE_I_INTERFERON_PRODUCTION \| 126 \| 0.6963422 \| 2.6250036 \| <0.001 \| <0.001 \| 0 \| 5399 \| \| GO_T_CELL_APOPTOTIC_PROCESS \| 49 \| 0.73264915 \| 2.624742 \| <0.001 \| <0.001 \| 0 \| 3998 \| \| GO_NEGATIVE_REGULATION_OF_IMMUNE_EFFECTOR_PROCESS \| 120 \| 0.6823102 \| 2.6204567 \| <0.001 \| <0.001 \| 0 \| 5908 \| \| GO_REGULATION_OF_LYMPHOCYTE_MEDIATED_IMMUNITY \| 147 \| 0.7261776 \| 2.620346 \| <0.001 \| <0.001 \| 0 \| 4363 \| \| GO_CYTOKINE_PRODUCTION_INVOLVED_IN_IMMUNE_RESPONSE \| 104 \| 0.73876816 \| 2.6177945 \| <0.001 \| <0.001 \| 0 \| 4387 \| \| GO_LYMPHOCYTE_ACTIVATION_INVOLVED_IN_IMMUNE_RESPONSE \| 181 \| 0.67689776 \| 2.6177318 \| <0.001 \| <0.001 \| 0 \| 4626 \| \| GO_LEUKOCYTE_MIGRATION \| 493 \| 0.71401036 \| 2.6168463 \| <0.001 \| <0.001 \| 0 \| 4387 \| \| GO_POSITIVE_REGULATION_OF_CELL_ACTIVATION \| 383 \| 0.7677326 \| 2.6154003 \| <0.001 \| <0.001 \| 0 \| 4375 \| \| GO_LYMPHOCYTE_DIFFERENTIATION \| 353 \| 0.64831835 \| 2.6139405 \| <0.001 \| <0.001 \| 0 \| 5317 \| \| GO_ANTIGEN_PROCESSING_AND_PRESENTATION_OF_PEPTIDE_ANTIGEN \| 187 \| 0.68698364 \| 2.612253 \| <0.001 \| <0.001 \| 0 \| 5870 \| \| GO_AZUROPHIL_GRANULE_LUMEN \| 90 \| 0.6661626 \| 2.61075 \| <0.001 \| <0.001 \| 0 \| 6427 \| \| GO_POSITIVE_REGULATION_OF_IMMUNE_EFFECTOR_PROCESS \| 211 \| 0.6902996 \| 2.607688 \| <0.001 \| <0.001 \| 0 \| 5873 \| \| GO_EXTERNAL_SIDE_OF_PLASMA_MEMBRANE \| 384 \| 0.73249924 \| 2.6070578 \| <0.001 \| <0.001 \| 0 \| 4083 \| \| GO_LEUKOCYTE_HOMEOSTASIS \| 85 \| 0.6989049 \| 2.6035092 \| <0.001 \| <0.001 \| 0 \| 5254 \| \| GO_CYTOKINE_SECRETION \| 240 \| 0.68200344 \| 2.6010406 \| <0.001 \| <0.001 \| 0 \| 3906 \| \| GO_NEGATIVE_REGULATION_OF_CYTOKINE_PRODUCTION \| 294 \| 0.6461788 \| 2.6003869 \| <0.001 \| <0.001 \| 0 \| 5323 \| \| GO_REGULATION_OF_T_CELL_MEDIATED_IMMUNITY \| 71 \| 0.7770872 \| 2.6003542 \| <0.001 \| <0.001 \| 0 \| 4363 \| \| GO_ANTIGEN_RECEPTOR_MEDIATED_SIGNALING_PATHWAY \| 303 \| 0.76760316 \| 2.5950067 \| <0.001 \| <0.001 \| 0 \| 4524 \| \| GO_VACUOLAR_MEMBRANE \| 402 \| 0.6104939 \| 2.5908306 \| <0.001 \| <0.001 \| 0 \| 6053 \| \| GO_CELLULAR_RESPONSE_TO_BIOTIC_STIMULUS \| 241 \| 0.67446935 \| 2.5889316 \| <0.001 \| <0.001 \| 0 \| 4504 \| \| GO_NEGATIVE_REGULATION_OF_LYMPHOCYTE_ACTIVATION \| 149 \| 0.70498085 \| 2.5883803 \| <0.001 \| <0.001 \| 0 \| 5604 \| \| GO_LYMPHOCYTE_MEDIATED_IMMUNITY \| 347 \| 0.7864322 \| 2.587145 \| <0.001 \| <0.001 \| 0 \| 3293 \| \| GO_POSITIVE_REGULATION_OF_LEUKOCYTE_MEDIATED_IMMUNITY \| 131 \| 0.70648855 \| 2.5838053 \| <0.001 \| <0.001 \| 0 \| 6064 \| \| GO_POSITIVE_REGULATION_OF_LYMPHOCYTE_DIFFERENTIATION \| 94 \| 0.7589395 \| 2.5798736 \| <0.001 \| <0.001 \| 0 \| 4546 \| \| GO_NEGATIVE_REGULATION_OF_CELL_ACTIVATION \| 201 \| 0.68856937 \| 2.5778835 \| <0.001 \| <0.001 \| 0 \| 4775 \| \| GO_SECRETORY_GRANULE_MEMBRANE \| 296 \| 0.6382415 \| 2.5750897 \| <0.001 \| <0.001 \| 0 \| 7780 \| \| GO_POSITIVE_REGULATION_OF_LYMPHOCYTE_MEDIATED_IMMUNITY \| 103 \| 0.7253227 \| 2.5747852 \| <0.001 \| <0.001 \| 0 \| 6064 \| \| GO_RESPONSE_TO_MOLECULE_OF_BACTERIAL_ORIGIN \| 349 \| 0.6511073 \| 2.5729783 \| <0.001 \| <0.001 \| 0 \| 4504 \| \| GO_POSITIVE_REGULATION_OF_T_CELL_MEDIATED_IMMUNITY \| 50 \| 0.7886274 \| 2.5728874 \| <0.001 \| <0.001 \| 0 \| 6064 \| \| GO_ADAPTIVE_IMMUNE_RESPONSE_BASED_ON_SOMATIC_RECOMBINATION_OF_IMMUNE_RECEPTORS_BUILT_FROM_IMMUNOGLOBULIN_SUPERFAMILY_DOMAINS \| 347 \| 0.79318887 \| 2.5721066 \| <0.001 \| <0.001 \| 0 \| 4425 \| \| GO_RESPONSE_TO_INTERLEUKIN_12 \| 50 \| 0.6405748 \| 2.5702484 \| <0.001 \| <0.001 \| 0 \| 10574 \| \| GO_POSITIVE_REGULATION_OF_T_CELL_PROLIFERATION \| 95 \| 0.80504984 \| 2.569728 \| <0.001 \| <0.001 \| 0 \| 4546 \| \| GO_LEUKOCYTE_MEDIATED_CYTOTOXICITY \| 106 \| 0.7479141 \| 2.568185 \| <0.001 \| <0.001 \| 0.001 \| 4238 \| \| GO_POSITIVE_REGULATION_OF_LEUKOCYTE_DIFFERENTIATION \| 147 \| 0.71578276 \| 2.5653405 \| <0.001 \| <0.001 \| 0.002 \| 5317 \| \| GO_VACUOLAR_LUMEN \| 170 \| 0.62345403 \| 2.5638344 \| <0.001 \| <0.001 \| 0.002 \| 5052 \| \| GO_REGULATION_OF_CYTOKINE_PRODUCTION_INVOLVED_IN_IMMUNE_RESPONSE \| 85 \| 0.76103956 \| 2.5636988 \| <0.001 \| <0.001 \| 0.002 \| 4387 \| \| GO_INTERFERON_GAMMA_PRODUCTION \| 110 \| 0.7827198 \| 2.5634558 \| <0.001 \| <0.001 \| 0.002 \| 4379 \| \| GO_NEGATIVE_REGULATION_OF_LEUKOCYTE_PROLIFERATION \| 79 \| 0.7616895 \| 2.561199 \| <0.001 \| <0.001 \| 0.003 \| 2886 \| \| GO_INTERLEUKIN_8_PRODUCTION \| 82 \| 0.7525492 \| 2.556571 \| <0.001 \| <0.001 \| 0.003 \| 4006 \| \| GO_RESPONSE_TO_TYPE_I_INTERFERON \| 95 \| 0.7700989 \| 2.555576 \| <0.001 \| <0.001 \| 0.003 \| 2856 \| \| GO_REGULATION_OF_LYMPHOCYTE_APOPTOTIC_PROCESS \| 55 \| 0.7621722 \| 2.5537517 \| <0.001 \| <0.001 \| 0.003 \| 3943 \| \| GO_POSITIVE_REGULATION_OF_HEMOPOIESIS \| 187 \| 0.6583879 \| 2.548549 \| <0.001 \| <0.001 \| 0.005 \| 5317 \| \| GO_REGULATION_OF_LEUKOCYTE_APOPTOTIC_PROCESS \| 85 \| 0.74095124 \| 2.5475776 \| <0.001 \| <0.001 \| 0.005 \| 4202 \| \| GO_ALPHA_BETA_T_CELL_ACTIVATION \| 136 \| 0.71553844 \| 2.5451508 \| <0.001 \| <0.001 \| 0.005 \| 5317 \| \| GO_INTERLEUKIN_6_PRODUCTION \| 162 \| 0.71044034 \| 2.542204 \| <0.001 \| <0.001 \| 0.005 \| 4486 \| \| GO_T_CELL_MEDIATED_CYTOTOXICITY \| 42 \| 0.8148722 \| 2.5417624 \| <0.001 \| <0.001 \| 0.005 \| 6064 \| \| GO_I_KAPPAB_KINASE_NF_KAPPAB_SIGNALING \| 270 \| 0.6242109 \| 2.5395916 \| <0.001 \| <0.001 \| 0.005 \| 6021 \| \| GO_B_CELL_ACTIVATION \| 306 \| 0.7015154 \| 2.5391643 \| <0.001 \| <0.001 \| 0.005 \| 4425 \| \| GO_NEGATIVE_REGULATION_OF_DEFENSE_RESPONSE \| 234 \| 0.5906757 \| 2.5344548 \| <0.001 \| <0.001 \| 0.006 \| 5748 \| \| GO_REGULATION_OF_SYMBIOSIS_ENCOMPASSING_MUTUALISM_THROUGH_PARASITISM \| 221 \| 0.5827631 \| 2.532401 \| <0.001 \| <0.001 \| 0.006 \| 7644 \| \| GO_POSITIVE_REGULATION_OF_PRODUCTION_OF_MOLECULAR_MEDIATOR_OF_IMMUNE_RESPONSE \| 96 \| 0.6575864 \| 2.5312836 \| <0.001 \| <0.001 \| 0.006 \| 3418 \| \| GO_VIRAL_GENOME_REPLICATION \| 122 \| 0.66456485 \| 2.5302794 \| <0.001 \| <0.001 \| 0.006 \| 5871 \| \| GO_SUPEROXIDE_METABOLIC_PROCESS \| 71 \| 0.6933483 \| 2.5288148 \| <0.001 \| <0.001 \| 0.006 \| 5488 \| \| GO_ENDOCYTIC_VESICLE_MEMBRANE \| 158 \| 0.654198 \| 2.525036 \| <0.001 \| <0.001 \| 0.006 \| 5870 \| \| GO_LYMPHOCYTE_HOMEOSTASIS \| 61 \| 0.69167084 \| 2.5245824 \| <0.001 \| <0.001 \| 0.006 \| 5254 \| \| GO_POSITIVE_REGULATION_OF_I_KAPPAB_KINASE_NF_KAPPAB_SIGNALING \| 184 \| 0.6320742 \| 2.5239105 \| <0.001 \| <0.001 \| 0.006 \| 7633 \| \| GO_POSITIVE_REGULATION_OF_PEPTIDE_SECRETION \| 290 \| 0.57247174 \| 2.5231116 \| <0.001 \| <0.001 \| 0.006 \| 5317 \| |

GSEA, gene set enrichment analysis; ES, Enrichment score; NES, Normalized enrichment score; NOM p-val, Nominal p value; FDR q-val, False discovery rate q-value; FWER p-val, Familywise-error rate p-value.
