# Supplementary material for: Establishment of immune prognostic signature and analysis of prospective molecular mechanisms in childhood osteosarcoma patients
Source: Medicine (Baltimore). 2020 Nov 13;99(46):e23251. doi: 10.1097/MD.0000000000023251 (PMC7668544; doi:10.1097/MD.0000000000023251)
Supplement: Supplemental Digital Content [file medi-99-e23251-s009.docx]

| Table S3. Part of the results of GSEA analysis in the high expression group of CCL8. |
| --- |
| \| GS<br> follow link to MSigDB \| SIZE \| ES \| NES \| NOM  p-val \| FDR  q-val \| FWER  p-val \| RANK AT MAX \|  \|  \| \| --- \| --- \| --- \| --- \| --- \| --- \| --- \| --- \| --- \| --- \| |
| \| MIKKELSEN_MCV6_LCP_WITH_H3K4ME3 \| 162 \| 0.680289 \| 2.738547 \| <0.001 \| <0.001 \| 0 \| 4166 \| \| --- \| --- \| --- \| --- \| --- \| --- \| --- \| --- \| \| KEGG_PRION_DISEASES \| 35 \| 0.753840 \| 2.735265 \| <0.001 \| <0.001 \| 0 \| 3163 \| \| KEGG_CELL_ADHESION_MOLECULES_CAMS \| 131 \| 0.766939 \| 2.644151 \| <0.001 \| <0.001 \| 0.001 \| 3508 \| \| REACTOME_SIGNALING_BY_INTERLEUKINS \| 448 \| 0.609327 \| 2.637190 \| <0.001 \| <0.001 \| 0.001 \| 4951 \| \| REACTOME_NEUTROPHIL_DEGRANULATION \| 476 \| 0.672624 \| 2.636189 \| <0.001 \| <0.001 \| 0.001 \| 4771 \| \| KEGG_CYTOKINE_CYTOKINE_RECEPTOR_INTERACTION \| 264 \| 0.710527 \| 2.616738 \| <0.001 \| <0.001 \| 0.002 \| 5097 \| \| WANG_ESOPHAGUS_CANCER_VS_NORMAL_UP \| 119 \| 0.688885 \| 2.609480 \| <0.001 \| <0.001 \| 0.002 \| 7149 \| \| DIAZ_CHRONIC_MEYLOGENOUS_LEUKEMIA_DN \| 116 \| 0.748934 \| 2.605069 \| <0.001 \| <0.001 \| 0.003 \| 3696 \| \| SANSOM_APC_TARGETS_DN \| 338 \| 0.645575 \| 2.604976 \| <0.001 \| <0.001 \| 0.003 \| 5039 \| \| GAL_LEUKEMIC_STEM_CELL_DN \| 233 \| 0.706739 \| 2.602076 \| <0.001 \| <0.001 \| 0.003 \| 2064 \| \| REACTOME_INTERFERON_SIGNALING \| 199 \| 0.708703 \| 2.582684 \| <0.001 \| <0.001 \| 0.004 \| 3367 \| \| TARTE_PLASMA_CELL_VS_PLASMABLAST_UP \| 393 \| 0.572080 \| 2.570314 \| <0.001 \| <0.001 \| 0.006 \| 10896 \| \| FOSTER_TOLERANT_MACROPHAGE_UP \| 165 \| 0.643773 \| 2.569093 \| <0.001 \| <0.001 \| 0.006 \| 4482 \| \| KEGG_CHEMOKINE_SIGNALING_PATHWAY \| 188 \| 0.678019 \| 2.567527 \| <0.001 \| <0.001 \| 0.006 \| 4306 \| \| BOYLAN_MULTIPLE_MYELOMA_PCA1_UP \| 120 \| 0.721142 \| 2.563863 \| <0.001 \| <0.001 \| 0.006 \| 2743 \| \| MUNSHI_MULTIPLE_MYELOMA_UP \| 77 \| 0.629801 \| 2.555950 \| <0.001 \| <0.001 \| 0.006 \| 7635 \| \| REACTOME_DETOXIFICATION_OF_REACTIVE_OXYGEN_SPECIES \| 37 \| 0.724462 \| 2.551042 \| <0.001 \| <0.001 \| 0.007 \| 4671 \| \| WORSCHECH_TUMOR_EVASION_AND_TOLEROGENICITY_UP \| 32 \| 0.835198 \| 2.548121 \| <0.001 \| <0.001 \| 0.007 \| 2743 \| \| WORSCHECH_TUMOR_REJECTION_UP \| 60 \| 0.831399 \| 2.547396 \| <0.001 \| <0.001 \| 0.007 \| 1871 \| \| BOYLAN_MULTIPLE_MYELOMA_C_D_DN \| 277 \| 0.660629 \| 2.542338 \| <0.001 \| <0.001 \| 0.007 \| 2866 \| \| XU_CREBBP_TARGETS_DN \| 40 \| 0.667108 \| 2.540997 \| <0.001 \| <0.001 \| 0.007 \| 5316 \| \| JAATINEN_HEMATOPOIETIC_STEM_CELL_DN \| 234 \| 0.754154 \| 2.528236 \| <0.001 \| <0.001 \| 0.008 \| 4186 \| \| QI_PLASMACYTOMA_UP \| 254 \| 0.765484 \| 2.527363 \| <0.001 \| <0.001 \| 0.008 \| 3367 \| \| ZHENG_IL22_SIGNALING_UP \| 58 \| 0.755827 \| 2.526752 \| <0.001 \| <0.001 \| 0.008 \| 5489 \| \| KEGG_NATURAL_KILLER_CELL_MEDIATED_CYTOTOXICITY \| 132 \| 0.676974 \| 2.526346 \| <0.001 \| <0.001 \| 0.008 \| 4186 \| \| VERHAAK_AML_WITH_NPM1_MUTATED_UP \| 178 \| 0.789137 \| 2.524203 \| <0.001 \| <0.001 \| 0.008 \| 2936 \| \| REACTOME_INTERLEUKIN_3_INTERLEUKIN_5_AND_GM_CSF_SIGNALING \| 48 \| 0.742326 \| 2.523435 \| <0.001 \| <0.001 \| 0.008 \| 3977 \| \| PID_TXA2PATHWAY \| 56 \| 0.765393 \| 2.510881 \| <0.001 \| <0.001 \| 0.01 \| 2401 \| \| FONTAINE_FOLLICULAR_THYROID_ADENOMA_DN \| 69 \| 0.626586 \| 2.509612 \| <0.001 \| <0.001 \| 0.01 \| 5465 \| \| CHYLA_CBFA2T3_TARGETS_UP \| 376 \| 0.620650 \| 2.509445 \| <0.001 \| <0.001 \| 0.01 \| 7445 \| \| REACTOME_COSTIMULATION_BY_THE_CD28_FAMILY \| 72 \| 0.819643 \| 2.503284 \| <0.001 \| <0.001 \| 0.01 \| 1703 \| \| MIKKELSEN_IPS_LCP_WITH_H3K4ME3 \| 172 \| 0.614822 \| 2.502364 \| <0.001 \| <0.001 \| 0.01 \| 4543 \| \| LIU_SMARCA4_TARGETS \| 51 \| 0.720753 \| 2.498561 \| <0.001 \| <0.001 \| 0.01 \| 4423 \| \| KUMAR_TARGETS_OF_MLL_AF9_FUSION \| 401 \| 0.561294 \| 2.498128 \| <0.001 \| <0.001 \| 0.01 \| 8710 \| \| KEGG_LEUKOCYTE_TRANSENDOTHELIAL_MIGRATION \| 116 \| 0.666455 \| 2.494037 \| <0.001 \| <0.001 \| 0.011 \| 2876 \| \| RODWELL_AGING_KIDNEY_UP \| 492 \| 0.713644 \| 2.493855 \| <0.001 \| <0.001 \| 0.011 \| 5329 \| \| LI_INDUCED_T_TO_NATURAL_KILLER_UP \| 313 \| 0.667713 \| 2.490870 \| <0.001 \| <0.001 \| 0.011 \| 4477 \| \| HELLER_SILENCED_BY_METHYLATION_UP \| 277 \| 0.693015 \| 2.490239 \| <0.001 \| <0.001 \| 0.011 \| 5188 \| \| KEGG_ANTIGEN_PROCESSING_AND_PRESENTATION \| 81 \| 0.775315 \| 2.488970 \| <0.001 \| <0.001 \| 0.011 \| 3359 \| \| KEGG_CYTOSOLIC_DNA_SENSING_PATHWAY \| 54 \| 0.709446 \| 2.482078 \| <0.001 \| <0.001 \| 0.012 \| 3788 \| \| ZHAN_MULTIPLE_MYELOMA_MF_UP \| 48 \| 0.718988 \| 2.476498 \| <0.001 \| <0.001 \| 0.012 \| 4944 \| \| REACTOME_CLASS_A_1_RHODOPSIN_LIKE_RECEPTORS \| 329 \| 0.600442 \| 2.476305 \| <0.001 \| <0.001 \| 0.012 \| 6023 \| \| MULLIGHAN_MLL_SIGNATURE_1_UP \| 381 \| 0.644864 \| 2.474678 \| <0.001 \| <0.001 \| 0.012 \| 6661 \| \| TAKEDA_TARGETS_OF_NUP98_HOXA9_FUSION_10D_DN \| 150 \| 0.690095 \| 2.474591 \| <0.001 \| <0.001 \| 0.012 \| 5838 \| \| MARTENS_BOUND_BY_PML_RARA_FUSION \| 445 \| 0.590798 \| 2.472791 \| <0.001 \| <0.001 \| 0.013 \| 6885 \| \| KEGG_TOLL_LIKE_RECEPTOR_SIGNALING_PATHWAY \| 102 \| 0.698737 \| 2.468827 \| <0.001 \| <0.001 \| 0.013 \| 7806 \| \| ACOSTA_PROLIFERATION_INDEPENDENT_MYC_TARGETS_DN \| 116 \| 0.637254 \| 2.466751 \| <0.001 \| <0.001 \| 0.014 \| 5137 \| \| RASHI_RESPONSE_TO_IONIZING_RADIATION_6 \| 83 \| 0.766424 \| 2.465236 \| <0.001 \| <0.001 \| 0.015 \| 3367 \| \| ROSS_AML_WITH_MLL_FUSIONS \| 80 \| 0.709584 \| 2.464798 \| <0.001 \| <0.001 \| 0.016 \| 5148 \| \| RUTELLA_RESPONSE_TO_HGF_DN \| 233 \| 0.659500 \| 2.463423 \| <0.001 \| <0.001 \| 0.016 \| 4735 \| \| FURUKAWA_DUSP6_TARGETS_PCI35_UP \| 71 \| 0.727796 \| 2.461883 \| <0.001 \| <0.001 \| 0.016 \| 6574 \| \| LEE_AGING_CEREBELLUM_UP \| 79 \| 0.682320 \| 2.460830 \| <0.001 \| <0.001 \| 0.017 \| 6559 \| \| HOFFMANN_SMALL_PRE_BII_TO_IMMATURE_B_LYMPHOCYTE_UP \| 65 \| 0.749354 \| 2.457225 \| <0.001 \| <0.001 \| 0.017 \| 5921 \| \| TAKEDA_TARGETS_OF_NUP98_HOXA9_FUSION_16D_UP \| 176 \| 0.656476 \| 2.456799 \| <0.001 \| <0.001 \| 0.017 \| 3656 \| \| BOYLAN_MULTIPLE_MYELOMA_D_DN \| 78 \| 0.673196 \| 2.456098 \| <0.001 \| <0.001 \| 0.017 \| 2629 \| \| TAKEDA_TARGETS_OF_NUP98_HOXA9_FUSION_8D_DN \| 196 \| 0.676821 \| 2.453708 \| <0.001 \| <0.001 \| 0.017 \| 3461 \| \| LEE_DIFFERENTIATING_T_LYMPHOCYTE \| 196 \| 0.749426 \| 2.452371 \| <0.001 \| <0.001 \| 0.017 \| 4412 \| \| GRAESSMANN_RESPONSE_TO_MC_AND_SERUM_DEPRIVATION_UP \| 211 \| 0.651691 \| 2.447958 \| <0.001 \| 0.0010088 \| 0.018 \| 4189 \| \| ICHIBA_GRAFT_VERSUS_HOST_DISEASE_35D_UP \| 143 \| 0.822712 \| 2.441215 \| <0.001 \| 0.00108084 \| 0.018 \| 2444 \| \| KEGG_PATHOGENIC_ESCHERICHIA_COLI_INFECTION \| 56 \| 0.576618 \| 2.441162 \| <0.001 \| 0.00106282 \| 0.018 \| 5060 \| \| TONKS_TARGETS_OF_RUNX1_RUNX1T1_FUSION_HSC_DN \| 193 \| 0.698505 \| 2.440033 \| <0.001 \| 0.00109409 \| 0.019 \| 4932 \| \| RUTELLA_RESPONSE_TO_CSF2RB_AND_IL4_DN \| 315 \| 0.711612 \| 2.439823 \| <0.001 \| 0.00107644 \| 0.019 \| 4235 \| \| BOSCO_TH1_CYTOTOXIC_MODULE \| 113 \| 0.777258 \| 2.439160 \| <0.001 \| 0.00107325 \| 0.019 \| 2977 \| \| PID_IL27_PATHWAY \| 26 \| 0.846340 \| 2.438550 \| <0.001 \| 0.00105648 \| 0.019 \| 3636 \| \| SMID_BREAST_CANCER_NORMAL_LIKE_UP \| 481 \| 0.729081 \| 2.436644 \| <0.001 \| 0.00109049 \| 0.02 \| 5430 \| \| REACTOME_PEPTIDE_LIGAND_BINDING_RECEPTORS \| 197 \| 0.612747 \| 2.436626 \| <0.001 \| 0.00108812 \| 0.02 \| 6023 \| \| BASSO_CD40_SIGNALING_DN \| 70 \| 0.666287 \| 2.433889 \| <0.001 \| 0.00113839 \| 0.022 \| 4423 \| \| KEGG_HEMATOPOIETIC_CELL_LINEAGE \| 85 \| 0.769216 \| 2.433470 \| <0.001 \| 0.00112165 \| 0.022 \| 2663 \| \| REACTOME_INTERLEUKIN_2_FAMILY_SIGNALING \| 44 \| 0.798932 \| 2.427813 \| <0.001 \| 0.00130804 \| 0.027 \| 2743 \| \| PID_IL12_2PATHWAY \| 63 \| 0.811235 \| 2.422910 \| <0.001 \| 0.00133793 \| 0.028 \| 2977 \| \| WALLACE_PROSTATE_CANCER_RACE_UP \| 299 \| 0.819715 \| 2.422071 \| <0.001 \| 0.00131908 \| 0.028 \| 2846 \| \| WANG_BARRETTS_ESOPHAGUS_AND_ESOPHAGUS_CANCER_UP \| 26 \| 0.722372 \| 2.421031 \| <0.001 \| 0.00131468 \| 0.028 \| 5692 \| \| JOHNSTONE_PARVB_TARGETS_2_UP \| 142 \| 0.623354 \| 2.416472 \| <0.001 \| 0.00136577 \| 0.028 \| 6796 \| \| FULCHER_INFLAMMATORY_RESPONSE_LECTIN_VS_LPS_DN \| 442 \| 0.717551 \| 2.414189 \| <0.001 \| 0.00139039 \| 0.028 \| 5291 \| \| SATO_SILENCED_BY_METHYLATION_IN_PANCREATIC_CANCER_1 \| 417 \| 0.527738 \| 2.410861 \| <0.001 \| 0.00148737 \| 0.029 \| 8090 \| \| KEGG_T_CELL_RECEPTOR_SIGNALING_PATHWAY \| 108 \| 0.661476 \| 2.406867 \| <0.001 \| 0.0015334 \| 0.03 \| 4396 \| \| DUNNE_TARGETS_OF_AML1_MTG8_FUSION_UP \| 52 \| 0.807899 \| 2.405838 \| <0.001 \| 0.00154587 \| 0.03 \| 2950 \| \| KLEIN_TARGETS_OF_BCR_ABL1_FUSION \| 42 \| 0.803229 \| 2.405461 \| <0.001 \| 0.00152605 \| 0.03 \| 3977 \| \| REACTOME_TNFR2_NON_CANONICAL_NF_KB_PATHWAY \| 101 \| 0.652665 \| 2.402550 \| <0.001 \| 0.00160986 \| 0.031 \| 5112 \| \| MISSIAGLIA_REGULATED_BY_METHYLATION_UP \| 122 \| 0.688355 \| 2.400825 \| <0.001 \| 0.00162352 \| 0.032 \| 6743 \| \| PID_PTP1B_PATHWAY \| 52 \| 0.685786 \| 2.399267 \| <0.001 \| 0.00164151 \| 0.032 \| 2545 \| \| ONO_AML1_TARGETS_DN \| 40 \| 0.763461 \| 2.398040 \| <0.001 \| 0.00162149 \| 0.032 \| 4660 \| \| PEPPER_CHRONIC_LYMPHOCYTIC_LEUKEMIA_UP \| 31 \| 0.754120 \| 2.397349 \| <0.001 \| 0.00162861 \| 0.032 \| 1870 \| \| PID_TCR_PATHWAY \| 65 \| 0.777710 \| 2.396266 \| <0.001 \| 0.00160922 \| 0.032 \| 2570 \| \| BYSTROEM_CORRELATED_WITH_IL5_UP \| 38 \| 0.597322 \| 2.394417 \| <0.001 \| 0.00165062 \| 0.033 \| 11912 \| \| REACTOME_TCR_SIGNALING \| 122 \| 0.691771 \| 2.390917 \| <0.001 \| 0.00171337 \| 0.033 \| 1470 \| \| KATSANOU_ELAVL1_TARGETS_UP \| 157 \| 0.621408 \| 2.389726 \| <0.001 \| 0.00170864 \| 0.034 \| 6650 \| \| DELYS_THYROID_CANCER_UP \| 436 \| 0.598819 \| 2.387357 \| <0.001 \| 0.00178068 \| 0.037 \| 6172 \| \| REACTOME_INTERLEUKIN_RECEPTOR_SHC_SIGNALING \| 27 \| 0.776369 \| 2.386349 \| <0.001 \| 0.00183039 \| 0.037 \| 2427 \| \| SCHLOSSER_SERUM_RESPONSE_UP \| 126 \| 0.562722 \| 2.385965 \| <0.001 \| 0.00181005 \| 0.037 \| 4680 \| \| WUNDER_INFLAMMATORY_RESPONSE_AND_CHOLESTEROL_UP \| 60 \| 0.848918 \| 2.385801 \| <0.001 \| 0.00179016 \| 0.037 \| 3595 \| \| KEGG_NOD_LIKE_RECEPTOR_SIGNALING_PATHWAY \| 62 \| 0.762868 \| 2.385752 \| <0.001 \| 0.0017707 \| 0.037 \| 3879 \| \| MARKEY_RB1_CHRONIC_LOF_DN \| 117 \| 0.742817 \| 2.385622 \| <0.001 \| 0.00175166 \| 0.037 \| 3586 \| \| VART_KSHV_INFECTION_ANGIOGENIC_MARKERS_UP \| 161 \| 0.647137 \| 2.385294 \| <0.001 \| 0.00177052 \| 0.037 \| 6483 \| \| HUTTMANN_B_CLL_POOR_SURVIVAL_UP \| 277 \| 0.551026 \| 2.384881 \| <0.001 \| 0.00176545 \| 0.037 \| 7026 \| \| RAMALHO_STEMNESS_DN \| 72 \| 0.735222 \| 2.383969 \| <0.001 \| 0.00176219 \| 0.038 \| 6186 \| \| MULLIGHAN_MLL_SIGNATURE_2_UP \| 419 \| 0.626577 \| 2.382165 \| <0.001 \| 0.00177662 \| 0.039 \| 7199 \| \| FERRANDO_T_ALL_WITH_MLL_ENL_FUSION_UP \| 89 \| 0.641861 \| 2.381822 \| <0.001 \| 0.00175849 \| 0.039 \| 4562 \| \| NABA_SECRETED_FACTORS \| 342 \| 0.563004 \| 2.381225 \| <0.001 \| 0.00174072 \| 0.039 \| 10213 \| \| MIKKELSEN_ES_LCP_WITH_H3K4ME3 \| 143 \| 0.593839 \| 2.380234 \| <0.001 \| 0.00174561 \| 0.04 \| 4429 \| \| GO_POSITIVE_REGULATION_OF_CYTOKINE_PRODUCTION \| 461 \| 0.678704 \| 2.762394 \| <0.001 \| <0.001 \| 0.001 \| 4484 \| \| GO_NEGATIVE_REGULATION_OF_MULTI_ORGANISM_PROCESS \| 222 \| 0.686357 \| 2.744848 \| <0.001 \| <0.001 \| 0.001 \| 6196 \| \| GO_REGULATION_OF_PRODUCTION_OF_MOLECULAR_MEDIATOR_OF_IMMUNE_RESPONSE \| 141 \| 0.711370 \| 2.740395 \| <0.001 \| <0.001 \| 0.001 \| 3690 \| \| GO_LEUKOCYTE_PROLIFERATION \| 299 \| 0.717269 \| 2.726456 \| <0.001 \| <0.001 \| 0.001 \| 2883 \| \| GO_NEGATIVE_REGULATION_OF_IMMUNE_RESPONSE \| 152 \| 0.709958 \| 2.707092 \| <0.001 \| <0.001 \| 0.001 \| 2992 \| \| GO_NEGATIVE_REGULATION_OF_CYTOKINE_PRODUCTION \| 294 \| 0.655581 \| 2.702826 \| <0.001 \| <0.001 \| 0.001 \| 5042 \| \| GO_LEUKOCYTE_APOPTOTIC_PROCESS \| 107 \| 0.720601 \| 2.687472 \| <0.001 \| <0.001 \| 0.001 \| 2725 \| \| GO_RESPONSE_TO_MOLECULE_OF_BACTERIAL_ORIGIN \| 349 \| 0.674549 \| 2.677630 \| <0.001 \| <0.001 \| 0.001 \| 5115 \| \| GO_REGULATION_OF_ADAPTIVE_IMMUNE_RESPONSE \| 159 \| 0.744935 \| 2.677430 \| <0.001 \| <0.001 \| 0.001 \| 2866 \| \| GO_REGULATION_OF_CELL_CELL_ADHESION \| 406 \| 0.672360 \| 2.672797 \| <0.001 \| <0.001 \| 0.001 \| 4407 \| \| GO_T_CELL_PROLIFERATION \| 185 \| 0.742606 \| 2.667211 \| <0.001 \| <0.001 \| 0.001 \| 2257 \| \| GO_CELLULAR_RESPONSE_TO_BIOTIC_STIMULUS \| 241 \| 0.701819 \| 2.664008 \| <0.001 \| <0.001 \| 0.001 \| 4484 \| \| GO_REGULATION_OF_LEUKOCYTE_MEDIATED_IMMUNITY \| 198 \| 0.747979 \| 2.659438 \| <0.001 \| <0.001 \| 0.001 \| 5229 \| \| GO_LYMPHOCYTE_DIFFERENTIATION \| 353 \| 0.665705 \| 2.659196 \| <0.001 \| <0.001 \| 0.001 \| 2977 \| \| GO_POSITIVE_REGULATION_OF_RESPONSE_TO_BIOTIC_STIMULUS \| 396 \| 0.611133 \| 2.658177 \| <0.001 \| <0.001 \| 0.001 \| 4505 \| \| GO_T_CELL_ACTIVATION \| 466 \| 0.689401 \| 2.655594 \| <0.001 \| <0.001 \| 0.001 \| 3461 \| \| GO_RESPONSE_TO_VIRUS \| 324 \| 0.660112 \| 2.655445 \| <0.001 \| <0.001 \| 0.001 \| 4190 \| \| GO_TUMOR_NECROSIS_FACTOR_SUPERFAMILY_CYTOKINE_PRODUCTION \| 170 \| 0.719630 \| 2.653327 \| <0.001 \| <0.001 \| 0.001 \| 4396 \| \| GO_INTERLEUKIN_6_PRODUCTION \| 162 \| 0.727615 \| 2.646240 \| <0.001 \| <0.001 \| 0.001 \| 6240 \| \| GO_CELL_CHEMOTAXIS \| 297 \| 0.703142 \| 2.646199 \| <0.001 \| <0.001 \| 0.001 \| 4482 \| \| GO_MYELOID_LEUKOCYTE_MIGRATION \| 204 \| 0.728431 \| 2.644404 \| <0.001 \| <0.001 \| 0.001 \| 4482 \| \| GO_NEGATIVE_REGULATION_OF_RESPONSE_TO_BIOTIC_STIMULUS \| 95 \| 0.714809 \| 2.643883 \| <0.001 \| <0.001 \| 0.001 \| 7427 \| \| GO_POSITIVE_REGULATION_OF_PEPTIDE_SECRETION \| 290 \| 0.610527 \| 2.643065 \| <0.001 \| <0.001 \| 0.001 \| 5470 \| \| GO_VESICLE_LUMEN \| 326 \| 0.600786 \| 2.640681 \| <0.001 \| <0.001 \| 0.001 \| 6347 \| \| GO_MODIFICATION_OF_MORPHOLOGY_OR_PHYSIOLOGY_OF_OTHER_ORGANISM \| 164 \| 0.566812 \| 2.639759 \| <0.001 \| <0.001 \| 0.001 \| 4951 \| \| GO_LEUKOCYTE_CELL_CELL_ADHESION \| 338 \| 0.715037 \| 2.628171 \| <0.001 \| <0.001 \| 0.001 \| 4407 \| \| GO_CYTOKINE_RECEPTOR_BINDING \| 281 \| 0.635852 \| 2.628037 \| <0.001 \| <0.001 \| 0.001 \| 7882 \| \| GO_POSITIVE_REGULATION_OF_IMMUNE_EFFECTOR_PROCESS \| 211 \| 0.700907 \| 2.621395 \| <0.001 \| <0.001 \| 0.001 \| 5229 \| \| GO_NEGATIVE_REGULATION_OF_LYMPHOCYTE_ACTIVATION \| 149 \| 0.708202 \| 2.620211 \| <0.001 \| <0.001 \| 0.001 \| 3213 \| \| GO_ACTIVATION_OF_INNATE_IMMUNE_RESPONSE \| 318 \| 0.600104 \| 2.619798 \| <0.001 \| <0.001 \| 0.001 \| 4505 \| \| GO_CYTOKINE_SECRETION \| 240 \| 0.693224 \| 2.618793 \| <0.001 \| <0.001 \| 0.001 \| 5838 \| \| GO_CELL_KILLING \| 166 \| 0.709027 \| 2.618642 \| <0.001 \| <0.001 \| 0.001 \| 5429 \| \| GO_REGULATION_OF_LEUKOCYTE_PROLIFERATION \| 223 \| 0.730667 \| 2.613969 \| <0.001 \| <0.001 \| 0.001 \| 4482 \| \| GO_REGULATION_OF_LYMPHOCYTE_MEDIATED_IMMUNITY \| 147 \| 0.733966 \| 2.613326 \| <0.001 \| <0.001 \| 0.001 \| 2866 \| \| GO_NEGATIVE_REGULATION_OF_LEUKOCYTE_PROLIFERATION \| 79 \| 0.762400 \| 2.612931 \| <0.001 \| <0.001 \| 0.001 \| 1517 \| \| GO_REGULATION_OF_LEUKOCYTE_APOPTOTIC_PROCESS \| 85 \| 0.747318 \| 2.610004 \| <0.001 \| <0.001 \| 0.001 \| 3768 \| \| GO_LEUKOCYTE_CHEMOTAXIS \| 218 \| 0.741383 \| 2.608187 \| <0.001 \| <0.001 \| 0.001 \| 4482 \| \| GO_POSITIVE_REGULATION_OF_INTERLEUKIN_6_PRODUCTION \| 95 \| 0.757035 \| 2.605420 \| <0.001 \| <0.001 \| 0.001 \| 4698 \| \| GO_NEGATIVE_REGULATION_OF_IMMUNE_EFFECTOR_PROCESS \| 120 \| 0.688085 \| 2.604640 \| <0.001 \| <0.001 \| 0.001 \| 2866 \| \| GO_POSITIVE_REGULATION_OF_CELL_CELL_ADHESION \| 256 \| 0.733980 \| 2.603018 \| <0.001 \| <0.001 \| 0.001 \| 3459 \| \| GO_LYMPHOCYTE_ACTIVATION_INVOLVED_IN_IMMUNE_RESPONSE \| 181 \| 0.681841 \| 2.602718 \| <0.001 \| <0.001 \| 0.001 \| 3679 \| \| GO_NEGATIVE_REGULATION_OF_IMMUNE_SYSTEM_PROCESS \| 452 \| 0.624725 \| 2.602209 \| <0.001 \| <0.001 \| 0.001 \| 7511 \| \| GO_CYTOKINE_METABOLIC_PROCESS \| 123 \| 0.735944 \| 2.599254 \| <0.001 \| <0.001 \| 0.001 \| 4484 \| \| GO_T_CELL_APOPTOTIC_PROCESS \| 49 \| 0.729925 \| 2.598561 \| <0.001 \| <0.001 \| 0.001 \| 2051 \| \| GO_NEGATIVE_REGULATION_OF_CELL_ACTIVATION \| 201 \| 0.687816 \| 2.597408 \| <0.001 \| <0.001 \| 0.001 \| 4407 \| \| GO_GRANULOCYTE_MIGRATION \| 138 \| 0.759060 \| 2.596084 \| <0.001 \| <0.001 \| 0.001 \| 4482 \| \| GO_RESPONSE_TO_INTERFERON_GAMMA \| 193 \| 0.786602 \| 2.590838 \| <0.001 \| <0.001 \| 0.001 \| 3452 \| \| GO_REGULATION_OF_PHAGOCYTOSIS \| 93 \| 0.749078 \| 2.586610 \| <0.001 \| <0.001 \| 0.001 \| 2554 \| \| GO_POSITIVE_REGULATION_OF_PRODUCTION_OF_MOLECULAR_MEDIATOR_OF_IMMUNE_RESPONSE \| 96 \| 0.684578 \| 2.586198 \| <0.001 \| <0.001 \| 0.001 \| 3690 \| \| GO_REGULATION_OF_T_CELL_ACTIVATION \| 316 \| 0.713318 \| 2.582685 \| <0.001 \| <0.001 \| 0.001 \| 3213 \| \| GO_CYTOKINE_ACTIVITY \| 217 \| 0.668341 \| 2.582578 \| <0.001 \| <0.001 \| 0.001 \| 7896 \| \| GO_POSITIVE_REGULATION_OF_CYTOKINE_SECRETION \| 139 \| 0.718048 \| 2.580864 \| <0.001 \| <0.001 \| 0.001 \| 4698 \| \| GO_SECRETORY_GRANULE_MEMBRANE \| 296 \| 0.656216 \| 2.578039 \| <0.001 \| <0.001 \| 0.001 \| 4736 \| \| GO_SPECIFIC_GRANULE \| 159 \| 0.699484 \| 2.577090 \| <0.001 \| <0.001 \| 0.002 \| 7710 \| \| GO_NEGATIVE_REGULATION_OF_DEFENSE_RESPONSE \| 234 \| 0.598701 \| 2.570539 \| <0.001 \| <0.001 \| 0.002 \| 7427 \| \| GO_REGULATION_OF_LEUKOCYTE_DIFFERENTIATION \| 274 \| 0.670486 \| 2.569812 \| <0.001 \| <0.001 \| 0.002 \| 4396 \| \| GO_NEGATIVE_REGULATION_OF_VIRAL_PROCESS \| 100 \| 0.729977 \| 2.567831 \| <0.001 \| <0.001 \| 0.002 \| 2750 \| \| GO_REGULATION_OF_LYMPHOCYTE_DIFFERENTIATION \| 170 \| 0.694333 \| 2.567673 \| <0.001 \| <0.001 \| 0.002 \| 4396 \| \| GO_INTERLEUKIN_8_PRODUCTION \| 82 \| 0.743701 \| 2.561170 \| <0.001 \| <0.001 \| 0.002 \| 6214 \| \| GO_POSITIVE_REGULATION_OF_LEUKOCYTE_PROLIFERATION \| 139 \| 0.754870 \| 2.560673 \| <0.001 \| <0.001 \| 0.002 \| 4698 \| \| GO_T_CELL_ACTIVATION_INVOLVED_IN_IMMUNE_RESPONSE \| 105 \| 0.722920 \| 2.555144 \| <0.001 \| <0.001 \| 0.002 \| 3213 \| \| GO_REGULATION_OF_HEMOPOIESIS \| 446 \| 0.597183 \| 2.554930 \| <0.001 \| <0.001 \| 0.002 \| 4505 \| \| GO_CYTOKINE_PRODUCTION_INVOLVED_IN_IMMUNE_RESPONSE \| 104 \| 0.724396 \| 2.554473 \| <0.001 \| <0.001 \| 0.002 \| 3690 \| \| GO_REGULATION_OF_LEUKOCYTE_MIGRATION \| 192 \| 0.687000 \| 2.553194 \| <0.001 \| <0.001 \| 0.002 \| 4698 \| \| GO_NEGATIVE_REGULATION_OF_LEUKOCYTE_MEDIATED_IMMUNITY \| 49 \| 0.831968 \| 2.551566 \| <0.001 \| <0.001 \| 0.002 \| 2866 \| \| GO_INTERFERON_GAMMA_PRODUCTION \| 110 \| 0.781042 \| 2.551082 \| <0.001 \| <0.001 \| 0.002 \| 4407 \| \| GO_DEFENSE_RESPONSE_TO_VIRUS \| 241 \| 0.676478 \| 2.549238 \| <0.001 \| <0.001 \| 0.002 \| 4190 \| \| GO_ALPHA_BETA_T_CELL_ACTIVATION \| 136 \| 0.731229 \| 2.546645 \| <0.001 \| <0.001 \| 0.003 \| 2977 \| \| GO_G_PROTEIN_COUPLED_RECEPTOR_BINDING \| 278 \| 0.553349 \| 2.546483 \| <0.001 \| <0.001 \| 0.003 \| 3557 \| \| GO_T_CELL_MEDIATED_IMMUNITY \| 100 \| 0.748323 \| 2.545889 \| <0.001 \| <0.001 \| 0.003 \| 5229 \| \| GO_POSITIVE_REGULATION_OF_SECRETION \| 429 \| 0.567428 \| 2.544751 \| <0.001 \| <0.001 \| 0.003 \| 5868 \| \| GO_T_CELL_DIFFERENTIATION \| 239 \| 0.681825 \| 2.543804 \| <0.001 \| <0.001 \| 0.003 \| 3461 \| \| GO_POSITIVE_REGULATION_OF_REACTIVE_OXYGEN_SPECIES_METABOLIC_PROCESS \| 99 \| 0.639376 \| 2.540770 \| <0.001 \| <0.001 \| 0.003 \| 7965 \| \| GO_LYMPHOCYTE_APOPTOTIC_PROCESS \| 72 \| 0.705890 \| 2.538140 \| <0.001 \| <0.001 \| 0.003 \| 3310 \| \| GO_LEUKOCYTE_MEDIATED_CYTOTOXICITY \| 106 \| 0.766027 \| 2.536664 \| <0.001 \| <0.001 \| 0.005 \| 5229 \| \| GO_T_CELL_RECEPTOR_SIGNALING_PATHWAY \| 199 \| 0.686842 \| 2.536094 \| <0.001 \| <0.001 \| 0.005 \| 3553 \| \| GO_TYPE_I_INTERFERON_PRODUCTION \| 126 \| 0.693112 \| 2.535902 \| <0.001 \| <0.001 \| 0.005 \| 4367 \| \| GO_NEGATIVE_REGULATION_OF_TUMOR_NECROSIS_FACTOR_SUPERFAMILY_CYTOKINE_PRODUCTION \| 75 \| 0.704872 \| 2.535026 \| <0.001 \| <0.001 \| 0.005 \| 2631 \| \| GO_REGULATION_OF_PEPTIDE_SECRETION \| 496 \| 0.555395 \| 2.533986 \| <0.001 \| <0.001 \| 0.005 \| 5537 \| \| GO_REGULATION_OF_CYTOKINE_PRODUCTION_INVOLVED_IN_IMMUNE_RESPONSE \| 85 \| 0.753105 \| 2.533769 \| <0.001 \| <0.001 \| 0.005 \| 3690 \| \| GO_NEUTROPHIL_MIGRATION \| 116 \| 0.773217 \| 2.531634 \| <0.001 \| <0.001 \| 0.006 \| 4482 \| \| GO_NEGATIVE_REGULATION_OF_LYMPHOCYTE_MEDIATED_IMMUNITY \| 39 \| 0.832999 \| 2.531487 \| <0.001 \| <0.001 \| 0.006 \| 2866 \| \| GO_POSITIVE_REGULATION_OF_LEUKOCYTE_CELL_CELL_ADHESION \| 216 \| 0.753818 \| 2.530749 \| <0.001 \| <0.001 \| 0.006 \| 3213 \| \| GO_RESPONSE_TO_TYPE_I_INTERFERON \| 95 \| 0.774308 \| 2.530140 \| <0.001 \| <0.001 \| 0.006 \| 2268 \| \| GO_POSITIVE_REGULATION_OF_CELL_ADHESION \| 397 \| 0.658291 \| 2.527330 \| <0.001 \| <0.001 \| 0.006 \| 4121 \| \| GO_ENTRY_INTO_HOST \| 133 \| 0.625579 \| 2.526998 \| <0.001 \| <0.001 \| 0.006 \| 5489 \| \| GO_INTERLEUKIN_1_PRODUCTION \| 114 \| 0.715630 \| 2.525195 \| <0.001 \| <0.001 \| 0.006 \| 6240 \| \| GO_NEGATIVE_REGULATION_OF_INNATE_IMMUNE_RESPONSE \| 59 \| 0.757661 \| 2.524653 \| <0.001 \| <0.001 \| 0.006 \| 6196 \| \| GO_REGULATION_OF_VIRAL_ENTRY_INTO_HOST_CELL \| 29 \| 0.811916 \| 2.522935 \| <0.001 \| <0.001 \| 0.006 \| 2727 \| \| GO_NEGATIVE_REGULATION_OF_PRODUCTION_OF_MOLECULAR_MEDIATOR_OF_IMMUNE_RESPONSE \| 36 \| 0.791438 \| 2.522878 \| <0.001 \| <0.001 \| 0.006 \| 2866 \| \| GO_POSITIVE_REGULATION_OF_ERK1_AND_ERK2_CASCADE \| 204 \| 0.618639 \| 2.521449 \| <0.001 \| <0.001 \| 0.006 \| 5963 \| \| GO_REGULATION_OF_T_CELL_DIFFERENTIATION \| 139 \| 0.702714 \| 2.519736 \| <0.001 \| <0.001 \| 0.006 \| 4396 \| \| GO_IMMUNE_RECEPTOR_ACTIVITY \| 126 \| 0.763027 \| 2.518870 \| <0.001 \| <0.001 \| 0.006 \| 5646 \| \| GO_REGULATION_OF_INFLAMMATORY_RESPONSE \| 365 \| 0.615675 \| 2.517516 \| <0.001 \| <0.001 \| 0.006 \| 6240 \| \| GO_POSITIVE_REGULATION_OF_LEUKOCYTE_MEDIATED_IMMUNITY \| 131 \| 0.691745 \| 2.516435 \| <0.001 \| <0.001 \| 0.006 \| 5533 \| \| GO_POSITIVE_REGULATION_OF_ADAPTIVE_IMMUNE_RESPONSE \| 104 \| 0.720914 \| 2.513763 \| <0.001 \| <0.001 \| 0.006 \| 5229 \| \| GO_LYMPHOCYTE_MIGRATION \| 112 \| 0.783695 \| 2.512144 \| <0.001 \| <0.001 \| 0.006 \| 4209 \| \| GO_NEGATIVE_REGULATION_OF_LEUKOCYTE_CELL_CELL_ADHESION \| 128 \| 0.676018 \| 2.510207 \| <0.001 \| <0.001 \| 0.007 \| 6837 \| \| GO_NEGATIVE_REGULATION_OF_CELL_CELL_ADHESION \| 181 \| 0.633114 \| 2.509935 \| <0.001 \| <0.001 \| 0.007 \| 4407 \| \| GO_CHEMOKINE_RECEPTOR_BINDING \| 64 \| 0.812950 \| 2.506852 \| <0.001 \| <0.001 \| 0.007 \| 3557 \| |

GSEA, gene set enrichment analysis; ES, Enrichment score; NES, Normalized enrichment score; NOM p-val, Nominal p value; FDR q-val, False discovery rate q-value; FWER p-val, Familywise-error rate p-value.
