# Supplementary material for: Establishment of immune prognostic signature and analysis of prospective molecular mechanisms in childhood osteosarcoma patients
Source: Medicine (Baltimore). 2020 Nov 13;99(46):e23251. doi: 10.1097/MD.0000000000023251 (PMC7668544; doi:10.1097/MD.0000000000023251)
Supplement: Supplemental Digital Content [file medi-99-e23251-s010.docx]

| Table S4. Part of the results of GSEA analysis in the low expression group of CCR4. |
| --- |
| \| GS<br> follow link to MSigDB \| SIZE \| ES \| NES \| NOM  p-val \| FDR  q-val \| FWER  p-val \| RANK AT MAX \| \| --- \| --- \| --- \| --- \| --- \| --- \| --- \| --- \| |
| \| REACTOME_EUKARYOTIC_TRANSLATION_INITIATION \| 120 \| -0.766441 \| -2.177197 \| 0.004008 \| 0.235011 \| 0.181 \| 6436 \| \| --- \| --- \| --- \| --- \| --- \| --- \| --- \| --- \| \| REACTOME_ACTIVATION_OF_THE_MRNA_UPON_BINDING_OF_THE_CAP_BINDING_COMPLEX_AND_EIFS_AND_SUBSEQUENT_BINDING_TO_43S \| 60 \| -0.761013 \| -2.169656 \| 0.004008 \| 0.167860 \| 0.189 \| 5903 \| \| REACTOME_SRP_DEPENDENT_COTRANSLATIONAL_PROTEIN_TARGETING_TO_MEMBRANE \| 113 \| -0.727558 \| -2.080044 \| 0.007737 \| 0.242739 \| 0.308 \| 7356 \| \| REACTOME_EUKARYOTIC_TRANSLATION_ELONGATION \| 94 \| -0.799743 \| -2.073346 \| 0.001938 \| 0.215226 \| 0.318 \| 7356 \| \| REACTOME_RESPONSE_OF_EIF2AK4_GCN2_TO_AMINO_ACID_DEFICIENCY \| 102 \| -0.715540 \| -2.053402 \| 0.005929 \| 0.220191 \| 0.352 \| 7356 \| \| KEGG_RIBOSOME \| 88 \| -0.801695 \| -2.028262 \| 0.003960 \| 0.237578 \| 0.394 \| 7356 \| \| GO_CYTOSOLIC_LARGE_RIBOSOMAL_SUBUNIT \| 57 \| -0.824377 \| -2.090954 \| 0.000000 \| 0.241607 \| 0.261 \| 7356 \| \| GO_COTRANSLATIONAL_PROTEIN_TARGETING_TO_MEMBRANE \| 99 \| -0.757215 \| -2.072089 \| 0.008230 \| 0.216714 \| 0.291 \| 7343 \| \| GO_POLYSOME \| 73 \| -0.555602 \| -2.067765 \| 0.014433 \| 0.180837 \| 0.299 \| 7262 \| |

GSEA, gene set enrichment analysis; ES, Enrichment score; NES, Normalized enrichment score; NOM p-val, Nominal p value; FDR q-val, False discovery rate q-value; FWER p-val, Familywise-error rate p-value.
