# Supplementary material for: Establishment of immune prognostic signature and analysis of prospective molecular mechanisms in childhood osteosarcoma patients
Source: Medicine (Baltimore). 2020 Nov 13;99(46):e23251. doi: 10.1097/MD.0000000000023251 (PMC7668544; doi:10.1097/MD.0000000000023251)
Supplement: Supplemental Digital Content [file medi-99-e23251-s011.docx]

| Table S5. Part of the results of GSEA analysis in the high expression group of CCR5. |
| --- |
| \| GS<br> follow link to MSigDB \| SIZE \| ES \| NES \| NOM  p-val \| FDR  q-val \| FWER  p-val \| RANK AT MAX \| \| --- \| --- \| --- \| --- \| --- \| --- \| --- \| --- \| |
| \| REACTOME_INTERFERON_SIGNALING \| 199 \| 0.735016 \| 2.675485 \| <0.001 \| <0.001 \| 0 \| 4621 \| \| --- \| --- \| --- \| --- \| --- \| --- \| --- \| --- \| \| REACTOME_SIGNALING_BY_INTERLEUKINS \| 448 \| 0.596172 \| 2.628265 \| <0.001 \| <0.001 \| 0 \| 5203 \| \| MIKKELSEN_MCV6_LCP_WITH_H3K4ME3 \| 162 \| 0.638740 \| 2.582371 \| <0.001 \| 0.001792787 \| 0.004 \| 5862 \| \| FOSTER_TOLERANT_MACROPHAGE_UP \| 165 \| 0.644295 \| 2.580025 \| <0.001 \| 0.001589593 \| 0.004 \| 7705 \| \| KEGG_CELL_ADHESION_MOLECULES_CAMS \| 131 \| 0.757806 \| 2.569877 \| <0.001 \| 0.001991308 \| 0.007 \| 3052 \| \| SANSOM_APC_TARGETS_DN \| 338 \| 0.629615 \| 2.569468 \| <0.001 \| 0.001659423 \| 0.007 \| 7717 \| \| TAKEDA_TARGETS_OF_NUP98_HOXA9_FUSION_8D_UP \| 156 \| 0.679870 \| 2.565164 \| <0.001 \| 0.001557124 \| 0.007 \| 5326 \| \| DIAZ_CHRONIC_MEYLOGENOUS_LEUKEMIA_DN \| 116 \| 0.733083 \| 2.553103 \| <0.001 \| 0.001495315 \| 0.008 \| 4782 \| \| TAKEDA_TARGETS_OF_NUP98_HOXA9_FUSION_16D_UP \| 176 \| 0.678762 \| 2.548561 \| <0.001 \| 0.001538688 \| 0.008 \| 5824 \| \| TARTE_PLASMA_CELL_VS_PLASMABLAST_UP \| 393 \| 0.572521 \| 2.546684 \| <0.001 \| 0.001384819 \| 0.008 \| 9690 \| \| BYSTROEM_CORRELATED_WITH_IL5_UP \| 38 \| 0.637549 \| 2.539649 \| <0.001 \| 0.001515569 \| 0.009 \| 3769 \| \| BOYLAN_MULTIPLE_MYELOMA_PCA1_UP \| 120 \| 0.706335 \| 2.531416 \| <0.001 \| 0.00157241 \| 0.011 \| 4881 \| \| BOYLAN_MULTIPLE_MYELOMA_C_D_DN \| 277 \| 0.648445 \| 2.530999 \| <0.001 \| 0.001451455 \| 0.011 \| 5996 \| \| GAL_LEUKEMIC_STEM_CELL_DN \| 233 \| 0.675541 \| 2.529284 \| <0.001 \| 0.00134778 \| 0.011 \| 3418 \| \| RODWELL_AGING_KIDNEY_UP \| 492 \| 0.732869 \| 2.529191 \| <0.001 \| 0.001257928 \| 0.011 \| 6131 \| \| REACTOME_LEISHMANIA_INFECTION \| 306 \| 0.662593 \| 2.528263 \| <0.001 \| 0.001179307 \| 0.011 \| 3728 \| \| HELLER_SILENCED_BY_METHYLATION_UP \| 277 \| 0.708567 \| 2.526998 \| <0.001 \| 0.001109936 \| 0.011 \| 5607 \| \| BOSCO_TH1_CYTOTOXIC_MODULE \| 113 \| 0.796188 \| 2.522422 \| <0.001 \| 0.001100351 \| 0.011 \| 3797 \| \| REACTOME_COSTIMULATION_BY_THE_CD28_FAMILY \| 72 \| 0.823120 \| 2.521349 \| <0.001 \| 0.001042438 \| 0.011 \| 3749 \| \| TAKEDA_TARGETS_OF_NUP98_HOXA9_FUSION_8D_DN \| 196 \| 0.689991 \| 2.520701 \| <0.001 \| 0.001084599 \| 0.011 \| 3777 \| \| REACTOME_DDX58_IFIH1_MEDIATED_INDUCTION_OF_INTERFERON_ALPHA_BETA \| 78 \| 0.661094 \| 2.514571 \| <0.001 \| 0.001302098 \| 0.011 \| 6125 \| \| CHYLA_CBFA2T3_TARGETS_UP \| 376 \| 0.613837 \| 2.514362 \| <0.001 \| 0.001242912 \| 0.011 \| 8312 \| \| FULCHER_INFLAMMATORY_RESPONSE_LECTIN_VS_LPS_DN \| 442 \| 0.734541 \| 2.512409 \| <0.001 \| 0.001311614 \| 0.011 \| 5213 \| \| TAKEDA_TARGETS_OF_NUP98_HOXA9_FUSION_3D_UP \| 178 \| 0.718908 \| 2.510661 \| <0.001 \| 0.001256964 \| 0.011 \| 3969 \| \| KEGG_ANTIGEN_PROCESSING_AND_PRESENTATION \| 81 \| 0.791087 \| 2.505254 \| <0.001 \| 0.001357539 \| 0.011 \| 3859 \| \| KUMAR_TARGETS_OF_MLL_AF9_FUSION \| 401 \| 0.565433 \| 2.500137 \| <0.001 \| 0.001386321 \| 0.013 \| 8152 \| \| GAVIN_FOXP3_TARGETS_CLUSTER_P3 \| 160 \| 0.639525 \| 2.499157 \| <0.001 \| 0.001334976 \| 0.013 \| 5239 \| \| RUTELLA_RESPONSE_TO_HGF_DN \| 233 \| 0.663557 \| 2.497982 \| <0.001 \| 0.001320989 \| 0.013 \| 6136 \| \| REACTOME_NEUTROPHIL_DEGRANULATION \| 476 \| 0.627885 \| 2.496501 \| <0.001 \| 0.001377565 \| 0.014 \| 9390 \| \| TONKS_TARGETS_OF_RUNX1_RUNX1T1_FUSION_ERYTHROCYTE_UP \| 157 \| 0.703123 \| 2.494256 \| <0.001 \| 0.001370993 \| 0.015 \| 5747 \| \| KEGG_CYTOKINE_CYTOKINE_RECEPTOR_INTERACTION \| 264 \| 0.675935 \| 2.493165 \| <0.001 \| 0.001326768 \| 0.015 \| 4172 \| \| GRAESSMANN_RESPONSE_TO_MC_AND_SERUM_DEPRIVATION_UP \| 211 \| 0.648820 \| 2.490257 \| <0.001 \| 0.001285306 \| 0.015 \| 3754 \| \| KEGG_CHEMOKINE_SIGNALING_PATHWAY \| 188 \| 0.653777 \| 2.486125 \| <0.001 \| 0.001306142 \| 0.016 \| 5601 \| \| REACTOME_TCR_SIGNALING \| 122 \| 0.727348 \| 2.485725 \| <0.001 \| 0.001295297 \| 0.016 \| 2476 \| \| KEGG_VIRAL_MYOCARDITIS \| 68 \| 0.806137 \| 2.483349 \| <0.001 \| 0.001287142 \| 0.017 \| 2332 \| \| RUTELLA_RESPONSE_TO_CSF2RB_AND_IL4_DN \| 315 \| 0.702068 \| 2.473457 \| <0.001 \| 0.001309672 \| 0.018 \| 6924 \| \| THUM_SYSTOLIC_HEART_FAILURE_UP \| 424 \| 0.659648 \| 2.469021 \| <0.001 \| 0.001558916 \| 0.02 \| 4544 \| \| KEGG_T_CELL_RECEPTOR_SIGNALING_PATHWAY \| 108 \| 0.674858 \| 2.465259 \| <0.001 \| 0.001634192 \| 0.021 \| 3508 \| \| TAKEDA_TARGETS_OF_NUP98_HOXA9_FUSION_10D_UP \| 190 \| 0.651462 \| 2.461057 \| <0.001 \| 0.001714145 \| 0.022 \| 3178 \| \| LEE_DIFFERENTIATING_T_LYMPHOCYTE \| 196 \| 0.755078 \| 2.458102 \| <0.001 \| 0.001794095 \| 0.022 \| 3607 \| \| RASHI_RESPONSE_TO_IONIZING_RADIATION_6 \| 83 \| 0.778249 \| 2.457823 \| <0.001 \| 0.001750336 \| 0.022 \| 3220 \| \| SMID_BREAST_CANCER_NORMAL_LIKE_UP \| 481 \| 0.744527 \| 2.457377 \| <0.001 \| 0.001708661 \| 0.022 \| 3520 \| \| REACTOME_INTERFERON_GAMMA_SIGNALING \| 91 \| 0.829733 \| 2.445879 \| <0.001 \| 0.001893483 \| 0.022 \| 4462 \| \| FURUKAWA_DUSP6_TARGETS_PCI35_UP \| 71 \| 0.716938 \| 2.444694 \| <0.001 \| 0.001850449 \| 0.022 \| 6488 \| \| REACTOME_MHC_CLASS_II_ANTIGEN_PRESENTATION \| 123 \| 0.594468 \| 2.444532 \| <0.001 \| 0.001831238 \| 0.022 \| 4199 \| \| BASSO_CD40_SIGNALING_DN \| 70 \| 0.679997 \| 2.443634 \| <0.001 \| 0.001812951 \| 0.023 \| 4431 \| \| ONO_AML1_TARGETS_DN \| 40 \| 0.778563 \| 2.441208 \| <0.001 \| 0.001842326 \| 0.023 \| 4462 \| \| WANG_BARRETTS_ESOPHAGUS_UP \| 52 \| 0.662680 \| 2.440601 \| <0.001 \| 0.001803945 \| 0.023 \| 8498 \| \| JAATINEN_HEMATOPOIETIC_STEM_CELL_DN \| 234 \| 0.743576 \| 2.440379 \| <0.001 \| 0.001767129 \| 0.023 \| 3332 \| \| VALK_AML_WITH_CEBPA \| 37 \| 0.735952 \| 2.439888 \| <0.001 \| 0.001731787 \| 0.023 \| 5994 \| \| BOYLAN_MULTIPLE_MYELOMA_D_DN \| 78 \| 0.672605 \| 2.438856 \| <0.001 \| 0.001716327 \| 0.023 \| 3029 \| \| BROWN_MYELOID_CELL_DEVELOPMENT_UP \| 160 \| 0.718314 \| 2.435458 \| <0.001 \| 0.001737515 \| 0.023 \| 4791 \| \| POOLA_INVASIVE_BREAST_CANCER_UP \| 291 \| 0.765023 \| 2.434749 \| <0.001 \| 0.001704732 \| 0.023 \| 3197 \| \| VERHAAK_AML_WITH_NPM1_MUTATED_DN \| 246 \| 0.620913 \| 2.432730 \| <0.001 \| 0.001673163 \| 0.023 \| 5775 \| \| REACTOME_ANTIGEN_PROCESSING_CROSS_PRESENTATION \| 99 \| 0.761181 \| 2.429279 \| <0.001 \| 0.001663205 \| 0.023 \| 2929 \| \| PID_IL12_2PATHWAY \| 63 \| 0.826856 \| 2.428990 \| <0.001 \| 0.001633505 \| 0.023 \| 2784 \| \| LEE_AGING_CEREBELLUM_UP \| 79 \| 0.694750 \| 2.424883 \| <0.001 \| 0.001641832 \| 0.024 \| 3501 \| \| WALLACE_PROSTATE_CANCER_RACE_UP \| 299 \| 0.823900 \| 2.424060 \| <0.001 \| 0.001613525 \| 0.024 \| 3933 \| \| KEGG_TOLL_LIKE_RECEPTOR_SIGNALING_PATHWAY \| 102 \| 0.682312 \| 2.419383 \| <0.001 \| 0.001695325 \| 0.027 \| 5493 \| \| ZHENG_IL22_SIGNALING_UP \| 58 \| 0.724917 \| 2.417960 \| <0.001 \| 0.001703175 \| 0.027 \| 3946 \| \| WORSCHECH_TUMOR_EVASION_AND_TOLEROGENICITY_UP \| 32 \| 0.801330 \| 2.417251 \| <0.001 \| 0.001690719 \| 0.027 \| 2576 \| \| YU_MYC_TARGETS_DN \| 58 \| 0.783893 \| 2.413766 \| <0.001 \| 0.001760922 \| 0.03 \| 3720 \| \| REACTOME_INTERFERON_ALPHA_BETA_SIGNALING \| 70 \| 0.834504 \| 2.411863 \| <0.001 \| 0.001783209 \| 0.032 \| 4621 \| \| ICHIBA_GRAFT_VERSUS_HOST_DISEASE_35D_UP \| 143 \| 0.812622 \| 2.409297 \| <0.001 \| 0.001755346 \| 0.032 \| 3066 \| \| LI_INDUCED_T_TO_NATURAL_KILLER_UP \| 313 \| 0.640865 \| 2.406764 \| <0.001 \| 0.001789179 \| 0.032 \| 9152 \| \| LEIN_ASTROCYTE_MARKERS \| 42 \| 0.668772 \| 2.405723 \| <0.001 \| 0.00176207 \| 0.032 \| 2690 \| \| BIDUS_METASTASIS_DN \| 149 \| 0.662245 \| 2.402060 \| <0.001 \| 0.001809092 \| 0.033 \| 11260 \| \| KATSANOU_ELAVL1_TARGETS_UP \| 157 \| 0.615236 \| 2.397217 \| <0.001 \| 0.001926239 \| 0.036 \| 5524 \| \| LIU_SMARCA4_TARGETS \| 51 \| 0.693828 \| 2.396749 \| <0.001 \| 0.001946417 \| 0.037 \| 5035 \| \| WUNDER_INFLAMMATORY_RESPONSE_AND_CHOLESTEROL_UP \| 60 \| 0.852701 \| 2.393927 \| <0.001 \| 0.001992586 \| 0.038 \| 1844 \| \| ALTEMEIER_RESPONSE_TO_LPS_WITH_MECHANICAL_VENTILATION \| 119 \| 0.820209 \| 2.393507 \| <0.001 \| 0.001980787 \| 0.038 \| 3670 \| \| VILIMAS_NOTCH1_TARGETS_UP \| 51 \| 0.847073 \| 2.393048 \| <0.001 \| 0.001953276 \| 0.038 \| 1609 \| \| SERVITJA_ISLET_HNF1A_TARGETS_UP \| 167 \| 0.570132 \| 2.392365 \| <0.001 \| 0.001940077 \| 0.038 \| 9520 \| \| REACTOME_RESPONSE_TO_ELEVATED_PLATELET_CYTOSOLIC_CA2 \| 132 \| 0.548487 \| 2.392242 \| <0.001 \| 0.00191386 \| 0.038 \| 12644 \| \| JOHNSTONE_PARVB_TARGETS_2_UP \| 142 \| 0.610749 \| 2.390199 \| <0.001 \| 0.001932435 \| 0.038 \| 8815 \| \| CHIARETTI_ACUTE_LYMPHOBLASTIC_LEUKEMIA_ZAP70 \| 66 \| 0.662825 \| 2.389696 \| <0.001 \| 0.00192254 \| 0.038 \| 6447 \| \| ODONNELL_TARGETS_OF_MYC_AND_TFRC_UP \| 80 \| 0.771600 \| 2.388233 \| <0.001 \| 0.001937391 \| 0.038 \| 5167 \| \| MALONEY_RESPONSE_TO_17AAG_UP \| 40 \| 0.691014 \| 2.386782 \| <0.001 \| 0.001912553 \| 0.038 \| 4468 \| \| WANG_ESOPHAGUS_CANCER_VS_NORMAL_UP \| 119 \| 0.655350 \| 2.381753 \| <0.001 \| 0.00211058 \| 0.038 \| 4752 \| \| MORI_MATURE_B_LYMPHOCYTE_UP \| 94 \| 0.715372 \| 2.381593 \| <0.001 \| 0.002084198 \| 0.038 \| 6234 \| \| REACTOME_ANTI_INFLAMMATORY_RESPONSE_FAVOURING_LEISHMANIA_PARASITE_INFECTION \| 223 \| 0.680638 \| 2.381014 \| <0.001 \| 0.002058467 \| 0.038 \| 3728 \| \| WORSCHECH_TUMOR_REJECTION_UP \| 60 \| 0.778137 \| 2.379979 \| <0.001 \| 0.002045641 \| 0.038 \| 5880 \| \| KEGG_SYSTEMIC_LUPUS_ERYTHEMATOSUS \| 55 \| 0.885746 \| 2.379560 \| <0.001 \| 0.002055079 \| 0.038 \| 1656 \| \| QI_PLASMACYTOMA_UP \| 254 \| 0.732403 \| 2.379072 \| <0.001 \| 0.002056167 \| 0.038 \| 4119 \| \| REACTOME_CD28_CO_STIMULATION \| 33 \| 0.703720 \| 2.378055 \| <0.001 \| 0.002043005 \| 0.038 \| 3749 \| \| BORCZUK_MALIGNANT_MESOTHELIOMA_DN \| 105 \| 0.626108 \| 2.375490 \| <0.001 \| 0.002075658 \| 0.039 \| 8841 \| \| HADDAD_T_LYMPHOCYTE_AND_NK_PROGENITOR_DN \| 64 \| 0.751939 \| 2.374795 \| <0.001 \| 0.002064285 \| 0.04 \| 4591 \| \| KLEIN_TARGETS_OF_BCR_ABL1_FUSION \| 42 \| 0.806166 \| 2.374603 \| <0.001 \| 0.002040827 \| 0.04 \| 5558 \| \| LIAN_LIPA_TARGETS_6M \| 81 \| 0.815298 \| 2.367018 \| <0.001 \| 0.002223773 \| 0.044 \| 4067 \| \| KEGG_COMPLEMENT_AND_COAGULATION_CASCADES \| 69 \| 0.691770 \| 2.366687 \| <0.001 \| 0.002199065 \| 0.044 \| 5717 \| \| MA_MYELOID_DIFFERENTIATION_DN \| 42 \| 0.657963 \| 2.366011 \| 0.001980198 \| 0.002186993 \| 0.044 \| 3029 \| \| FERRANDO_T_ALL_WITH_MLL_ENL_FUSION_UP \| 89 \| 0.655085 \| 2.364533 \| <0.001 \| 0.002184192 \| 0.044 \| 4578 \| \| KEGG_AUTOIMMUNE_THYROID_DISEASE \| 50 \| 0.839566 \| 2.364494 \| <0.001 \| 0.002160706 \| 0.044 \| 1812 \| \| LEE_EARLY_T_LYMPHOCYTE_DN \| 58 \| 0.767996 \| 2.363045 \| <0.001 \| 0.002175947 \| 0.046 \| 4050 \| \| PID_CD8_TCR_DOWNSTREAM_PATHWAY \| 65 \| 0.670251 \| 2.362744 \| <0.001 \| 0.002163624 \| 0.046 \| 2784 \| \| CHEN_ETV5_TARGETS_SERTOLI \| 25 \| 0.824578 \| 2.360130 \| <0.001 \| 0.002183571 \| 0.046 \| 3127 \| \| HOWLIN_CITED1_TARGETS_1_UP \| 34 \| 0.689604 \| 2.359161 \| <0.001 \| 0.002171034 \| 0.046 \| 4754 \| \| WANG_RESPONSE_TO_GSK3_INHIBITOR_SB216763_UP \| 387 \| 0.617081 \| 2.358109 \| <0.001 \| 0.002158942 \| 0.046 \| 8387 \| \| NEMETH_INFLAMMATORY_RESPONSE_LPS_UP \| 80 \| 0.748731 \| 2.358031 \| <0.001 \| 0.002137134 \| 0.046 \| 4973 \| \| VERHAAK_AML_WITH_NPM1_MUTATED_UP \| 178 \| 0.741647 \| 2.357670 \| <0.001 \| 0.002134619 \| 0.046 \| 3977 \| \| GO_NEGATIVE_REGULATION_OF_MULTI_ORGANISM_PROCESS \| 222 \| 0.689291 \| 2.746925 \| <0.001 \| <0.001 \| 0 \| 4782 \| \| GO_NEGATIVE_REGULATION_OF_IMMUNE_RESPONSE \| 152 \| 0.710013 \| 2.707047 \| <0.001 \| <0.001 \| 0.001 \| 2811 \| \| GO_POSITIVE_REGULATION_OF_CYTOKINE_PRODUCTION \| 461 \| 0.676213 \| 2.698461 \| <0.001 \| <0.001 \| 0.001 \| 3854 \| \| GO_NEGATIVE_REGULATION_OF_IMMUNE_EFFECTOR_PROCESS \| 120 \| 0.717291 \| 2.691616 \| <0.001 \| <0.001 \| 0.001 \| 3298 \| \| GO_MODIFICATION_OF_MORPHOLOGY_OR_PHYSIOLOGY_OF_OTHER_ORGANISM \| 164 \| 0.576143 \| 2.670927 \| <0.001 \| <0.001 \| 0.002 \| 6296 \| \| GO_LEUKOCYTE_PROLIFERATION \| 299 \| 0.708344 \| 2.655489 \| <0.001 \| <0.001 \| 0.002 \| 2527 \| \| GO_POSITIVE_REGULATION_OF_RESPONSE_TO_BIOTIC_STIMULUS \| 396 \| 0.608999 \| 2.655097 \| <0.001 \| <0.001 \| 0.002 \| 4085 \| \| GO_NEGATIVE_REGULATION_OF_RESPONSE_TO_BIOTIC_STIMULUS \| 95 \| 0.713606 \| 2.650914 \| <0.001 \| <0.001 \| 0.002 \| 3769 \| \| GO_NEGATIVE_REGULATION_OF_CYTOKINE_PRODUCTION \| 294 \| 0.656026 \| 2.647546 \| <0.001 \| <0.001 \| 0.002 \| 6265 \| \| GO_T_CELL_RECEPTOR_SIGNALING_PATHWAY \| 199 \| 0.710240 \| 2.638808 \| <0.001 \| <0.001 \| 0.002 \| 5649 \| \| GO_NEGATIVE_REGULATION_OF_INNATE_IMMUNE_RESPONSE \| 59 \| 0.760511 \| 2.634029 \| <0.001 \| <0.001 \| 0.002 \| 3769 \| \| GO_REGULATION_OF_LYMPHOCYTE_ACTIVATION \| 485 \| 0.733488 \| 2.630806 \| <0.001 \| <0.001 \| 0.002 \| 3809 \| \| GO_REGULATION_OF_IMMUNE_EFFECTOR_PROCESS \| 451 \| 0.718845 \| 2.630652 \| <0.001 \| <0.001 \| 0.002 \| 3387 \| \| GO_RESPONSE_TO_VIRUS \| 324 \| 0.662280 \| 2.621561 \| <0.001 \| <0.001 \| 0.002 \| 6274 \| \| GO_NEGATIVE_REGULATION_OF_DEFENSE_RESPONSE \| 234 \| 0.625154 \| 2.620051 \| <0.001 \| <0.001 \| 0.002 \| 6265 \| \| GO_ACTIVATION_OF_INNATE_IMMUNE_RESPONSE \| 318 \| 0.604675 \| 2.617731 \| <0.001 \| <0.001 \| 0.002 \| 6265 \| \| GO_DEFENSE_RESPONSE_TO_VIRUS \| 241 \| 0.689585 \| 2.613030 \| <0.001 \| <0.001 \| 0.002 \| 4621 \| \| GO_RESPONSE_TO_TYPE_I_INTERFERON \| 95 \| 0.792491 \| 2.611205 \| <0.001 \| <0.001 \| 0.002 \| 4621 \| \| GO_EXTERNAL_SIDE_OF_PLASMA_MEMBRANE \| 384 \| 0.729220 \| 2.607964 \| <0.001 \| <0.001 \| 0.002 \| 3221 \| \| GO_IMMUNE_RESPONSE_REGULATING_CELL_SURFACE_RECEPTOR_SIGNALING_PATHWAY \| 491 \| 0.735087 \| 2.597068 \| <0.001 \| <0.001 \| 0.003 \| 3769 \| \| GO_CYTOKINE_SECRETION \| 240 \| 0.690713 \| 2.595766 \| <0.001 \| <0.001 \| 0.003 \| 6411 \| \| GO_LEUKOCYTE_MIGRATION \| 493 \| 0.707674 \| 2.594064 \| <0.001 \| <0.001 \| 0.004 \| 6990 \| \| GO_REGULATION_OF_LEUKOCYTE_PROLIFERATION \| 223 \| 0.725917 \| 2.592765 \| <0.001 \| <0.001 \| 0.004 \| 2527 \| \| GO_T_CELL_PROLIFERATION \| 185 \| 0.734466 \| 2.588172 \| <0.001 \| <0.001 \| 0.004 \| 2284 \| \| GO_REGULATION_OF_ADAPTIVE_IMMUNE_RESPONSE \| 159 \| 0.735797 \| 2.588117 \| <0.001 \| <0.001 \| 0.004 \| 3375 \| \| GO_INFLAMMATORY_RESPONSE_TO_ANTIGENIC_STIMULUS \| 50 \| 0.758140 \| 2.583443 \| <0.001 \| <0.001 \| 0.004 \| 2913 \| \| GO_DEFENSE_RESPONSE_TO_BACTERIUM \| 315 \| 0.689199 \| 2.583205 \| <0.001 \| <0.001 \| 0.004 \| 3500 \| \| GO_T_CELL_ACTIVATION \| 466 \| 0.680100 \| 2.582378 \| <0.001 \| <0.001 \| 0.004 \| 6356 \| \| GO_NEGATIVE_REGULATION_OF_LYMPHOCYTE_ACTIVATION \| 149 \| 0.707219 \| 2.580809 \| <0.001 \| <0.001 \| 0.004 \| 7951 \| \| GO_RESPONSE_TO_INTERFERON_GAMMA \| 193 \| 0.786241 \| 2.576339 \| <0.001 \| <0.001 \| 0.004 \| 4524 \| \| GO_LEUKOCYTE_APOPTOTIC_PROCESS \| 107 \| 0.700765 \| 2.572665 \| <0.001 \| <0.001 \| 0.004 \| 4100 \| \| GO_LYMPHOCYTE_ACTIVATION_INVOLVED_IN_IMMUNE_RESPONSE \| 181 \| 0.668987 \| 2.572426 \| <0.001 \| <0.001 \| 0.004 \| 6990 \| \| GO_REGULATION_OF_VIRAL_LIFE_CYCLE \| 145 \| 0.683724 \| 2.571509 \| <0.001 \| <0.001 \| 0.004 \| 4671 \| \| GO_NEGATIVE_REGULATION_OF_CELL_ACTIVATION \| 201 \| 0.692727 \| 2.570654 \| <0.001 \| <0.001 \| 0.004 \| 7590 \| \| GO_HUMORAL_IMMUNE_RESPONSE \| 339 \| 0.746483 \| 2.567419 \| <0.001 \| <0.001 \| 0.004 \| 3758 \| \| GO_NEGATIVE_REGULATION_OF_IMMUNE_SYSTEM_PROCESS \| 452 \| 0.629183 \| 2.565328 \| <0.001 \| <0.001 \| 0.004 \| 6356 \| \| GO_LYMPHOCYTE_APOPTOTIC_PROCESS \| 72 \| 0.707942 \| 2.560437 \| <0.001 \| <0.001 \| 0.004 \| 5932 \| \| GO_REGULATION_OF_CELL_CELL_ADHESION \| 406 \| 0.659056 \| 2.557480 \| <0.001 \| <0.001 \| 0.004 \| 6990 \| \| GO_NEGATIVE_REGULATION_OF_CELL_CELL_ADHESION \| 181 \| 0.639029 \| 2.556952 \| <0.001 \| <0.001 \| 0.004 \| 7590 \| \| GO_REGULATION_OF_SYMBIOSIS_ENCOMPASSING_MUTUALISM_THROUGH_PARASITISM \| 221 \| 0.619331 \| 2.556215 \| <0.001 \| <0.001 \| 0.004 \| 4671 \| \| GO_REGULATION_OF_T_CELL_ACTIVATION \| 316 \| 0.716745 \| 2.556031 \| <0.001 \| <0.001 \| 0.004 \| 3809 \| \| GO_NIK_NF_KAPPAB_SIGNALING \| 177 \| 0.609334 \| 2.553393 \| <0.001 \| <0.001 \| 0.004 \| 5032 \| \| GO_IMMUNE_RECEPTOR_ACTIVITY \| 126 \| 0.780947 \| 2.553217 \| <0.001 \| <0.001 \| 0.004 \| 2811 \| \| GO_POSITIVE_REGULATION_OF_CELL_ACTIVATION \| 383 \| 0.756047 \| 2.553135 \| <0.001 \| <0.001 \| 0.004 \| 3538 \| \| GO_LYMPHOCYTE_MEDIATED_IMMUNITY \| 347 \| 0.777852 \| 2.552142 \| <0.001 \| <0.001 \| 0.004 \| 4269 \| \| GO_NEGATIVE_REGULATION_OF_LEUKOCYTE_CELL_CELL_ADHESION \| 128 \| 0.689415 \| 2.551729 \| <0.001 \| <0.001 \| 0.004 \| 7590 \| \| GO_REGULATION_OF_LYMPHOCYTE_MEDIATED_IMMUNITY \| 147 \| 0.713138 \| 2.551642 \| <0.001 \| <0.001 \| 0.004 \| 5824 \| \| GO_NEGATIVE_REGULATION_OF_VIRAL_PROCESS \| 100 \| 0.733746 \| 2.551467 \| <0.001 \| <0.001 \| 0.004 \| 4671 \| \| GO_BLOOD_MICROPARTICLE \| 145 \| 0.697005 \| 2.549641 \| <0.001 \| <0.001 \| 0.004 \| 3859 \| \| GO_ADAPTIVE_IMMUNE_RESPONSE_BASED_ON_SOMATIC_RECOMBINATION_OF_IMMUNE_RECEPTORS_BUILT_FROM_IMMUNOGLOBULIN_SUPERFAMILY_DOMAINS \| 347 \| 0.788855 \| 2.546592 \| <0.001 \| <0.001 \| 0.004 \| 3563 \| \| GO_REGULATION_OF_LEUKOCYTE_MEDIATED_IMMUNITY \| 198 \| 0.716399 \| 2.546547 \| <0.001 \| <0.001 \| 0.004 \| 3375 \| \| GO_ANTIGEN_PROCESSING_AND_PRESENTATION \| 224 \| 0.697155 \| 2.545526 \| <0.001 \| <0.001 \| 0.004 \| 2929 \| \| GO_TYPE_I_INTERFERON_PRODUCTION \| 126 \| 0.709380 \| 2.540976 \| <0.001 \| <0.001 \| 0.004 \| 2495 \| \| GO_VESICLE_LUMEN \| 326 \| 0.579201 \| 2.539536 \| <0.001 \| <0.001 \| 0.004 \| 9520 \| \| GO_INTERLEUKIN_1_PRODUCTION \| 114 \| 0.715253 \| 2.537283 \| <0.001 \| <0.001 \| 0.004 \| 3143 \| \| GO_NEGATIVE_REGULATION_OF_RESPONSE_TO_EXTERNAL_STIMULUS \| 392 \| 0.573433 \| 2.536751 \| <0.001 \| <0.001 \| 0.004 \| 6317 \| \| GO_REGULATION_OF_LEUKOCYTE_APOPTOTIC_PROCESS \| 85 \| 0.733665 \| 2.535221 \| <0.001 \| <0.001 \| 0.004 \| 3375 \| \| GO_ANTIGEN_RECEPTOR_MEDIATED_SIGNALING_PATHWAY \| 303 \| 0.762518 \| 2.531768 \| <0.001 \| <0.001 \| 0.004 \| 3769 \| \| GO_REGULATION_OF_LEUKOCYTE_DIFFERENTIATION \| 274 \| 0.674351 \| 2.531150 \| <0.001 \| <0.001 \| 0.004 \| 3418 \| \| GO_REGULATION_OF_LYMPHOCYTE_DIFFERENTIATION \| 170 \| 0.697065 \| 2.529691 \| <0.001 \| <0.001 \| 0.005 \| 3418 \| \| GO_LEUKOCYTE_CELL_CELL_ADHESION \| 338 \| 0.700925 \| 2.528724 \| <0.001 \| <0.001 \| 0.005 \| 6984 \| \| GO_POSITIVE_REGULATION_OF_LEUKOCYTE_PROLIFERATION \| 139 \| 0.741416 \| 2.524710 \| <0.001 \| <0.001 \| 0.006 \| 2267 \| \| GO_B_CELL_ACTIVATION \| 306 \| 0.712614 \| 2.524299 \| <0.001 \| <0.001 \| 0.006 \| 4844 \| \| GO_POSITIVE_REGULATION_OF_INTERLEUKIN_1_PRODUCTION \| 59 \| 0.753600 \| 2.523953 \| <0.001 \| <0.001 \| 0.007 \| 3018 \| \| GO_LYMPHOCYTE_DIFFERENTIATION \| 353 \| 0.643801 \| 2.522859 \| <0.001 \| <0.001 \| 0.007 \| 6234 \| \| GO_T_CELL_APOPTOTIC_PROCESS \| 49 \| 0.717315 \| 2.520042 \| <0.001 \| <0.001 \| 0.007 \| 5573 \| \| GO_NEGATIVE_REGULATION_OF_LYMPHOCYTE_MEDIATED_IMMUNITY \| 39 \| 0.820756 \| 2.513034 \| <0.001 \| <0.001 \| 0.007 \| 2811 \| \| GO_RESPONSE_TO_MOLECULE_OF_BACTERIAL_ORIGIN \| 349 \| 0.641162 \| 2.510753 \| <0.001 \| <0.001 \| 0.007 \| 6271 \| \| GO_POSITIVE_REGULATION_OF_CYTOKINE_SECRETION \| 139 \| 0.691322 \| 2.503657 \| <0.001 \| <0.001 \| 0.007 \| 4085 \| \| GO_INTERLEUKIN_1_BETA_PRODUCTION \| 100 \| 0.727088 \| 2.501568 \| <0.001 \| <0.001 \| 0.007 \| 5876 \| \| GO_CELL_KILLING \| 166 \| 0.671415 \| 2.501469 \| <0.001 \| <0.001 \| 0.007 \| 7241 \| \| GO_CYTOKINE_METABOLIC_PROCESS \| 123 \| 0.723285 \| 2.497473 \| <0.001 \| <0.001 \| 0.007 \| 8605 \| \| GO_NEGATIVE_REGULATION_OF_LEUKOCYTE_MEDIATED_IMMUNITY \| 49 \| 0.817710 \| 2.497290 \| <0.001 \| <0.001 \| 0.007 \| 2811 \| \| GO_INTERLEUKIN_6_PRODUCTION \| 162 \| 0.713542 \| 2.496688 \| <0.001 \| <0.001 \| 0.007 \| 5876 \| \| GO_REGULATION_OF_PRODUCTION_OF_MOLECULAR_MEDIATOR_OF_IMMUNE_RESPONSE \| 141 \| 0.658589 \| 2.495244 \| <0.001 \| <0.001 \| 0.007 \| 4269 \| \| GO_REGULATION_OF_T_CELL_MEDIATED_IMMUNITY \| 71 \| 0.751599 \| 2.494121 \| <0.001 \| <0.001 \| 0.007 \| 3881 \| \| GO_T_CELL_MEDIATED_IMMUNITY \| 100 \| 0.725649 \| 2.491381 \| <0.001 \| <0.001 \| 0.007 \| 4054 \| \| GO_NEGATIVE_REGULATION_OF_LEUKOCYTE_PROLIFERATION \| 79 \| 0.740766 \| 2.490227 \| <0.001 \| <0.001 \| 0.007 \| 5607 \| \| GO_NEGATIVE_REGULATION_OF_VIRAL_LIFE_CYCLE \| 84 \| 0.743957 \| 2.488633 \| <0.001 \| <0.001 \| 0.007 \| 4671 \| \| GO_INTERFERON_GAMMA_PRODUCTION \| 110 \| 0.766935 \| 2.488393 \| <0.001 \| <0.001 \| 0.007 \| 2874 \| \| GO_TUMOR_NECROSIS_FACTOR_SUPERFAMILY_CYTOKINE_PRODUCTION \| 170 \| 0.690819 \| 2.486736 \| <0.001 \| <0.001 \| 0.008 \| 6906 \| \| GO_REGULATION_OF_T_CELL_DIFFERENTIATION \| 139 \| 0.708970 \| 2.480714 \| <0.001 \| <0.001 \| 0.009 \| 3769 \| \| GO_ENDOCYTIC_VESICLE_MEMBRANE \| 158 \| 0.647744 \| 2.476561 \| <0.001 \| <0.001 \| 0.009 \| 4423 \| \| GO_CYTOKINE_RECEPTOR_ACTIVITY \| 97 \| 0.721932 \| 2.473415 \| <0.001 \| <0.001 \| 0.01 \| 2776 \| \| GO_POSITIVE_REGULATION_OF_T_CELL_PROLIFERATION \| 95 \| 0.789628 \| 2.472949 \| <0.001 \| <0.001 \| 0.01 \| 2267 \| \| GO_REGULATION_OF_HEMOPOIESIS \| 446 \| 0.590023 \| 2.470412 \| <0.001 \| <0.001 \| 0.01 \| 8771 \| \| GO_REGULATION_OF_LYMPHOCYTE_APOPTOTIC_PROCESS \| 55 \| 0.750613 \| 2.470110 \| <0.001 \| <0.001 \| 0.01 \| 3375 \| \| GO_REGULATION_OF_INFLAMMATORY_RESPONSE \| 365 \| 0.615089 \| 2.470084 \| <0.001 \| <0.001 \| 0.01 \| 8944 \| \| GO_CELLULAR_RESPONSE_TO_BIOTIC_STIMULUS \| 241 \| 0.657104 \| 2.469909 \| <0.001 \| <0.001 \| 0.01 \| 6271 \| \| GO_T_CELL_DIFFERENTIATION \| 239 \| 0.665117 \| 2.467820 \| <0.001 \| <0.001 \| 0.011 \| 3800 \| \| GO_VIRAL_GENOME_REPLICATION \| 122 \| 0.668656 \| 2.467582 \| <0.001 \| <0.001 \| 0.011 \| 4524 \| \| GO_NEGATIVE_REGULATION_OF_ADAPTIVE_IMMUNE_RESPONSE \| 46 \| 0.765506 \| 2.465477 \| <0.001 \| <0.001 \| 0.011 \| 2811 \| \| GO_POSITIVE_REGULATION_OF_IMMUNE_EFFECTOR_PROCESS \| 211 \| 0.666374 \| 2.465330 \| <0.001 \| <0.001 \| 0.011 \| 3375 \| \| GO_NEGATIVE_REGULATION_OF_T_CELL_PROLIFERATION \| 60 \| 0.736511 \| 2.464119 \| <0.001 \| <0.001 \| 0.011 \| 5607 \| \| GO_CELL_RECOGNITION \| 207 \| 0.678692 \| 2.461794 \| <0.001 \| <0.001 \| 0.011 \| 4207 \| \| GO_NEGATIVE_REGULATION_OF_INFLAMMATORY_RESPONSE \| 164 \| 0.592023 \| 2.460696 \| <0.001 \| <0.001 \| 0.011 \| 6265 \| \| GO_T_CELL_HOMEOSTASIS \| 38 \| 0.740057 \| 2.460674 \| <0.001 \| <0.001 \| 0.011 \| 546 \| \| GO_ENTRY_INTO_HOST \| 133 \| 0.622157 \| 2.459659 \| <0.001 \| <0.001 \| 0.011 \| 6090 \| \| GO_ANTIGEN_PROCESSING_AND_PRESENTATION_OF_PEPTIDE_ANTIGEN \| 187 \| 0.683419 \| 2.459560 \| <0.001 \| <0.001 \| 0.011 \| 2929 \| \| GO_NEGATIVE_REGULATION_OF_SECRETION \| 231 \| 0.572724 \| 2.453197 \| <0.001 \| <0.001 \| 0.012 \| 5864 \| |

GSEA, gene set enrichment analysis; ES, Enrichment score; NES, Normalized enrichment score; NOM p-val, Nominal p value; FDR q-val, False discovery rate q-value; FWER p-val, Familywise-error rate p-value.
